# Dynamic Energy Optimization and Lighting Flexibility Classification for Sustainable Vertical Farming: A Simulation-Based Multi-Scenario Analysis

**DOI:** 10.12688/openreseurope.20847.1

**Published:** 2025-10-16

**Authors:** Chrysovalantis Ketikidis, Petros Dallas, Aristotelis Triantafyllidis, Christina Makri, Panagiotis Grammelis

**Affiliations:** 1 CPERI, Ethniko Kentro Ereunas & Technologikes Anaptyxes, Ptolemaida,, Dytiki Makedonia, 50200, Greece

**Keywords:** Vertical Farming, Lettuce Cultivation, Photovoltaic Integration, TRNSYS 18 Simulation, Energy Optimization, Carbon Footprint

## Abstract

**Background:**

Vertical farming offers a promising solution to food production challenges in urban and climate-constrained regions, yet its high energy demand raises concerns about sustainability.

**Methods:**

This study evaluates the performance and carbon footprint of a pilot vertical farming unit in Northern Greece using TRNSYS 18 simulations and high- resolution environmental data. Forty-eight cultivation scenarios were generated by varying photoperiods, humidity levels, and HVAC setpoints to reflect seasonal Mediterranean conditions. Each scenario was analyzed for energy consumption, grid reliance, photovoltaic sufficiency, and life cycle and CO _2_ emissions. A dynamic crop cycle estimation model was applied to capture seasonal variability and align planting windows with solar energy availability. Performance benchmarking was conducted through a multi-criteria framework introducing the Grid Independence Index, Seasonal Resilience Score, and energy-per-cycle indicators.

**Results:**

The median carbon footprint across scenarios was 3.67kg CO _2_ per kilogram of lettuce (Lactuca Sativa), representing favorable performance compared to conventional methods such as greenhouse cultivation. The analysis demonstrated seasonal fluctuations in photovoltaic sufficiency, with certain scenarios achieving higher autonomy during spring and summer. A Lighting Flexibility Classification was developed to identify the maximum lighting reduction tolerance per scenario without increasing total energy demand, resulting in five flexibility levels and supporting adaptive climate-responsive strategies.

**Conclusions:**

The findings highlight the potential of simulation-based design for optimizing energy use and minimizing environmental impacts in control-environment agriculture. The proposed metrics and classification provide practical tools for improving the resilience and sustainability of vertical farming under Mediterranean conditions.

## Plain Language Summary

This study looks at how vertical farming, growing crops in stacked indoor systems, can become more energy-efficient and environmentally friendly. Using a test facility in Northern Greece, simulation scenarios of 48 lettuce-plantings were produced with different lighting and climate settings. The electricity production was measured, along with calculations of the amount of energy coming from solar panels, and the amount of carbon dioxide (CO _2_) released. This study also examined the best planting periods based on seasonal sunlight, tools to rate how independent the system was from the electricity grid and how flexible the lighting setup can be. Overall, the study shows that vertical farms can reduce their environmental impact with smart planning, especially when renewable energy is used.

## 1. Introduction

The increasing demand for sustainable food production in densely populated urban areas has led to a growing interest in Vertical Farming (VF) systems. These closed-loop, climate-controlled environments enable year-round crop cultivation with minimal land use, reduced water consumption through hydroponic systems, and the elimination of pesticides and herbicides ^1, 2^. Vertical farms can yield significantly higher productivity per square meter compared to traditional open-field agriculture, making them a promising solution for future food security ^3^. However, despite their agronomic advantages, VF systems are characterized by disproportionately high energy demands, primarily attributed to artificial lighting and climate control systems. Lighting alone, mostly provided by LED luminaires, accounts for 60–80% of total electricity consumption in typical VF setups ^4, 5^. This heavy energy burden challenges the economic viability and environmental sustainability of VF, especially in regions with high electricity costs or low renewable energy penetration. To address these challenges, recent research efforts have focused on optimizing energy use through simulation-based design, renewable energy integration, and advanced control strategies. Energy modeling tools offer the ability to evaluate lighting regimes, thermal loads, and storage performance under different operating conditions ^6^. Furthermore, integrating photovoltaic (PV) systems with battery storage and MPPT inverters has been proposed as a potential solution to enhance energy autonomy and reduce reliance on grid electricity ^7, 8^. To maximize the Vertical Farming benefits, the applicant must integrate energy-efficient systems and renewable energy sources to remain competitive against traditional agriculture powered by natural sunlight. J. Pimentel’s study ^6^ explored different strategies for energy optimization to be integrated into urban infrastructure, utilizing a multipored model. The results showed that optimizing resource allocation and operation plans can lead to electricity cost savings of up to 40% daily and 31% annually. A case study in Malaysia assesses solar/hybrid/storage energy solutions, demonstrating that grid-connected solar PV systems can support up to 11.6% of VF energy demand while reducing dependency on utility grids and lowering CO _2_ emissions. This study highlights the economic viability of renewable energy integration through Levelized Cost of Energy (LCOE) analysis ^6^. This study aims to assess the energy efficiency of a VF system under a Mediterranean climatic context through a scenario-based analysis. Using TRNSYS 18, 48 crop cycles are simulated to quantify lighting and HVAC energy demands. Scenarios include different photoperiod strategies and the integration of renewable energy systems. The objective is to identify optimal operational configurations that minimize energy use per unit of harvested biomass, contributing to the broader effort of improving VF sustainability.

## 2. Scope of the study

**Figure 1.**
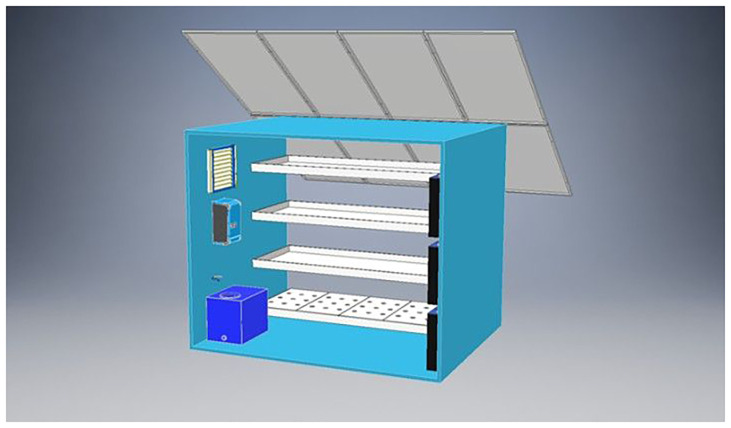
3D visualization of the pilot vertical farming unit.

This study investigates the energy performance and carbon footprint of a fully controlled vertical hydroponic system designed for lettuce cultivation ( Figure 1). The analysis is based on dynamic simulations performed using TRNSYS 18, a transient system simulation tool, under a set of predefined technical configurations and operational conditions. The examined system operates in a Mediterranean area, specifically simulating climate data corresponding to Ptolemaida, Greece. Environmental control includes artificial LED lighting, HVACD systems (Heating, Ventilation, Air Conditioning, and Dehumidification), and automated irrigation via nutrient film technique (NFT) which is an active hydroponic system where plants are grown with their bare roots submerged in a shallow stream of nutrient-rich water that continuously circulates through watertight channels or gullies. This method uses a pump to deliver nutrients and water, while the shallow, oxygenated film ensures plants receive water, nutrients, and oxygen. NFT systems are water-efficient, require less space than other hydroponic methods, and are particularly suited for leafy greens and other fast-growing crops ^1^. All simulations consider a fully enclosed environment, isolated from external natural light. The main focus of the analysis lies in the evaluation of electricity consumption per unit of yield (expressed in kWh/kg), taking into account the impact of different operational scenarios—48 in total—that vary in crop cycle duration, lighting profiles, and environmental parameters. Additionally, the study assesses the sufficiency of an integrated photovoltaic (PV) system with battery storage in covering the system's energy demands, both on an annual and seasonal basis. The carbon footprint is calculated based on energy consumption and regional emission factors, allowing a comparative assessment with conventional farming systems. The potential benefits of dynamic lighting control strategies are also explored, as a means to reduce overall consumption without affecting yield.

This schematic shows the pilot vertical farming unit analyzed in this study, comprising four cultivation shelves with LED lighting and integrated HVAC and nutrient delivery systems. Solar panels are mounted on top to enable partial energy autonomy. The internal layout includes environmental sensors, a nutrient reservoir (bottom left), and control electronics (left wall). The above sketch was provided by the manufacturer.

## 3. Methodology

In order to assess the energy performance and sustainability potential of a vertical farming (VF) system, this study employs a scenario-based dynamic simulation framework that integrates environmental control parameters, crop cycle variation, and renewable energy contributions. The methodological approach is designed to capture the complexity of VF operations by combining system-level energy modeling, crop growth cycle configurations, and photovoltaic energy supply analysis. The analysis unfolds across multiple stages. Initially, a comprehensive set of cultivation scenarios is defined, incorporating variations in cycle duration, lighting regimes, and harvest frequency. These scenarios are then used as input into a detailed simulation environment developed in TRNSYS 18. The model replicates the core subsystems of a VF unit, including lighting, HVACD Systems and other components (pumps, fans, etc.) as presented in Manuscript Tables ( Table 1). Simulations were run in TRNSYS 18 at 15 min timestep. For weather inputs Meteonorm data were used. Type 56 of TRNSYS components was used in order to simulate the ISOBOX (vertical farming unit) as a single zone building, where dehumidification modeled via latent load removal. Lighting is user defined electric load with scenario specific photoperiod (10–16 h). Heat pump/HVAC: performance maps applied with heating/cooling setpoints 12–18 °C/20–28 °C. The system operates with a self-consumption priority, where PV supply is directed first to loads, the to the battery, and finally to the grid.

**Table 1.** VF Equipment Characteristics.

Vertical Farming System Equipment													
*Pumps*		*Battery 2V for our case 24 are used*		*Inverter*		*PV Characteristics*		*Heat Pump Technical Specifications*		*LED Lights*		*Fans*	
Types	Values	Specification	Per Cell	Model	Voltronic Axpert VM III 5000-48	Model Name	LX-430M108H (N-Type Bifacial)	Nominal Cooling Capacity	3.4 kW (1.4–4.0 kW range)	Model	MARSHYDRO HYDRO	Model	PF28080R
Flow Rate (m ^3^/h)	1	Nominal Voltage	2 V	Nominal Power	5000 VA / 5000 W	Cell Type	Monocrystalline, Half-Cut, Bifacial	Nominal Heating Capacity	4.0 kW (1.4–5.2 kW range)	Power Consuption	300W	Dimensions	280 × 280 × 80 mm
Head (m)	45	Capacity C120 (1,85V/cell, 20°C)	605 (Ah)	Output Voltage	230 VAC	Module Efficiency	22.02%	Cooling Power Input	~0.80 kW	PPFD at 30 cm	940 μmol/m ^2^/s	Voltage	230 V AC
Power (kW)	0.55	Capacity C120 (1,80V/cell, 20°C)	422 (Ah)	Output Frequency	50/60 Hz	Nominal Power (Pmax)	430 Wp	Heating Power Input	~0.99 kW	PPFD at 45 cm	780 μmol/m ^2^/s	Power Consumption	130 W
Current (A)	1.6	Recommended Float Voltage	2.23 V	Battery Voltage	48 VDC	Open Circuit Voltage (Voc)	38.93 V	Seasonal Efficiency (SEER/SCOP)	SEER ≈ 8.65 (Cooling), SCOP ≈ 5.10 (Heating)	Spectrum	Full Spectrum (IR+Red+Blue)	Current	0.35 A
Voltage (V)	230	Boost Charge Voltage	2.40 V	Max Charging Current	80 A	Short Circuit Current (Isc)	14.01 A	Nominal EER/COP	EER ≈ 4.23; COP ≈ 4.04	LED Type	Samsung + Epistar Diodes	Speed (RPM)	2,600 rpm
Rotational Speed (rpm)	2850	End-of-Discharge Voltage (C10 rate)	1.80 V	PV Input Voltage Range	60-145 VDC	Maximum Power Voltage (Vmp)	32.65 V	Operating Refrigerant	R-32 (environmentally efficient)	Cooling	Passive aluminum heat	Airflow	1,800–1,980 m ^3^/h (≈ 500–550 L/s)
Impeller Diameter (mm)	140	Internal Resistance	0.64 mΩ	Max PV Array Power	4000 W	Maximum Power Current (Imp)	13.17 A	Airflow (Cooling Mode)	~276–738 m ^3^/h (multiple fan speeds)	Dimming	0–100% knob dimming	Noise Level	68 dBA
Weight (kg)	8.5	Short Circuit Current (each)	3170 A	Max PV Input Current	66 A	NOCT (Nominal Operating Cell Temperature)	45 ± 2 °C	Indoor Unit Noise	Very low (bedroom/office suitable)	Waterproof Rating	IP65	Bearing Type	Ball bearings
		Cycle Life 60% DOD (20°C)	2000 cycles	Efficiency	93% (peak)	Temperature Coefficient (Pmax)	-0.29 %/°C	Controls & Comfort Features	Inverter compressor, intelligent motion sensor, Wi-Fi control	Dimensions	600 × 300 × 70 mm		
		Self-Discharge Rate (20°C)	~2.5% per month	Communication Interfaces	RS232, USB, Wi-Fi (optional), dry contact	Module Dimensions	1722 x 1134 x30 mm	Dimensions/Weight (Indoor)	~778 × 299 × 272 mm; ~14.5 kg	Weight	5.2 kg		
		Recommended Temperature Range	10–30°C					Certifications	EU Label A+++ for both heating & cooling				
		Pole Type	M10										

Subsequently, the developed scenarios were based on the operational setup of a pilot-scale vertical hydroponic unit. This real-world infrastructure provided the necessary technical specifications and boundary conditions for the simulation, including crop cycles, energy consumption patterns, and system sizing. To evaluate the performance and environmental impact of the simulated cases, results were compared against typical values reported in recent literature regarding energy use and carbon emissions across different agricultural practices:

- Open-field agriculture: Minimal energy use (≈0.35 kWh/kg of lettuce), no artificial lighting, but highly weather-dependent and less controllable ^9^.
- Greenhouse cultivation: Moderate energy footprint (≈5.4 kWh/kg), partial environmental control, and supplemental lighting ^1^.
- Standard vertical farming (grid-powered): High energy intensity (≈10–12.5 kWh/kg), dominated by artificial lighting and climate control needs ^5^.
- Optimized hybrid VF systems with PV support: Potential for up to 50% self-sufficiency depending on location, crop scheduling, and storage availability ( 7, 8).

This comparative framework allows the proposed model to be contextualized within the broader landscape of sustainable food production systems. This methodological structure ensures that the simulation captures both the technological intricacies and environmental implications of VF operation, providing a solid foundation for evaluating optimization pathways and energy-efficiency strategies.

In the following section, the simulation framework is presented in more detail, including the system architecture, component modeling, and scenario parameterization.

### 3.1 Cultivation procedure

The selected crop for this analysis is lettuce ( *Lactuca sativa*), a leafy green that has become a benchmark species in vertical farming studies, has high responsiveness to microclimate conditions. It is a diploid species (2n = 18), photoperiod-sensitive, and generally classified as a long-day plant. Ch. Vatistas *et al*. compared the lettuce cultivation between GHs and VFs and presents that VFs are more efficient than GHs in terms of yield per unit area, but require more energy to operate. The experimental plant material, lettuce cultivar ROMANIA (Lactuca sativa), was obtained from Gemma Grow, a certified supplier of planting material within the Greek agricultural sector. The delivery was accompanied by a plant passport with the following specifications: A: Lactuca sativa, B: 02319004, C: 32658CINDEX, D: GR, Lot number: C.

To become more sustainable, VFs must reduce energy demand and develop energy-efficient systems ^10^. Optimal growth has been observed under light intensities with photoperiods of 16 hours. Temperature plays a crucial role as well, with ideal day/night temperature ranges reported between 20–24 °C/16–18 °C. Deviations from these conditions can result in delayed growth, poor head formation, or early bolting ^9^. Photoperiods exceeding 18 hours promote bolting, particularly under elevated temperatures. Optimal growth is achieved at 18–22°C during the light period and 10–15°C during the dark period. Exposure to temperatures above 28°C, especially combined with long photoperiods, increases the likelihood of early flowering and bitterness due to increased sesquiterpene lactones. Table 2 below includes a literature review concerning the conditions for lettuce cultivation.

**Table 2.** Literature review on lettuce cultivation conditions.

Photoperiod	Humidity	Temperature	Duration	Cultivation type	CO2	Ref.
16	60 – 80%	16 – 18 °C	transplanted 20 + 35 days	Vertical Farming (VF)		11
16–20	65%	22 °C	14 + 30	Indoor Controlled Environment Agriculture (CEA)		12
24		23,9	30 days	Hydroponic Yield		13
10	60 – 80	20,5–24	15 + 30	Tempered glass covered greenhouse (GH)		14
12 – 16	60–65%	21.7	29 + 48	Conventional soilbased cultivation (CN) system	11.97 m ^–2^ s ^–1^	15
12 – 16	60–65%	22.3	22 +40.5	Deep water culture (DWC) system	12.95 m ^–2^ s ^–1^	
12 – 16	60–65%	23.3	22 + 47	Nutrient film technique (NFT) system	10.18 m ^–2^ s ^–1^	
12		20 – 22	35			16
18		22 – 28		Directly from the Seeds in Three Separate NTF Hydroponic Systems		17
16 – 20	70–80	22–26 /15–20	30 days			18
	65–75	24/18	19 days after transplantation			19
	60	11	34			20
	60	20	15			
	60	32	14			
	60–70	17– 22	57 days			21

Table 2 presents a comparative overview of reported cultivation parameters for lettuce (Lactuca sativa) from the literature. These include photoperiod (in hours), humidity (%), temperature (°C), and total cultivation duration (in days), along with the cultivation system employed. This dataset serves as a benchmark for evaluating the environmental conditions used in this study's simulation scenarios. Where applicable, CO _2_ photosynthetic photon flux density (PPFD) values are reported.

**Figure 2.**
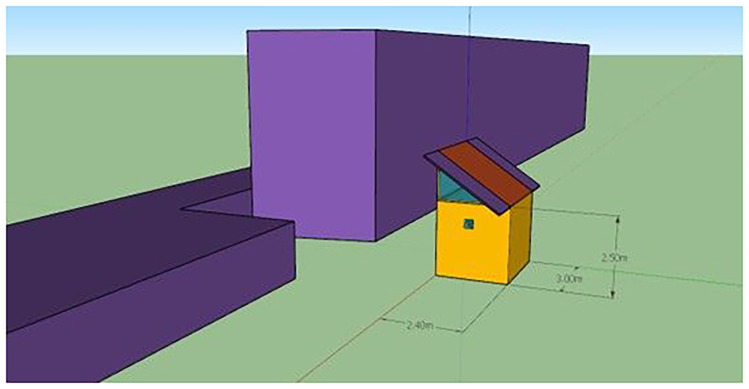
ISOBOX single-zone building exterior. Simplified 3D model of the pilot cultivation chamber (2.40 × 3.00 × 2.50 m) used in TRNSYS simulations, with adjacent blocks representing shading elements.

Each cultivation cycle begins with seed germination in nursery trays containing cocopeat or perlite for 10 days under constant 50–70% relative humidity. Following transplanting, crops were grown under controlled conditions in a vertical rack system with recirculating hydroponics. Two substrates were evaluated: (i) mineral wool (rockwool), and (ii) expanded clay pellets. The rockwool system exhibited superior water-holding capacity and capillarity, supporting maximum yields of 5.26 kg/m ^2^ per cycle, compared to 3.82 kg/m ^2^ in the clay pellet system. The nutrient solution was based on a modified Hoagland formulation with continuous pH and EC monitoring. The pH was maintained at 5.5–6.0, while electrical conductivity ranged from 1.8 to 2.2 mS/cm, adjusted according to growth stage. The solution was recirculated at a flow rate of 1.2–1.5 L/min, ensuring full saturation and oxygenation. Water temperature was kept at 18–20°C to minimize thermal shock and root stress. No artificial CO _2_ enrichment was applied. Instead, air exchange was passively regulated to maintain ambient CO _2_ concentrations (~400–450 ppm). Relative humidity was controlled at 50–70%, with automated misting used during the seedling stage. The vertical racks consisted of 4 cultivation tiers spaced 50 cm apart, with LED luminaires delivering variable PPFD levels (200–350 µmol/m ^2^/s) depending on the lighting scenario. Harvest took place at physiological maturity (~120–150 g fresh weight per head), depending on lighting strategy and environmental control conditions. Vertical farming system is realized in ISOBOX with galvanized metal construction of 4 cm sandwich panel insulation, Aluminium door with LED lights of 1kW. The dimensions of the ISOBOX are 240cm x 300cm x 250cm (LxWxH) ( Figure 2) as shown in the sketch which will be introduced in TRNSYS 18 software. The system includes controlled-environment parameters, such as artificial lighting, HVACD (heating, ventilation, air conditioning, and dehumidification, in this case a heat-pump), circulation pumps and fans. All these components of the VF system are presented on the ( Table 1) in the Manuscript Tables section. The cultivation area comprised four vertical hydroponic layers capable of housing up to 200 lettuce heads per cycle, producing approximately 40–45 kg of lettuce per cultivation cycle.

This simplified 3D illustration represents the pilot unit used in the vertical farming study. Dimensions are provided in meters. The orange structure represents the cultivation chamber, while the purple volumes indicate adjacent buildings or shading elements. This schematic was created using SketchUp.

### 3.2 System setup

**Figure 3.**
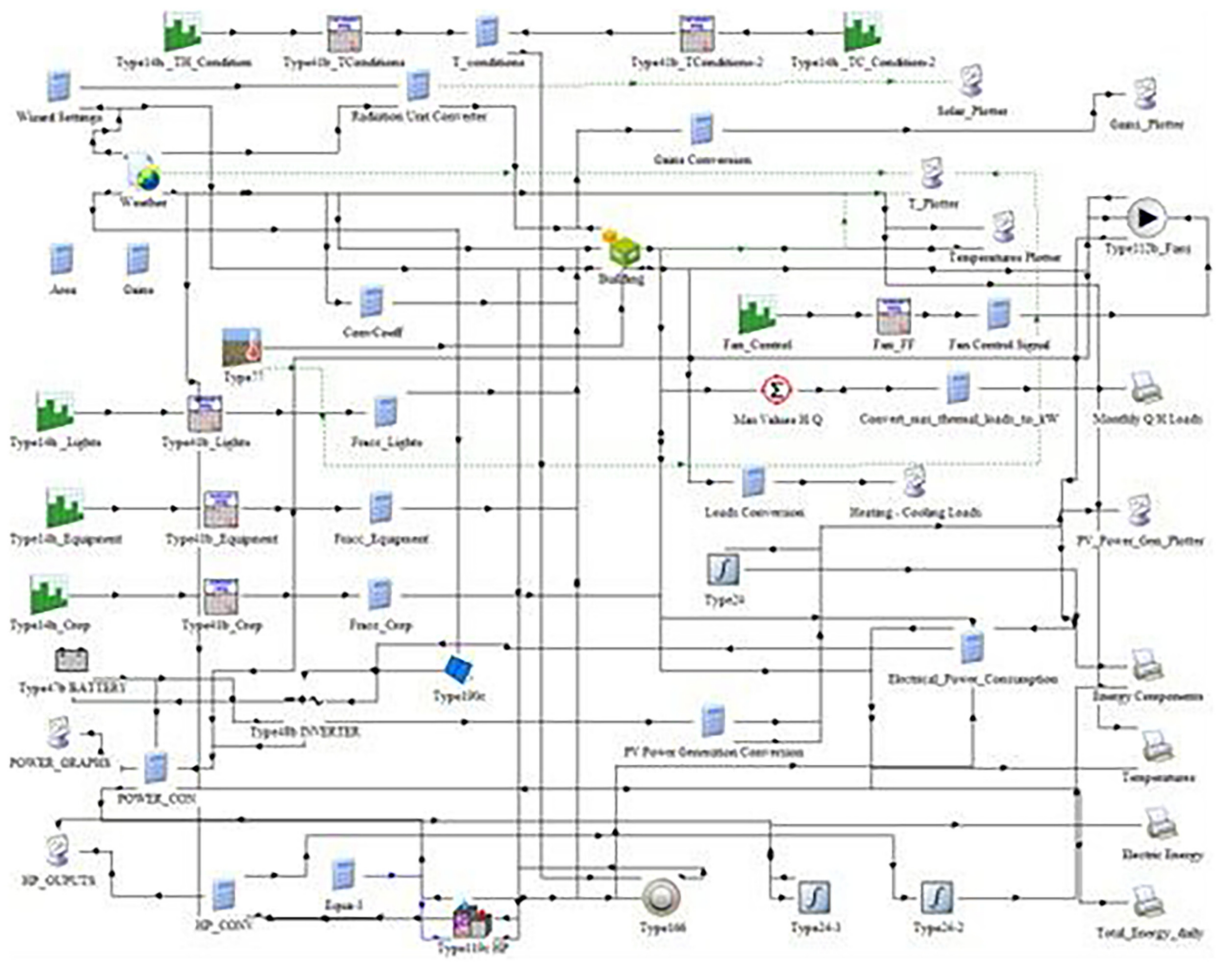
Configuration of the TRNSYS 18 model for the vertical farming unit. The schematic integrates weather inputs, lighting, HVAC, PV generation, battery storage.

The energy simulation was performed using TRNSYS 18, a dynamic simulation software widely used for analyzing energy systems ( Figure 3). Within this environment, a full-year simulation was developed to capture quarter hourly fluctuations in energy demand and supply for the pilot VF system. Simulations replicate the structural and operational characteristics of the existing hydroponic vertical farm system operating under the COALITION project. The HVACD system was modeled, along with climate database taking from METEONORM for the case of Northern Greece.

This schematic illustrates the structure of the simulation model, including components for weather input, lighting, HVAC, photovoltaic power generation, battery storage, and crop energy demand. The model enables the calculation of total energy consumption and PV contribution under different cultivation scenarios. This flowchart was exported from TRNSYS 18 Studio.

All the energy components of the VF system, including lighting, heating, cooling, and dehumidification, were simulated in an integrated manner within TRNSYS 18 to assess seasonal performance. PV generation was also modeled matching the actual specifications of the installed VF system, which is integrated with inverter and batteries. The energy systems were incorporated to simulate load shifting and storage of surplus PV energy production, while the MPPT inverter regulates energy from PV to storage, to consumption for the components that require energy and to the energy grid for surplus energy production. Thermal energy gains of the VF system derive from crop cultivation of lettuce in terms of latent heat throughout their evapotranspiration. Latent heat gains resulting from plant evapotranspiration were estimated by quantifying the mass of water transpired by lettuce plants and applying the latent heat of vaporization. The process accounts for the energy required to convert liquid water released by the plants into water vapor, which contributes to the internal latent load of the space. The total latent heat gain (Q _latent_) was calculated using the equation:


$$Q_{latent}=m\cdot h_{fg}\;(1)$$

where *m* is the mass flow rate of transpired water (kg/day or kg/h), and h _*fg*_ is the latent heat of vaporization of water which is approximately 2,450 kJ/kg, at 20 °C under standard atmospheric pressure. Transpiration rate was derived from literature values specific to indoor vertical farming conditions for lettuce, taking into account light intensity, temperature, and relative humidity. A value in the range of 0.2-0.5 l/day per plant was used to reflect typical water loss through transpiration under controlled environment agriculture (CEA) ( 22, 23). Sensible heat gains concern the electrical equipment of the VF system including fans, pumps, and the lighting system. The model incorporates dynamic interactions between internal loads (e.g. lighting, plant evapotranspiration, electrical equipment, etc.), external climate conditions, and HVACD system. Indoor setpoints for each scenario (heating, cooling, humidity, lighting duration) were introduced as control parameters, dynamically adjusted to reflect realistic operating logic for lettuce cultivation. Humidification and dehumidification were accounted for by balancing vapor pressures based on the indoor air setpoints and crop transpiration rates.

### 3.3 Scenario definition and crop cycles

To explore the dynamic behavior of energy consumption in vertical farming, a number of cultivation scenarios were conducted, each representing a specific combination of environmental conditions relevant to lettuce growth in terms of temperature set-point and humidity levels. The indoor environmental conditions were tailored for safe lettuce growth. Lighting requirements were defined across various scenarios, reflecting the photoperiods. Thus, three key environmental parameters were systematically varied across scenarios ( Table 3):

- Relative humidity, ranging from 50% to 70%,
- Photoperiod duration, from 10 to 16 hours per day, and
- Setpoint temperatures, with heating temperatures from 12°C - 18°C and cooling thresholds from 20°C - 28°C.

**Table 3.** Multiple Linear Regression Model.

ANOVA Table (Crop Cycle ~ ΔT + Photoperiod + Humidity)					
Source	DF	Sum of Squares	Mean Square	F-value	P-value
**ΔT**	1	533.94	533.94	3879.33	<0.0001
**Photoperiod**	1	540	540	3923.33	<0.0001
**Humidity**	1	0	0	0	0.998
**Residual**	44	6.06	0.14		
Model Summary					
Statistic	Value				
R-squared (R²)	0.994				
Adjusted R-squared	0.994				
F-statistic	2601				
Prob (F-statistic)	1.59E-49				
Observations	48				
Degrees of Freedom	44 (residual)				
Coefficient Table					
Term	Coefficient	Std. Error	t-value	P-value	Interpretation
Intercept	57.02	0.512	111.3	<0.001	Base duration with zero inputs
ΔT	0.645	0.01	62.3	<0.001	Longer cycles with wider temperature gap
Photoperiod	–1.5	0.024	–62.6	<0.001	Shorter cycles with longer light periods
Humidity	~0.0001	0.007	≈ 0	0.998	No significant effect on crop duration

Each one of the scenarios represents a distinct cultivation schedule, with unique crop cycle durations and annual repetition frequencies. In order to find the required crop cycle for each scenario values were taken from literature overview as presented in Table 2 concerning the lettuce cultivation conditions as well as data taken from the operation of the installed VF system. Thus, a multiple linear regression model was developed to estimate crop cycle duration as a function of environmental control parameters. Using 48 scenario configurations, the model considered temperature differential (Δ *T*), photoperiod, and humidity as predictive variables.


$$\text{y}={\text{β}}_{0}+{\text{β}}_{1}{\text{x}}_{1}+{\text{β}}_{2}{\text{x}}_{2}+\text{⋅}\cdot \cdot .+{\text{β}}_{\text{p}}{\text{x}}_{\text{p}}+\text{ε}\;(2)$$

Where:

- y: Dependent variable (response)
- β _0_ : Intercept (value of y when all x _i_ = 0)
- β _1,_ β _2_,……,β _p_ : Regression coefficients
- x _1_, x _2_..., xp: Independent variables (predictors)
- ε: Error term (residual)

Thus, the final fitted model used in all scenario computations was:


CropCycle(days)=57.02+0.645⋅ΔT–1.50⋅Photoperiod+0.0001⋅Humidity(3)

with R ^2^ = 0.994, Adj-R ^2^ = 0.994, and the humidity term non-significant (p ≈ 0.998).

**Figure 4.**
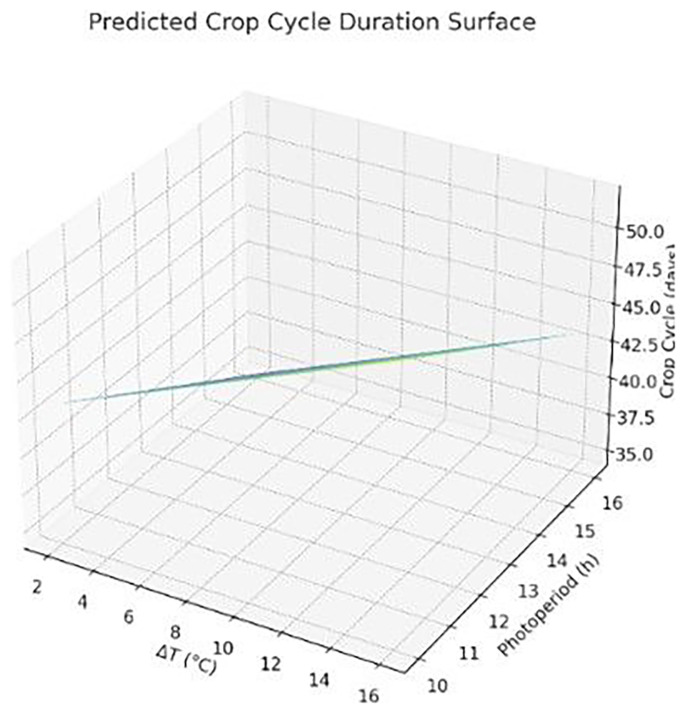
Predicted crop cycle duration as a function of photoperiod and temperature difference (ΔT). Longer light periods reduce cycle length, while wider ΔT slightly increases it.

The following Figure ( Figure 4) presents a 3D surface plot visualizing the Final Crop Cycle Estimation Model, which shows that crop cycles decrease significantly with increasing photoperiods, while they increase modestly with higher ΔT (temperature difference). This confirms the dominant role of light duration in accelerating growth, with ΔT offering a secondary, supportive influence.

The 3D surface represents the modeled crop cycle duration (in days) as a function of photoperiod (h/day) and temperature difference (ΔT in °C). Results were derived using a multiple linear regression model applied to simulation outputs. Graph produced using AI-assisted plotting tools.

Thus, the scenarios simulate a distinct cultivation strategy that leads to a different crop cycle duration, varying between 34 and 52 days as presented in Table 4. Shorter cycles (34–37 days) were observed in configurations combining high photoperiods (16 h), moderate humidity (50–60%), and elevated temperatures (18°C heating, 20°C cooling). Conversely, longer cycles (49–52 days) appeared in scenarios with lower light exposure (10 h), minimal heating (12°C), and broader temperature differentials between day and night. This structured variation allows for assessing how tighter climate control and intensified lighting can accelerate plant development—thereby increasing the number of annual cycles—while also evaluating the trade-offs in energy demand and sustainability. TRNSYS 18 simulations produced energy demand profiles for lighting and HVACD, which were subsequently compared against simulated PV energy availability on a quarter hourly basis. The aim was to quantify PV sufficiency across all scenarios and to assess energy demands.

**Table 4.** Environmental parameter combinations across the 48 lettuce cultivation scenarios, with estimated crop cycle durations.

Scenario	Heating Temperature (°C)	Cooling Temperature (°C)	Humidity (%)	Photoperiod (hours)	Estimated Crop Cycle (days)
s1	12	28	50	10	52
s2	12	28	50	12	49
s3	12	28	50	14	46
s4	12	28	50	16	43
s5	12	28	60	10	52
s6	12	28	60	12	49
s7	12	28	60	14	46
s8	12	28	60	16	43
s9	12	28	70	10	52
s10	12	28	70	12	49
s11	12	28	70	14	46
s12	12	28	70	16	43
s13	14	24	50	10	49
s14	14	24	50	12	46
s15	14	24	50	14	43
s16	14	24	50	16	40
s17	14	24	60	10	49
s18	14	24	60	12	46
s19	14	24	60	14	43
s20	14	24	60	16	40
s21	14	24	70	10	49
s22	14	24	70	12	46
s23	14	24	70	14	43
s24	14	24	70	16	40
s25	16	22	50	10	46
s26	16	22	50	12	43
s27	16	22	50	14	40
s28	16	22	50	16	37
s29	16	22	60	10	46
s30	16	22	60	12	43
s31	16	22	60	14	40
s32	16	22	60	16	37
s33	16	22	70	10	46
s34	16	22	70	12	43
s35	16	22	70	14	40
s36	16	22	70	16	37
s37	18	20	50	10	43
s38	18	20	50	12	40
s39	18	20	50	14	37
s40	18	20	50	16	34
s41	18	20	60	10	43
s42	18	20	60	12	40
s43	18	20	60	14	37
s44	18	20	60	16	34
s45	18	20	70	10	43
s46	18	20	70	12	40
s47	18	20	70	14	37
s48	18	20	70	16	34

### 3.4 Simulation process

**Figure 5.**
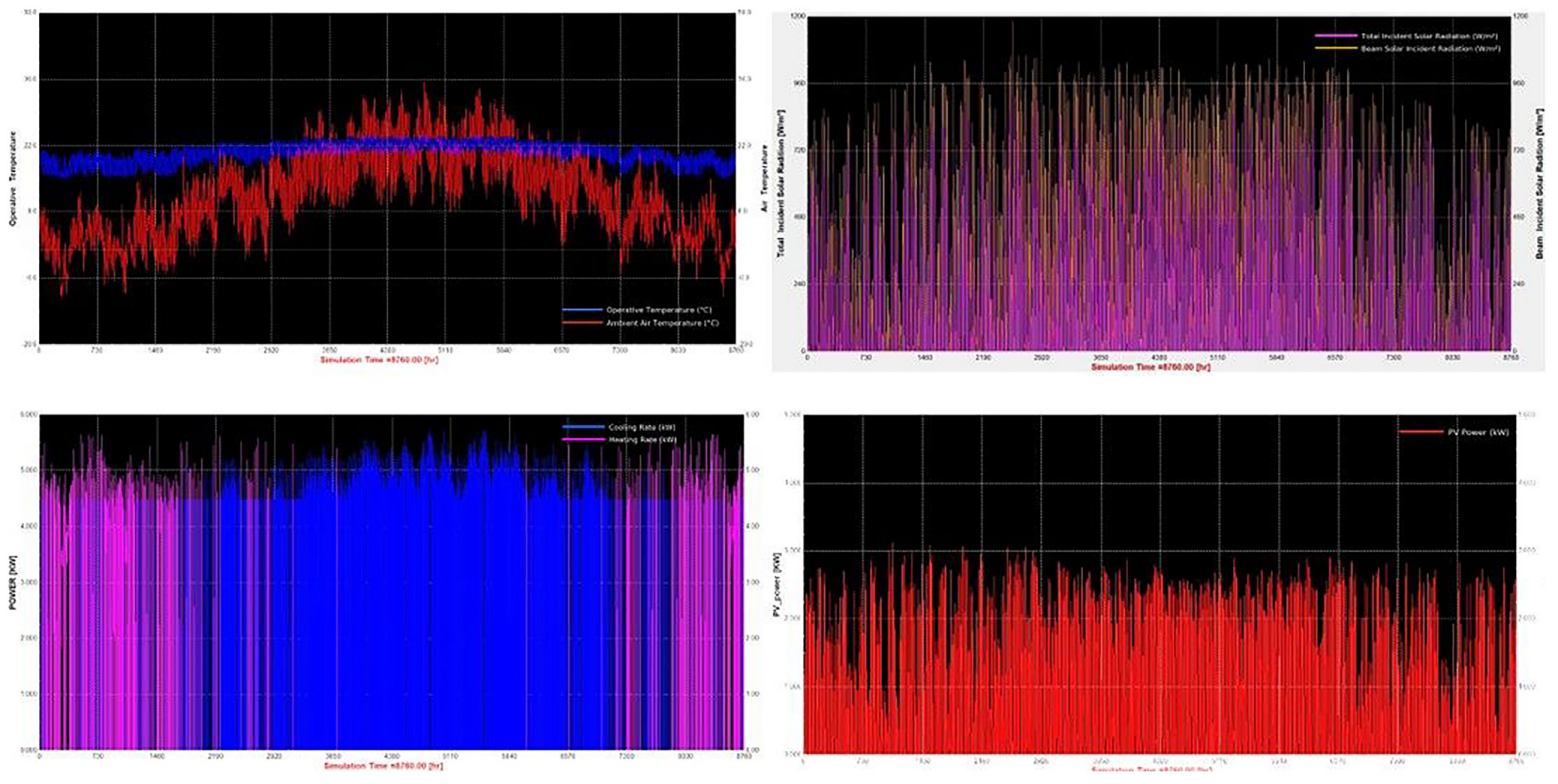
TRNSYS 18 Simulation Results for Specific Scenario.

The simulation process employed in this study was designed to capture the intricate energy dynamics of a fully enclosed VF system operating under the control scenarios discussed in Section 3.3. The core simulations via TRNSYS 18, for quarter-hourly data were generated for a full year, capturing the real-time interactions among lighting, HVACD systems, photovoltaic (PV) energy input, and battery storage behavior. For each one of the scenarios a set of annual data were produced including the following parameters: a) operating temperature and ambient temperature, b) total incident solar radiation and beam incident solar radiation, c) heat pump heating and cooling loads, d) PV power generation, e) energy required from grid and provided to grid, f) battery load cycles, g) energy consumption from electrical equipment. Figure 5 displays exported diagrams that were generated via TRNSYS 18 for a specific scenario ( Figure 5), where the top left figure shows the external and internal air temperatures over the simulation period, the top right displays the solar radiation and lighting power intensity, the bottom left shows the cooling (blue) and heating loads (pink) and lastly the bottom right displays the PV power generation profile.

TRNSYS 18 produced a number of diagrams that illustrate the dynamic behaviour of environmental and energy parameters throughout the year for a representative scenario (e.g., Scenario S40), which combines an intensive photoperiod (16 hours), strict temperature control (18 °C heating/20 °C cooling), and moderate humidity (60%). This configuration refers to a minimized crop cycle duration (34 days), thereby maximizing annual production cycles. For instance, Indoor vs. Ambient Temperature compares the ambient outdoor temperature (red) with the tightly regulated indoor temperature (blue). The indoor environment is maintained consistently approximately between 18 °C to 22 °C, regardless of external fluctuations, demonstrating the efficiency and responsiveness of the HVAC system in providing stable thermal conditions for optimal lettuce growth. The Incident Solar Radiation figure depicts total (magenta) and beam (yellow) solar radiation incident on the surface. As expected, solar input varies seasonally, peaking during spring and summer. However, frequent fluctuations and low winter irradiance confirm the system’s dependence on grid energy during low-sunlight periods where PV is not performing at its nominal capacity. The Figure concerning the PV Energy Consumption indicates continuous operation throughout the year, aligned as is expected for the conditions of Northern Greece. Variations reflect daily energy production cycles, where the energy production decreased significantly during winter months, highlighting the dominant dependence from grid energy consumption. Whereas, the heat pump's power input throughout the year shows consumption peaks in winter due to heating demands and rises again in summer due to cooling energy requirements. This pattern closely follows the inverse of outdoor temperature trends, confirming the seasonal nature of HVAC loads. All these data depicted on the plots above demonstrate how intensive environmental control strategies significantly increase energy demands – which vary for each scenario - but offer the benefit of reduced cultivation time. The strong interplay between lighting schedules, external climate, and HVAC loads emphasizes the importance of integrated energy planning. These temporal profiles form the basis for further analyses presented in the next sections, including PV sufficiency assessment, dynamic lighting evaluation, and scenario-based carbon footprint comparisons.

### 3.4. CO _2_ emissions estimation

For each scenario (S1–S48), monthly simulation data were aggregated to derive the annual energy requirements of the system. Grid electricity consumption was isolated from the total demand using energy flow logs, ensuring that only non-renewable coverage was considered in the carbon footprint calculation. Emission factor for electricity was drawn from the Greek National Inventory, with average value of 0.256 kg CO _2_/kWh applied to grid-supplied electricity, corresponding to Greece’s average energy mix ^24^. No feed-in to the grid was assumed, as the system operates in a self-consumption model with surplus energy curtailed or stored locally. Based on these an LCA for greenhouse gas emissions (GWP) was conducted per kilogram of harvested lettuce for each scenario of the crop cycles. Components for this analysis, apart from electricity energy for LED lighting, also include the water pumping system which reflects the continuous energy demand of closed-loop recirculation systems. Other minor CO _2_ emission contributions arise from valves and monitoring/sensor systems. The life cycle assessment (LCA) for greenhouse gas emissions (GWP) was conducted using system boundaries encompassing all material and energy inputs associated with a full cultivation cycle of the vertical farming unit. This included substrates (rockwool and clay pellets), growth media, total water consumption, and hybrid electricity supply from both PV and grid sources. The LCI inventory was set up based on real-time monitoring data from the pilot unit (e.g., water use, grid electricity), complemented with literature-based assumptions on germination duration. The functional unit was defined as 1 kg of harvested fresh lettuce (average head weight 250 g). A detailed breakdown of total electricity uses and corresponding CO _2_ emissions per scenario is presented in Table 5. Other components of CO _2_ emissions that were included in LCA refer to the cultivation of lettuce such as rockwool and clay pellets. Although, neither substrate significantly affected the carbon footprint, this variation suggests potential environmental optimization opportunities depending on the choice of substrate in large-scale applications. It is important to note, however, that this comparison assumes equal production efficiency between substrates, without accounting for the compatibility between crop and media. In practice, rockwool offers superior water retention, aeration and nutrient delivery, making it more suitable for lettuce cultivation. Conversely, clay pellets, though reusable and more sustainable in lifecycle terms, may require more precise irrigation and nutrient control to achieve comparable yields. Thus, substrate selection should consider both environmental performance and operational efficiency.

**Table 5.** CO2 Emissions for each scenario based on LCA.

CO2 Emissions for Each Scenario based on LCA					
Scenarios	Total Electricity (kWh)	Total CO2 (kgCO2eq/kg lettuce)	Scenarios	Total Electricity (kWh)	Total CO2 (kgCO2eq/kg lettuce)
S1	2455.3	2.3	S25	3839.2	3.5
S2	3198.1	2.9	S26	4587.9	3.6
S3	3908.7	3.5	S27	5242.2	3.7
S4	4659.2	3.7	S28	5264.5	3.7
S5	2460.8	2.3	S29	3858.3	3.5
S6	3210.9	2.9	S30	4607.8	3.7
S7	3921.5	3.6	S31	5262.3	3.7
S8	4674.4	3.7	S32	5980.3	4.2
S9	2472.0	2.3	S33	3876.8	3.5
S10	3217.3	2.9	S34	4614.0	3.7
S11	3940.4	3.6	S35	5280.3	3.7
S12	4693.6	3.7	S36	6002.2	4.2
S13	3068.3	2.8	S37	4971.5	3.9
S14	3808.0	3.5	S38	5712.7	4.0
S15	4486.4	3.6	S39	6346.8	4.4
S16	5216.8	3.7	S40	7061.0	4.4
S17	3082.7	2.8	S41	4989.3	3.9
S18	3816.3	3.5	S42	5722.6	4.0
S19	4504.1	3.6	S43	6363.5	4.5
S20	5242.2	3.7	S44	7086.3	4.5
S21	3093.6	2.8	S45	5015.2	4.0
S22	3829.3	3.5	S46	5740.7	4.0
S23	4523.1	3.6	S47	6383.9	4.5
S24	5264.5	3.7	S48	7092.5	4.5

While PV generation offsets a substantial portion of grid reliance, the prevailing fossil-heavy Greek energy mix and continuous lighting demands drive emissions upward. These findings highlight both the opportunity and challenge in decarbonizing vertical farming: while integrating on-site renewables like PV significantly lowers emissions, design optimization (e.g., dynamic lighting, shorter cycles) remains crucial for further reducing the carbon footprint per kilogram of product produced.

This approach underlines the critical role of renewable integration and system design in minimizing environmental impacts of vertical farming.

## 4. Results and discussion

### 4.1. Annual energy demand & grid dependency per scenario

**Figure 6.**
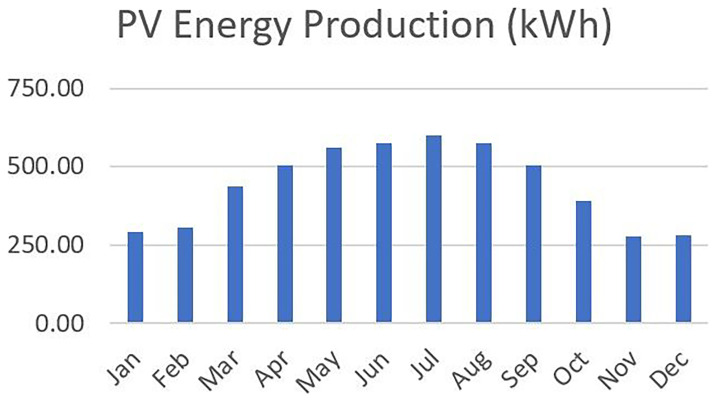
Monthly PV energy production profile for Northern Greece, with higher output in summer and lower in winter.

***4.1.1 Annual PV energy production & energy analysis.*** The PV integrated into the VF unit was modeled under fixed operational parameters based on the optimal tilt angle and orientation, for the case of Northern Greece. The system’s specifications remained the same across all 48 scenarios. Based on local solar irradiance data and TRNSYS 18 simulations, the annual electricity generation from the PV system was estimated at approximately 5,234 kWh, with monthly values ranging from 280 kWh in December to 600 kWh in July, following the typical seasonal pattern. This consistent energy profile served as the renewable input baseline across all scenarios. Any deviations in grid dependency, energy autonomy, or carbon footprint thus stem from the variation in environmental control settings (e.g., photoperiod, HVAC setpoints) rather than PV availability. Figure 6 illustrates the monthly distribution of PV energy production.

**Figure 7.**
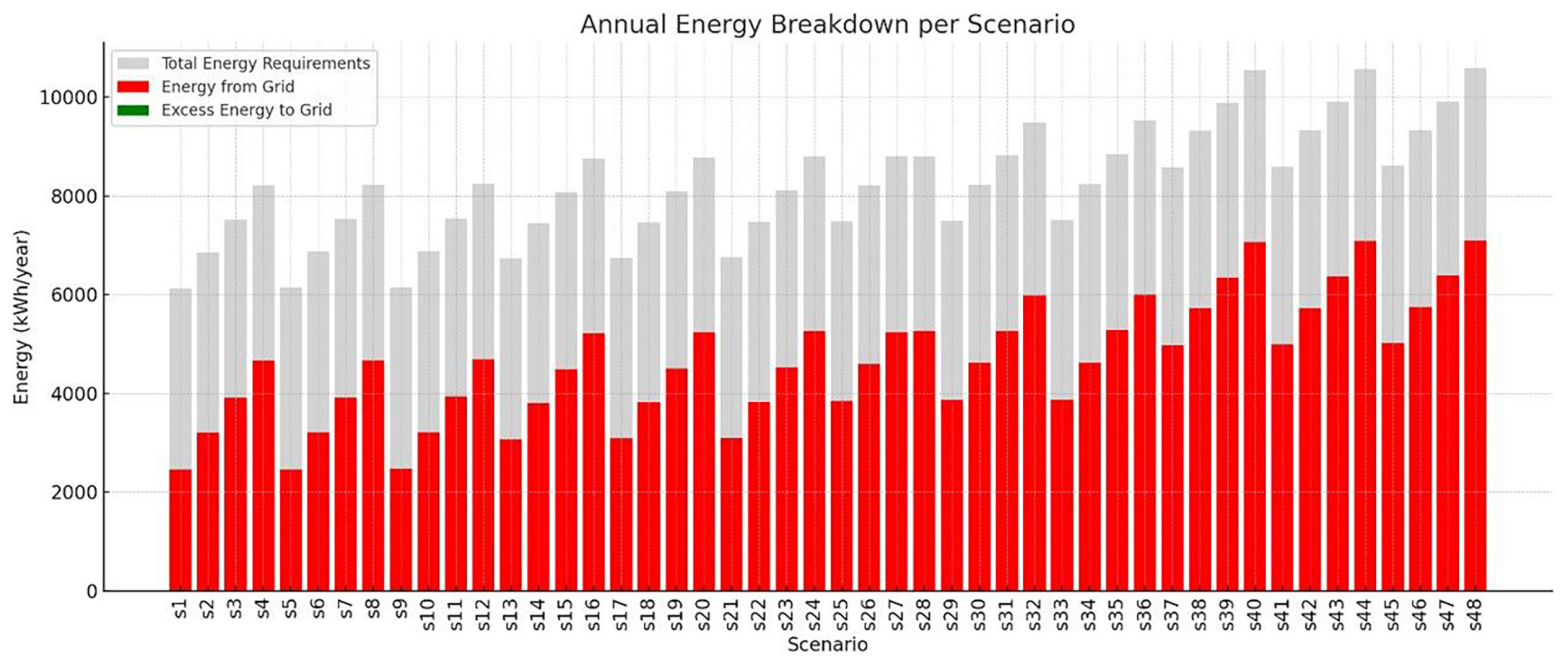
Breakdown of VF systems energy in annual basis.

The total annual energy requirements of the VF system vary considerably across the different scenarios, ranging from approximately 6,100 kWh to over 10,500 kWh ( Figure 7). This variability reflects differences in environmental control strategies—such as photoperiod, HVAC setpoints, and humidity levels—which directly impact the system’s lighting and climate energy loads. The amount of energy required from the grid also shows substantial fluctuation, spanning from almost 2,500 kWh in the most efficient scenarios (e.g., S1 and S5) to nearly 7,100 kWh in the most demanding cases. These figures underscore the importance of scenario configuration in determining energy autonomy. Scenarios with optimized settings—such as moderate temperature differentials and daylight-aligned photoperiods—demonstrate a significantly lower dependency on external energy sources. While the PV system contributes to reducing this grid reliance, the excess energy exported back to the grid remains relatively minimal in all cases, typically under 1% of total generation. For example, in scenarios like S1 and S5, although a small surplus of around 20 kWh is sent back to the grid, the majority of PV energy is consumed internally by the system (energy requirements and batteries). This indicates that the system operates close to a self-consumption model, with high internal utilization of PV electricity and limited overproduction. Overall, this energy breakdown confirms that while PV integration significantly offsets the total energy demand, grid dependency is still substantial in high-demand scenarios. The data also reinforces the value of seasonal crop scheduling, dynamic lighting control, and HVAC optimization to reduce total energy use and enhance self-sufficiency. In systems where excess PV is minimal, energy storage and load-shifting strategies become essential for further reducing grid reliance and improving energy resilience.

Each stacked bar represents the total annual energy demand (gray), the portion covered by grid electricity (red), and any excess PV energy fed back into the grid (green), across 48 simulation scenarios. Diagram generated using Python-based visualization tools and refined using AI-assisted layout optimization.

**Figure 8.**
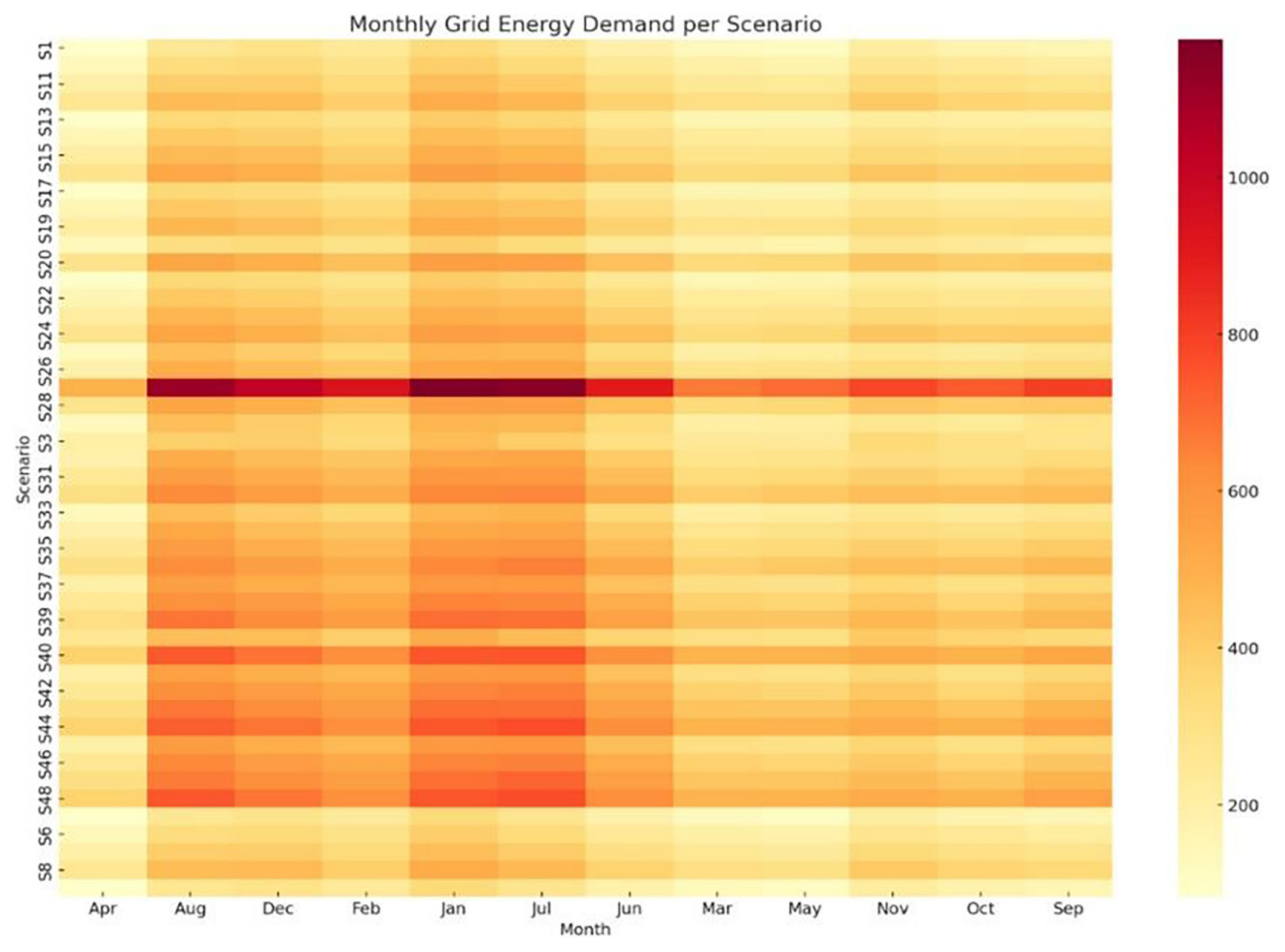
Monthly grid energy demand per scenario. Darker tones indicate higher dependency, with peaks in August, December, and January.

***4.1.2 Analysis of monthly grid energy demand by scenario.*** While electricity generation from PV remains fixed across all scenarios, the monthly grid energy demand varies significantly depending on the crop cycle configuration. These differences stem from diverse environmental control strategies, including heating and cooling setpoints, lighting durations, and humidity levels — all of which affect the total energy load and its seasonal distribution. To capture this variability, Figure 8 presents a heatmap of monthly grid electricity use for each scenario. The x-axis spans the calendar months, while the y-axis lists the 48 crop cycle scenarios sorted approximately by increasing annual energy consumption. This visualization reveals several key insights. Scenarios with higher energy demand exhibit intense grid dependency during months with large temperature fluctuations such as August, December and January reflected as deeper red tones on the heatmap. In contrast, energy-efficient scenarios, such as those with moderate environmental setpoints and shorter crop durations (e.g., S1–S10), display lighter tones, indicating lower grid dependency across the year. A notable asymmetry emerges in summer, where PV production peaks but energy demand may still rise sharply due to active cooling demands. This seasonal interplay underscores the importance of crop scheduling and environmental strategy optimization, particularly in months with low solar availability. Scenarios that compress crop cycles into spring and autumn months tend to benefit more from direct PV utilization.

Heatmap showing the variation in monthly grid energy requirements (in kWh) across all 48 simulation scenarios (S1–S48). Darker shades indicate higher dependency on grid electricity. Visualization generated using Python (matplotlib/seaborn); layout enhanced using AI-assisted tools.

**Figure 9.**
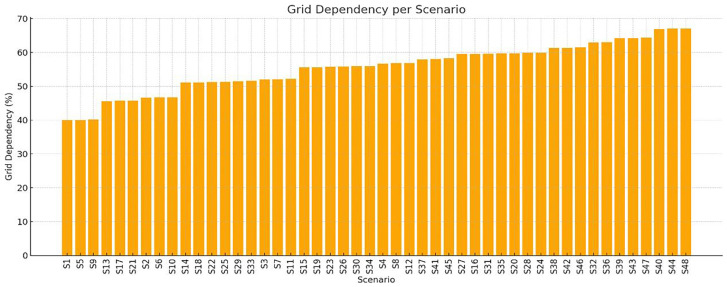
Grid dependency ratio for all 48 scenarios. Lower values (e.g., S1, S5, S9, S13) indicate higher PV contribution, while intensive scenarios (e.g., S44–S48) indicate higher grid reliance.

***4.1.3 Grid dependency per scenario.*** To better quantify the extent to which each scenario depends on grid for electricity supply, the Grid Dependency Ratio was computed for all 48 crop cycles. This metric reflects the percentage of the total annual energy demand that is met by the national grid, after accounting for the fixed annual PV contribution. Nearly all scenarios exhibit very high PV utilization (>99.6%), meaning almost all solar energy is consumed by the system. Since PV production is fixed for all scenarios, efficiency hinges on reducing demand or improving storage/load matching. Thus, the total energy demand varied significantly as a result of changing photoperiods, HVAC setpoints, and humidity levels. This variation in demand causes the share of PV energy to fluctuate accordingly, impacting grid reliance. The bar chart in Figure 9 illustrates the Grid Dependency Ratio values per scenario. Scenarios such as S1, S5, S9, and S13 exhibit the lowest grid dependency (~40%), corresponding to milder temperature settings, shorter photoperiods, and moderate humidity levels. On the contrary, scenarios like S44, S47, and S48 demonstrate the highest dependency, exceeding 66%, primarily due to extended lighting durations and elevated heating setpoints. This visualization underscores the direct relationship between crop cycle intensity and grid reliance. Scenarios that aim for smaller crop cycles typically impose higher energy burdens -particularly in lighting and heat-pump use- thus diluting the relative impact of PV production. Conversely, scenarios with larger crop cycles align better with the PV output, requiring less energy from the grid.

Bar chart illustrating the percentage of grid dependency for each of the 48 simulation scenarios (S1–S48), based on annual energy balance results. Higher values reflect increased reliance on external electricity sources and reduced energy autonomy. Plot generated using Python's matplotlib; layout support with AI-assisted enhancement.

### 4.2 Resilience analysis

**Figure 10.**
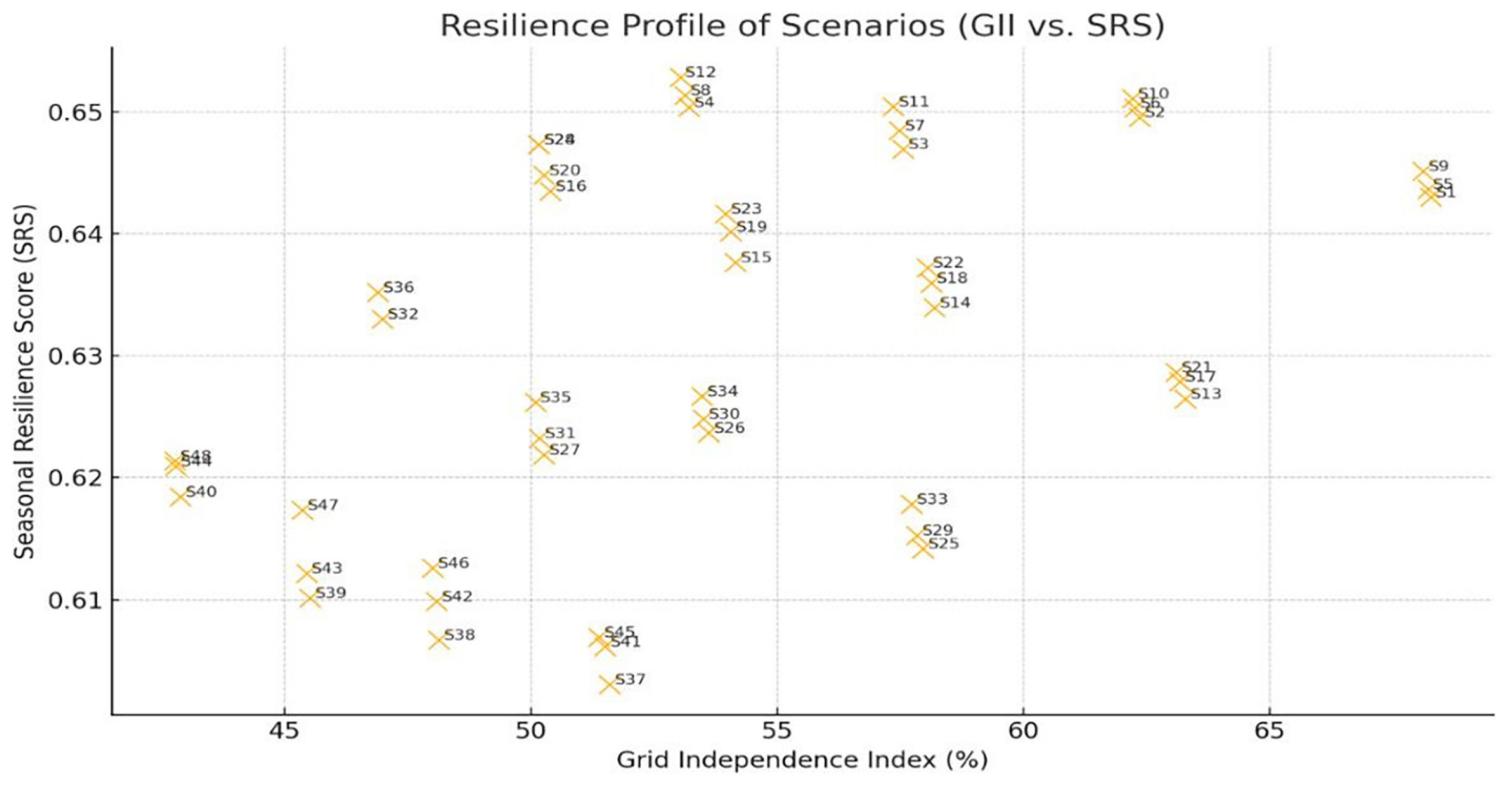
Resilience profile of scenarios based on GII and SRS. Upper-right cases are more resilient, while lower-left are grid-dependent.


To assess the adaptability of each vertical farming scenario under fluctuating solar and thermal conditions, we evaluated their resilience profiles using two metrics: the Grid Independence Index (GII) and the Seasonal Resilience Score (SRS). GII captures the proportion of energy self-supplied by PV and batteries, while the SRS reflects a scenario’s ability to maintain PV sufficiency during its worst-performing month compared to average months. These indicators revealed distinct clusters of behavior across all scenarios ( Figure 10).

$$\text{GII}=\left(1-\frac{\text{Annual}\;\mathrm{Grid}\;\text{Energy}\;\text{Use}}{\text{Total}\;\text{Annual}\;\text{Energy}\;\text{Demand}}\right)\times 100\;(4)$$

$$\text{SRS}=\frac{\text{PV}\;\text{Coverage}\;\text{In}\;\text{Lowest}\;\text{Month}}{\text{Average}\;\text{Monthly}\;\text{PV}\;\text{Coverage}}\;(5)$$

$$\text{PV}\;\text{Coverage}=\frac{\text{PV}\;\text{Used}}{\text{Monthly}\;\text{Energy}\;\text{Demand}}\;(6)$$

Scenarios such as S22 and S11 exhibited both high GII and SRS, suggesting a strong capacity to operate independently from the grid while maintaining consistency throughout seasonal fluctuations. For instance, S1 achieved the highest Grid Independence Index (GII) at 68.3%, meaning that nearly two-thirds of its annual energy needs were met by PV without grid support. Its Seasonal Resilience Score (SRS) of 0.643 indicates that even in its worst-performing month, PV still covered approximately 64% as much demand as in an average month, showing moderate seasonal vulnerability. By contrast, scenarios like S12 showed lower GII (53.0%), pointing to higher dependence on grid energy, though with relatively stable seasonal PV performance (SRS ≈ 0.653) which is therefore excellent candidates for off-grid or hybrid renewable deployments. Conversely, scenarios falling into the low GII/low SRS quadrant (e.g., S40, S43) are more dependent on grid support and vulnerable during seasonal troughs in PV generation. These findings highlight the need to evaluate both static energy sufficiency and temporal robustness. A high GII without seasonal stability may mask operational risks during winter or periods with low solar radiation, while high SRS without GII could still imply persistent grid dependence. The joint GII–SRS framework thus offers a more nuanced resilience benchmark that can inform not only technology selection but also adaptive scheduling strategies and tariff-aligned planting windows in climate-sensitive agricultural deployments.

Scatter plot illustrating the trade-off between Grid Independence Index (GII) and Seasonal Resilience Score (SRS) across all 48 examined scenarios. Scenarios in the upper-right quadrant exhibit both PV strong coverage and consistent seasonal performance, making them ideal for year-round sustainable operation. Scenario labels help identify where each falls on the resilience spectrum. Visualization generated in Python with enhanced AI-assisted labeling for interpretability and comparative clarity.

To further assess the robustness of each scenario under energy-constrained conditions, the resilience score of all scenarios was evaluated based on two dimensions: the worst-case PV deficit across a full crop cycle and the seasonal variability in PV availability. Four descriptive energy-performance typologies were set:

- **High Demand, Optimizable:** Heavy energy use but good potential for savings via cycle shifts or dynamic scheduling.
- **High Demand, Rigid:** High loads with limited flexibility — likely candidates for backup systems or setpoint reduction.
- **Moderate, Responsive:** Mid-range energy demand with high responsiveness — efficient and adaptable setups.
- **Stable, Low-Demand:** Low energy needs and minimal gains from optimization — ideal for low-intervention farming.

**Figure 11.**
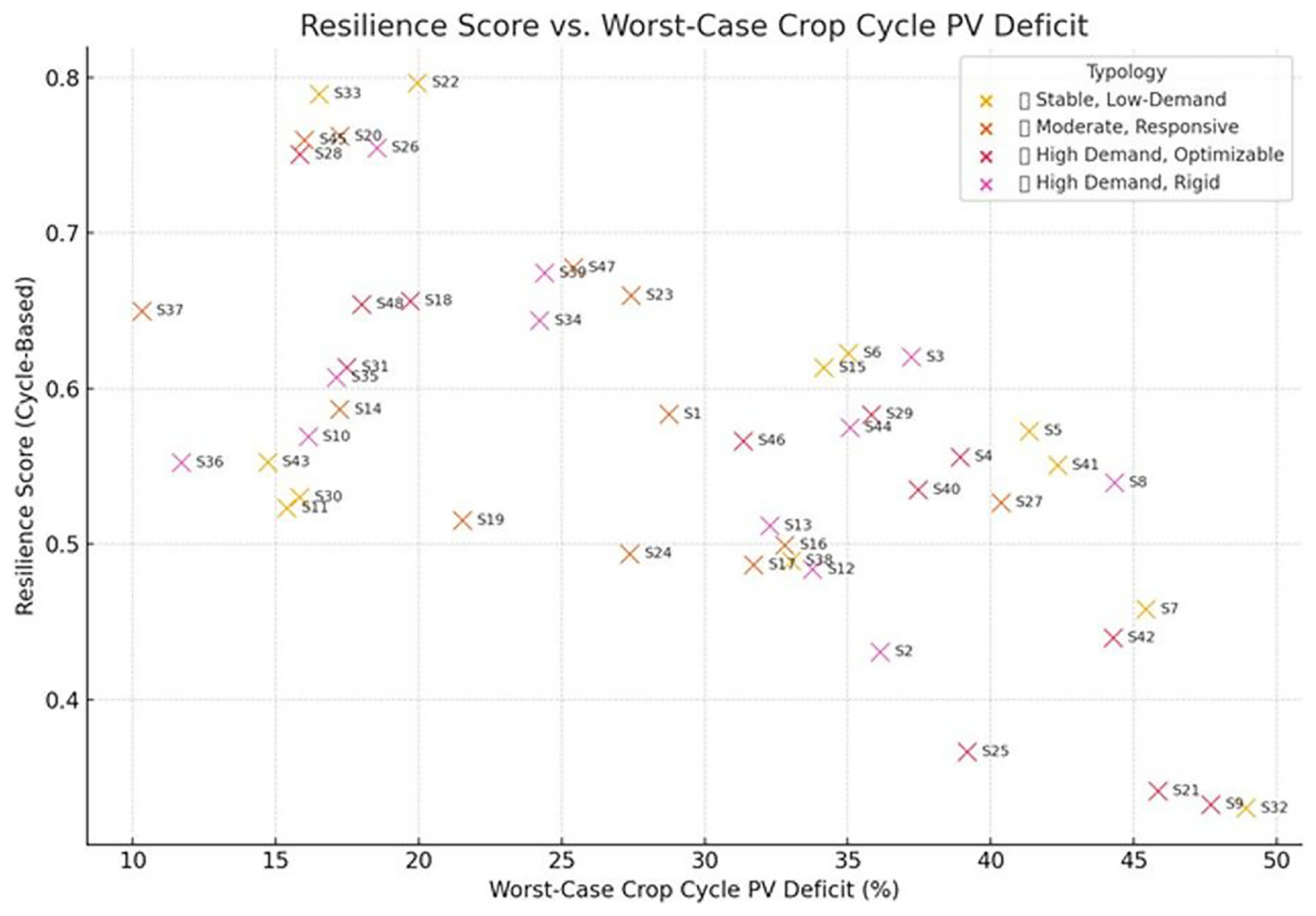
Resilience score versus worst-case PV deficit per crop cycle. Scenarios in the upper-right are more resilient, while those in the lower-left are vulnerable to energy shortages.

The resulting scatter plot revealed clear stratification in scenario performance ( Figure 11). Scenarios such as S22, S33, and S26 clustered in the top-left quadrant, indicating a combination of low vulnerability during energy-scarce crop cycles and strong seasonal PV alignment—a hallmark of high resilience. These scenarios were predominantly associated with the aforementioned typologies, underscoring the benefit of balanced lighting and HVAC control profiles.

Scatter plot illustrating the trade-off between grid independence and seasonal robustness for all 48 simulated scenarios. Scenarios positioned in the top-right quadrant demonstrate both high autonomy from the grid and strong seasonal resilience. Visualization developed using Python with AI-assisted plotting tools for label clarity and layout optimization.

Conversely, scenarios like S9, S21, and S32 exhibited both high PV deficits and low seasonal resilience, suggesting a poor fit for standalone or PV-heavy configurations without grid support or advanced energy storage. These findings reinforce the value of incorporating cycle-specific energy stress testing into scenario evaluation, as it provides more actionable insight than averages or static load assumptions. This crop-cycle-centered resilience view is particularly relevant for dynamic scheduling models and for selecting configurations suitable for deployment in regions with fluctuating solar availability. The cycle-based resilience analysis provides critical insights for the design and operational scheduling of VF systems. Scenarios demonstrating high resilience scores—such as S22, S33, and S26—not only minimize reliance on external grid power during vulnerable seasonal windows but also show greater alignment between crop energy demands and on-site PV generation. These scenarios are ideal candidates for battery-supplemented PV systems or off-grid setups, as they reduce the likelihood of unmet demand during the most energy-stressed periods. On the other hand, low-resilience scenarios may benefit from adaptive interventions, such as cycle shifting, dynamic lighting control, or HVAC load smoothing, particularly during cloud-prone months. Integrating this analysis into operational guidelines can help determine when to plant, what setpoints to apply, and how to adjust lighting schedules to optimize energy use without sacrificing crop yield. When the resilience indicators were integrated with energy cost and carbon emissions in a multi-objective composite score, several key scenarios (notably S33, S22, and S43) consistently ranked at the top. These scenarios represent optimal trade-offs across sustainability and robustness—achieving low cost, low emissions, and high cycle resilience simultaneously. This reinforces the idea that energy-efficient design does not have to compromise system adaptability or sustainability. In contrast, some scenarios with strong sustainability performance (e.g., low cost and emissions) fared poorly in resilience, revealing potential hidden vulnerabilities under real-world energy dynamics.

***4.2.1 Optimal crop cycle and season placement.*** To better understand the interplay between energy efficiency and crop scheduling, a focused analysis was conducted on the minimum annual energy requirements associated with the most favorable crop cycle per scenario ( Table 6). These values derive from dynamic simulations incorporating TRNSYS 18 data and seasonal variations.

**Table 6.** Lowest Energy Demand Crop Cycles per Scenario.

Scenario	First Day of Cycle	Last Day of Cycle
s1	291	342
s2	295	343
s3	297	342
s4	306	348
s5	291	342
s6	294	242
s7	294	339
s8	306	348
s9	292	343
s10	294	342
s11	294	339
s12	306	348
s13	72	120
s14	286	331
s15	289	331
s16	292	331
s17	72	120
s18	286	331
s19	289	331
s20	292	331
s21	72	120
s22	286	331
s23	289	331
s24	292	331
s25	286	331
s26	289	331
s27	291	330
s28	295	331
s29	286	331
s30	289	331
s31	291	330
s32	295	331
s33	286	331
s34	289	331
s35	292	331
s36	295	331
s37	288	330
s38	291	330
s39	294	330
s40	297	330
s41	288	330
s42	291	330
s43	294	330
s44	297	330
s45	288	330
s46	291	300
s47	294	330
s48	297	330

**Figure 12.**
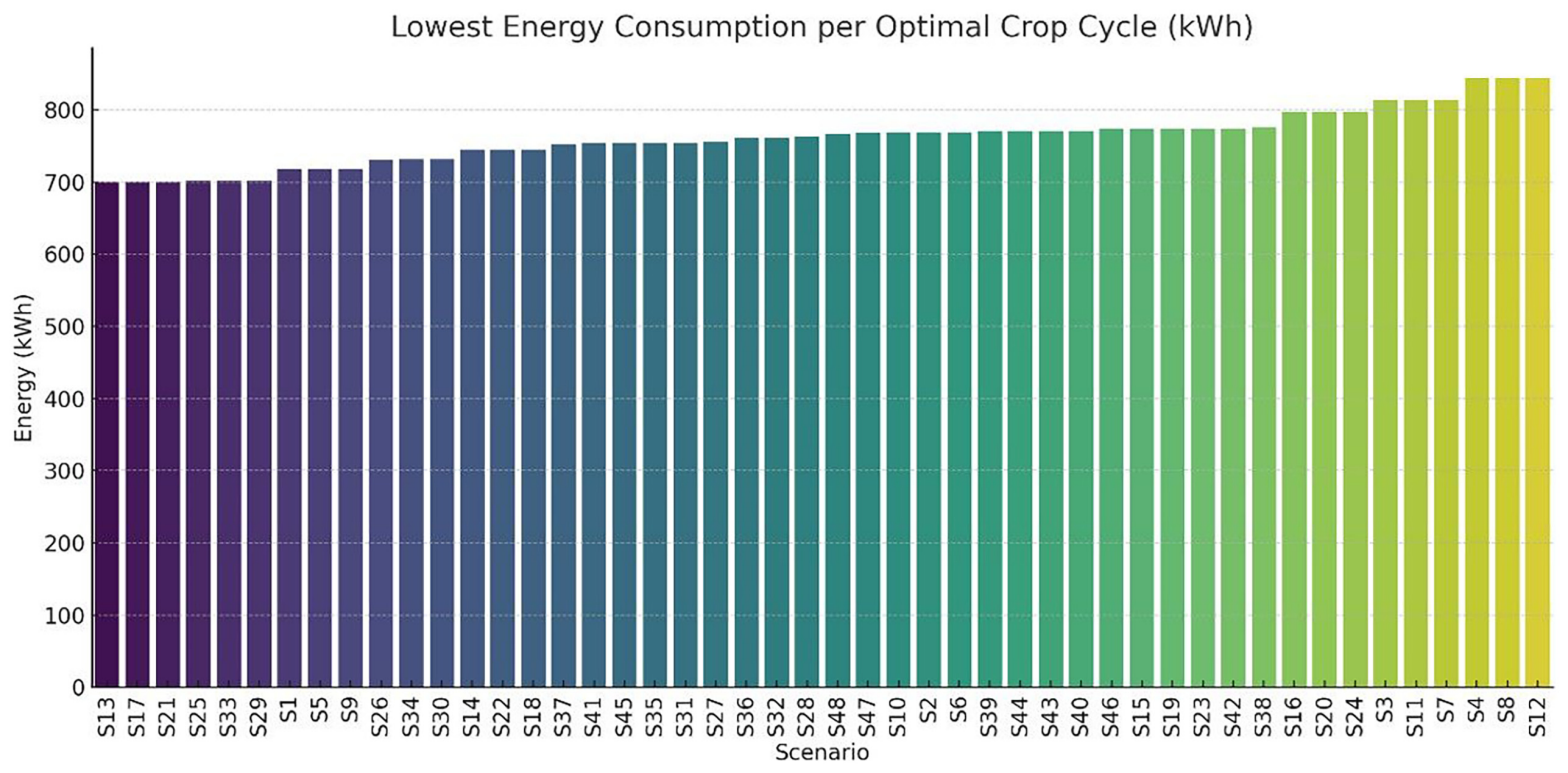
Optimal Crop Cycle Energy Profile example.

A typical depiction of s17 concerning the lowest energy demand for a full crop cycle, as well as its start and end dates during the year is presented in Figure 12. For this case the optimal crop cycle takes place in spring time.

The bar graph shows daily energy consumption (kWh) throughout the year, with the optimal crop cycle window indicated in green. This period was identified through simulation as the most energy-efficient and resilient interval for cultivation under Scenario 17 conditions. Diagram generated using AI-assisted plotting tools in Python for enhanced visual clarity *.*

**Figure 13.**
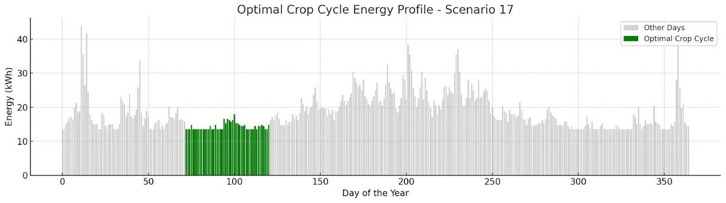
Optimal crop cycle energy demand across all scenarios. Lower values indicate more efficient environmental settings, while higher values reflect intensive control requirements.

A study was conducted on the optimal energy demand per scenario along with the corresponding first and last day of the crop cycle. The results are illustrated in Figure 13. Scenarios such as S1, S13, S17, and S21 exhibit the lowest energy consumption (approximately 700–720 kWh), while other scenarios (e.g., S4, S12, S8) surpass 840 kWh. These variations are primarily driven by setpoint configurations (e.g., heating/cooling set temperatures) and photoperiod duration.

The bar chart presents the total energy consumed (in kWh) during the most efficient cultivation cycle for each scenario (S1–S48). Scenarios are ranked from lowest to highest energy requirement, illustrating the variation in energy efficiency depending on environmental and operational parameters. Chart created using Python with AI-assisted data visualization techniques.

**Figure 14.**
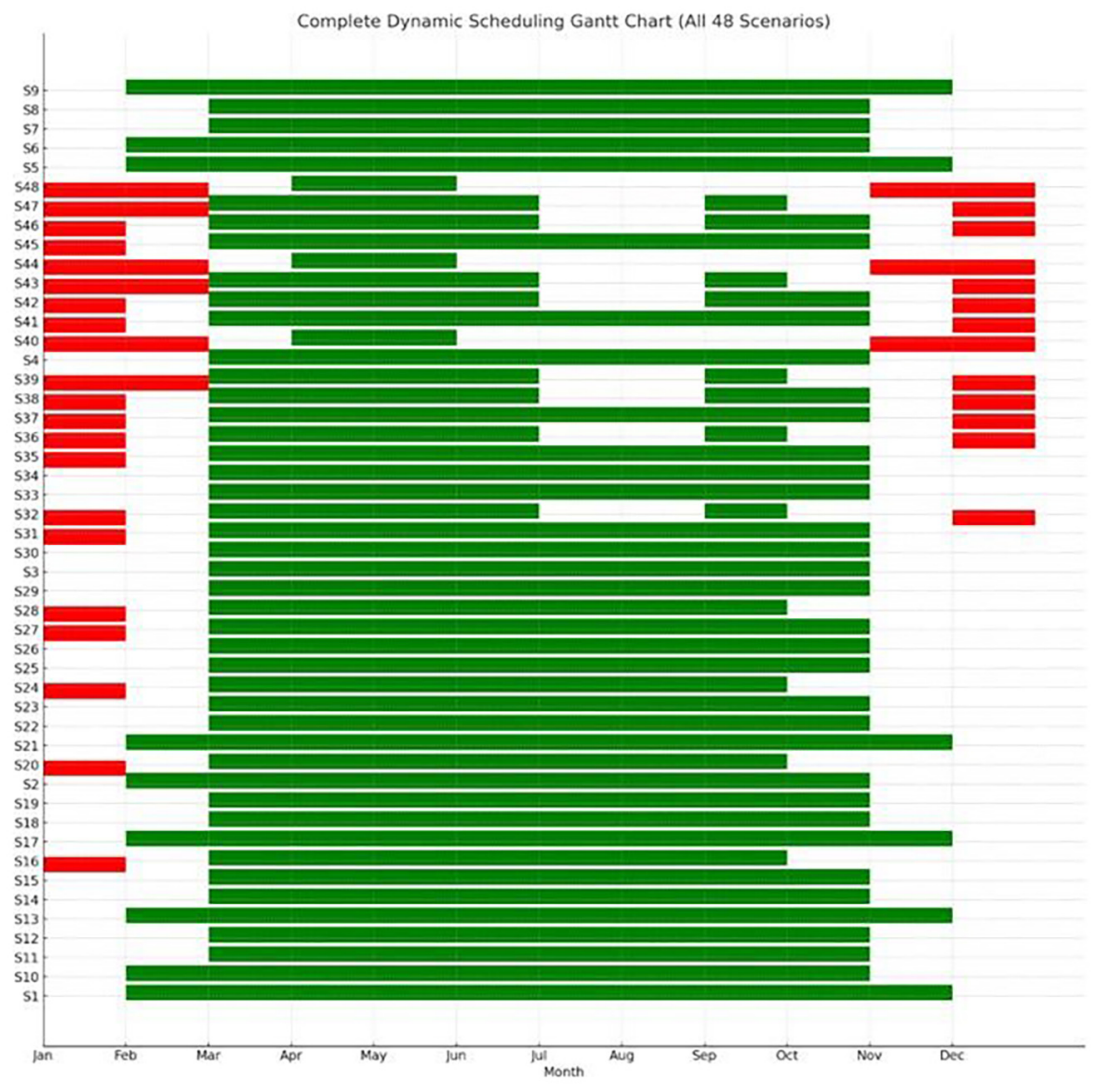
Dynamic scheduling Gantt chart of crop cycles across all scenarios. Green bars mark PV-favorable periods, while red bars highlight grid-stressed months with high energy demand.

To visualize how each crop cycle aligns with solar availability and ambient conditions, Figure 14 provides a dynamic Gantt chart of all scenarios, by providing information based on ascending energy requirements. Green bars indicate PV-favorable months (PV coverage > 60%) which are ideal for starting crop cycles while red bars represent grid-stressed months (PV coverage < 40%) where high-load operations are present. Blank months show a moderate energy balance where standard operations take place. Most high-performance scenarios (e.g., S1–S25) are clustered around the spring and autumn period, which offers favorable HVACD balance and improved PV yield. These scenarios avoid the extreme cooling load demands of summer and the high heating demands of winter. On the other hand, lower-ranked configurations like S4, S8, S12 are scheduled in late autumn or winter, where energy costs per cycle peak, and PV contribution is minimal. This temporal dimension of optimization demonstrates that seasonal timing is as critical as environmental control. Selecting appropriate crop windows allows the system to capitalize on periods of natural thermal neutrality and maximum PV availability, improving both energy independence and carbon footprint performance.

Each row represents one of the 48 scenarios (S1–S48), while the green bars indicate the periods during which optimal crop cycles were scheduled. Red bars highlight energy-deficient periods that were excluded from scheduling due to low PV availability or high energy demand. This Gantt chart visualizes the adaptive temporal distribution of crop cycles throughout the year, according to energy sufficiency and environmental suitability. Chart produced using Python and AI-assisted scheduling analysis.

**Figure 15.**
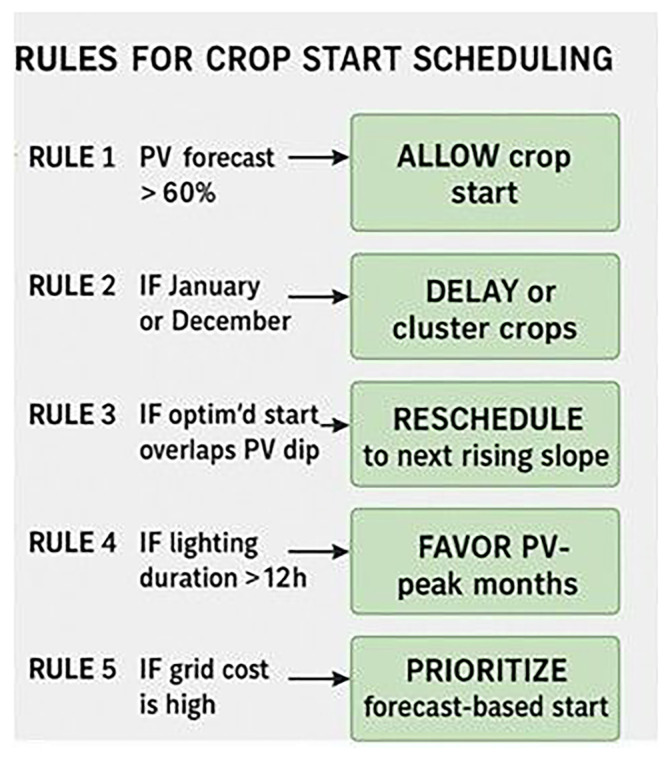
Rules for Crop Starting Scheduling.

To improve the energy efficiency of the VF system, one of the most impactful considerations is the timing of crop cycles. Aligning planting and harvesting schedules with months that are favorable for PV generation—typically March through May and September through October—can significantly reduce reliance on grid electricity. During these periods, solar irradiance tends to be higher, leading to more effective utilization of PV energy. Conversely, planting during winter months, such as December and January, should be avoided due to reduced solar availability and increased dependence on the grid. In regions like Greece, strategic scheduling can reduce grid energy consumption by over 40 percent. Employing a calendar-based Gantt model to plan crop cycles around these optimal solar periods ensures that energy use is aligned with the natural availability of renewable resources. In addition to scheduling, the climate control parameters of the indoor environment play a critical role in managing energy demand. Efficient setpoints for heating, ventilation, and air conditioning (HVAC) systems are essential. Cooling temperatures should be maintained between near 28°C to reduce the energy load on heat pumps for lettuce cultivation. Heating, on the other hand, should be limited to a range of 12°C up to 16°C to minimize energy consumption during colder months. Maintaining a relative humidity of 50% to 60% percent strikes a balance between optimizing plant transpiration and limiting energy-intensive dehumidification or humidification processes. It is also important to avoid extreme internal temperature gradients. Differences greater than 18°C between daytime and nighttime settings can dramatically increase HVAC demand. A dynamic approach to climate control is recommended, where HVAC intensity is adjusted in accordance with seasonal energy availability. Lighting, as one of the most energy-intensive components in vertical farming, must also be managed with precision to improve overall energy efficiency. Ideally, lighting should be concentrated during the daytime to coincide with periods of high PV output, thereby making the best use of self-generated solar energy. Furthermore, lighting intensity should be modulated based on environmental conditions, such as cloud cover, and the availability of energy stored in on-site batteries. During times when HVAC demand is high, lighting loads should be reduced accordingly to avoid overlapping energy peaks, which can place excessive stress on the system and increase reliance on grid electricity. All these recommendations are presented in Figure 15.

Five decision rules are applied to optimize the start date of cultivation cycles. Rule 1 allows crop start when PV forecast exceeds 60%. Rule 2 delays or clusters crops during low-insolation months (January or December). Rule 3 reschedules start dates that coincide with predicted PV dips. Rule 4 favors months with lighting durations >12 hours, while Rule 5 prioritizes forecast-based timing when grid electricity cost is high. These rules form the basis for dynamic Gantt scheduling in the simulation model.

To classify scenarios based on their energy behavior and adaptability, a Principal Component Analysis (PCA) was conducted on key performance indicators derived from dynamic crop-cycle simulations. The input variables included: (i) maximum HVAC energy during crop cycles, (ii) minimum PV availability within the worst energy period, (iii) total energy consumption over full cycles, (iv) optimized energy under ideal scheduling, (v) absolute energy savings from schedule optimization, and (vi) crop cycle duration. These features were chosen to jointly reflect both energy stress and operational flexibility of each vertical farming scenario.

The PCA reduced the six-dimensional dataset into two principal components, which together captured over 80% of the total variance. Principal Component 1 (PC1) was primarily associated with maximum cycle energy and energy savings (both negatively), while showing a slight positive correlation with crop cycle length and optimized energy use. This axis effectively separates scenarios based on energy intensity and flexibility. Scenarios with strongly negative PC1 scores exhibit high baseline energy demand but strong optimization potential, whereas those on the positive end are generally stable, lower-energy systems with flatter energy profiles and minimal gains from dynamic intervention. Principal Component 2 (PC2) captured the trade-off between cycle duration and optimized energy. It correlated positively with crop cycle length, and negatively with optimized cycle energy, distinguishing longer-duration, efficient scenarios from short-cycle, high-demand cases. Together, PC1 and PC2 define a spectrum from short, intensive, energy-heavy setups to long-duration, energy-optimized configurations. To further interpret the scenario landscape, k-means clustering (k=4) was applied to the PCA-transformed data, resulting in four distinct clustering groups:

- Cluster 0: High Demand, Rigid - Scenarios with heavy HVAC and lighting loads but limited optimization response. Likely require infrastructural interventions or high-reliability backup systems
- Cluster 1: Moderate, Responsive - Highly responsive to schedule shifts. Suitable for adaptive lighting or seasonally adjusted planting windows
- Cluster 2: High Demand, Optimizable - Initially high-demand scenarios with excellent response to cycle alignment—ideal for predictive control
- Cluster 3: Stable, Low-Demand - Inherently low-energy systems that show little gain from optimization—potential candidates for passive operation or fixed setpoints

**Figure 16.**
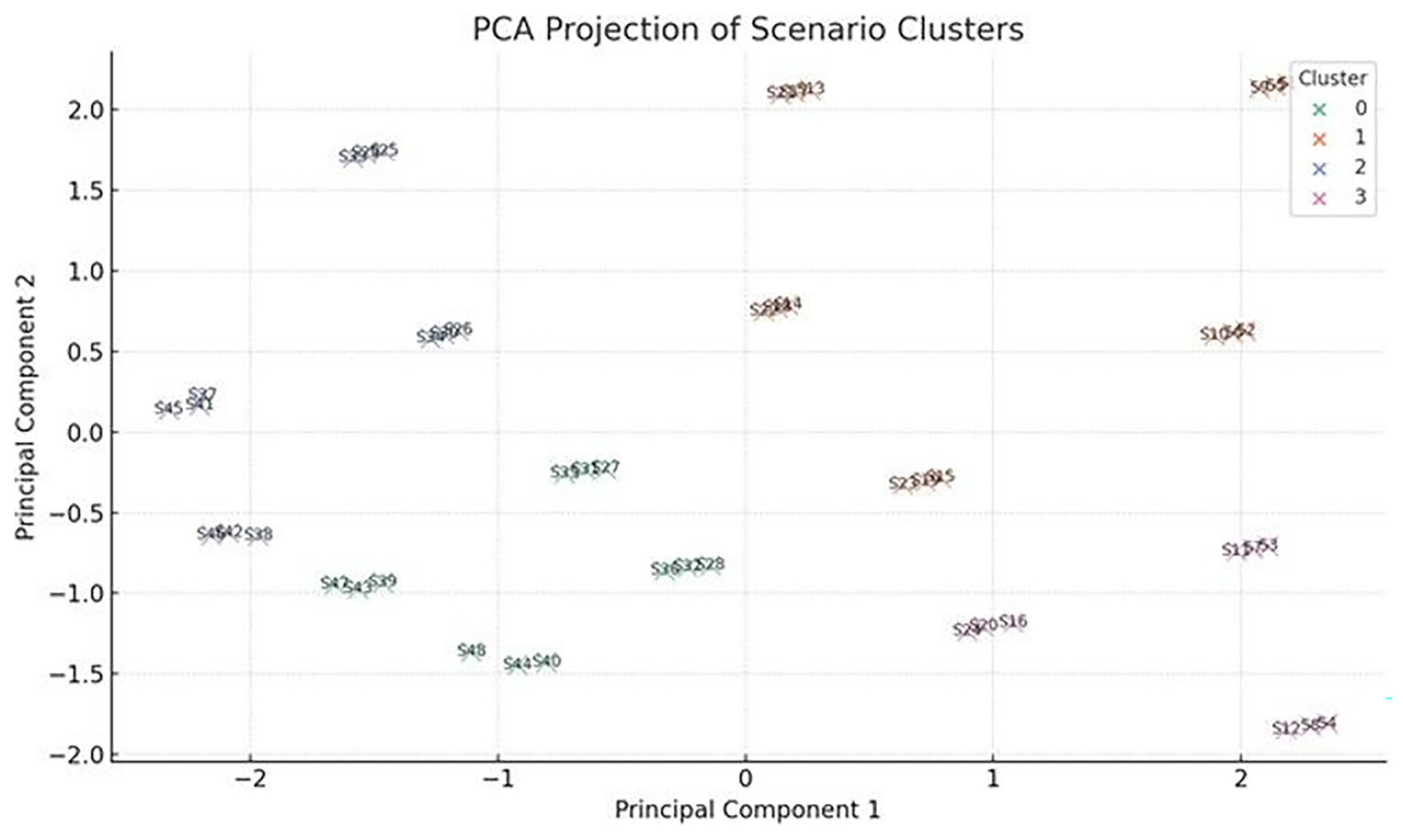
PCA of examined scenarios.

The PCA projection highlights not only the clustering structure but also offers insights into the internal coherence and outliers among the scenarios ( Figure 16). Clusters that are tightly grouped in the PCA space—such as those in the bottom-left quadrant—represent scenarios with highly similar energy profiles, making them suitable for generalization or batch optimization. In contrast, outlying points near the edges of the projection space, such as scenarios S44 and S47, exhibit unique behavior patterns that are unlikely to conform to generalized models and may require custom operational planning or targeted system design interventions. The first cluster, Cluster 0, is characterized by the highest average energy consumption per cycle. These scenarios falling into this Cluster demonstrate relatively optimized energy usage, along with notable energy savings through scheduling adjustments. With an average cycle length of just 37 days, these are short-cycle but high-energy setups. This cluster likely represents crops grown under intense environmental conditions or systems with heavy artificial lighting demands. Cluster 1 consists of scenarios with moderate overall energy consumption, and slightly lower optimized energy levels. These scenarios feature the longest average cycle lengths. The profile of Cluster 1 suggests a group of long-duration crops that respond well to energy optimization, combining operational stability with relatively efficient energy performance. The third group, Cluster 2, stands out due to its extremely high peak energy demand. Despite this intensity, these scenarios are among the most energy-efficient after optimization. Moreover, they have the potential to achieve the highest average energy savings suggesting systems that, while initially demanding, respond exceptionally well to strategic scheduling—likely corresponding to crops that benefit substantially from tailored timing and load balancing. Finally, Cluster 3 presents a contrasting picture, with the lowest recorded energy savings and minimal difference between total and optimized energy use. These scenarios suggest a lack of flexibility, potentially caused by poorly aligned PV availability, rigid operational schedules, or inherently flat energy demand profiles. As such, this cluster may represent VF systems that are either constrained by fixed schedules or offer little room for optimization. Principal Component 1 (PC1) exhibits a strong negative correlation with maximum cycle energy and energy savings, and a slight positive correlation with cycle length and optimized energy. This axis effectively distinguishes between high-energy, high-savings scenarios—typically located on the left side of the projection—and more stable or energy-efficient systems found on the right. PC1 thus captures a gradient of energy intensity and flexibility. Principal Component 2 (PC2), on the other hand, reflects a trade-off between crop duration and energy optimization. It correlates negatively with optimized energy and positively with cycle length. Scenarios situated at the top of the projection typically represent long-cycle, energy-efficient growers, while those at the bottom reflect shorter but more demanding cycles. This suggests that PC2 helps to distinguish between efficient, slow-growing setups and fast but energy-intensive operations. From these cluster characteristics and PCA interpretations, a typology of operational behaviors emerges. The "High Demand, Optimizable" category includes scenarios with substantial energy loads that still benefit significantly from dynamic scheduling or load shifting. In contrast, the "High Demand, Rigid" group represents systems with high energy use but limited adaptability—prime candidates for backup energy systems or aggressive HVAC setpoint tuning. The "Moderate, Responsive" typology includes systems with mid-range demand but high responsiveness, offering a good balance of efficiency and flexibility. Finally, the "Stable, Low-Demand" setups show minimal energy requirements and limited optimization gains, making them ideal for low-intervention, resilient farming operations.

The 48 simulated scenarios are projected in two-dimensional space based on PCA of key performance indicators (e.g., energy consumption, grid dependency, PV sufficiency). Four distinct clusters (C0–C3) are identified using unsupervised learning (k-means), providing insight into scenario typologies and potential trade-offs in resilience, autonomy, and energy optimization. Chart produced using Python and AI-assisted scheduling analysis.

### 4.3 CO _2_ emissions comparison

***4.3.1 Introduction-comparative carbon footprint of vertical, greenhouse, and open-field farming.*** Quantifying and comparing the carbon of farming systems is essential for evaluating their environmental sustainability, especially under the lens of climate change and decarbonization targets. In the context of vertical farming (VF), which relies heavily on artificial energy inputs, understanding the specific emissions per kilogram of output is a critical step toward evaluating its viability against more traditional systems such as greenhouses and open-field agriculture. Among the available metrics, CO _2_ emissions per kilogram of produced lettuce in comparative studies is the most widely adopted indicator. This normalized unit allows for a consistent comparison across systems that differ significantly in scale, energy inputs, and yield densities. It also enables benchmarking of technological improvements, such as the integration of renewable energy systems or optimized climate control strategies. In this section, a comparison of the carbon emissions associated with VF, greenhouse, and open-field production systems takes place based on both published literature and simulation outputs.

***4.3.2 Comparison rationale.*** A comparative literature review of the carbon footprint associated with lettuce production across different agricultural systems reveals significant variation, primarily driven by energy inputs, yield density, and regional grid emission factors. Table 7 summarizes indicative values (in kg CO _2eq_ /kg lettuce) from key studies.

**Table 7.** Overview on carbon footprint emissions for lettuce production.

Source	Production System/Location	CO _2_ Emissions (kgCO _2_/kg lettuce)	Scenario Description
3	Vertical Farming (NL)	15.2	VF unit powered by Dutch electricity mix (high fossil share), full artificial lighting.
	Greenhouse (NL)	2.4	High-yield semi-closed greenhouse with combined heat and power (CHP) and some supplemental lighting.
	Open Field (NL)	0.35	Conventional outdoor farming with minimal mechanization, no artificial lighting.
25	Vertical Farming (EU)	11.6–14.7	Range for fully enclosed systems with LED lighting and mechanical HVAC.
7	Vertical Farming (MY)	6.1–9.4	Hybrid PV-grid system in tropical climate with reduced HVAC demand.
5	Vertical Farming	6.9–12.0	Results vary by lighting intensity and climate (warmer climates yield lower HVAC loads).
6	Vertical Farming	≈9.8	Multiparametric optimization across different energy sources and lighting profiles.
4	VF (Global Range)	8.0–18.0	Aggregated data from multiple studies covering global setups, including worst-case fossil grid mixes.
9	Greenhouse (EU)	2.5–3.2	Range from low-tech to advanced greenhouses with CO2 fertilization and heat recovery.

Several key factors influence the variability of carbon emissions in vertical farming systems, with the most significant being the carbon intensity of the electricity grid. Vertical farms that operate in regions where the electrical grid relies heavily on fossil fuels—such as coal or natural gas—tend to exhibit substantially higher CO _2_ emissions. The energy source behind a vertical farm's electricity supply is therefore a primary determinant of its environmental impact. Lighting, also, represents another major contributor to overall energy consumption in vertical farming. Consequently, the lighting strategy adopted has a direct effect on emissions. Approaches that reduce the Daily Light Integral (DLI), such as spectrum-tuned LEDs or photoperiod adjustments, as well as the implementation of dynamic lighting systems that respond to real-time energy availability or crop needs, can substantially decrease total emissions. Heating, ventilation, air conditioning, and dehumidification (HVACD) systems also play an increasingly important role in emission profiles, particularly in tropical climates or facilities with suboptimal insulation. In such contexts, the energy demands of HVACD systems can rival or exceed those of lighting. Optimizing these systems through the use of energy-efficient heat pumps, and smart thermal setpoint scheduling is essential for minimizing emissions without compromising crop quality or yield. Finally, the method of normalizing emissions data has a significant impact on the interpretation of sustainability metrics. While some studies express emissions per square meter of cultivated area, this approach can obscure meaningful comparisons across systems with varying productivity. In this analysis, all emission values are normalized per kilogram of fresh produce, providing a more accurate and consistent basis for assessing the environmental performance of different vertical farming configurations. This yield-based normalization ensures that emissions are contextualized relative to actual product output, supporting more reliable benchmarking and decision-making.

**Figure 17.**
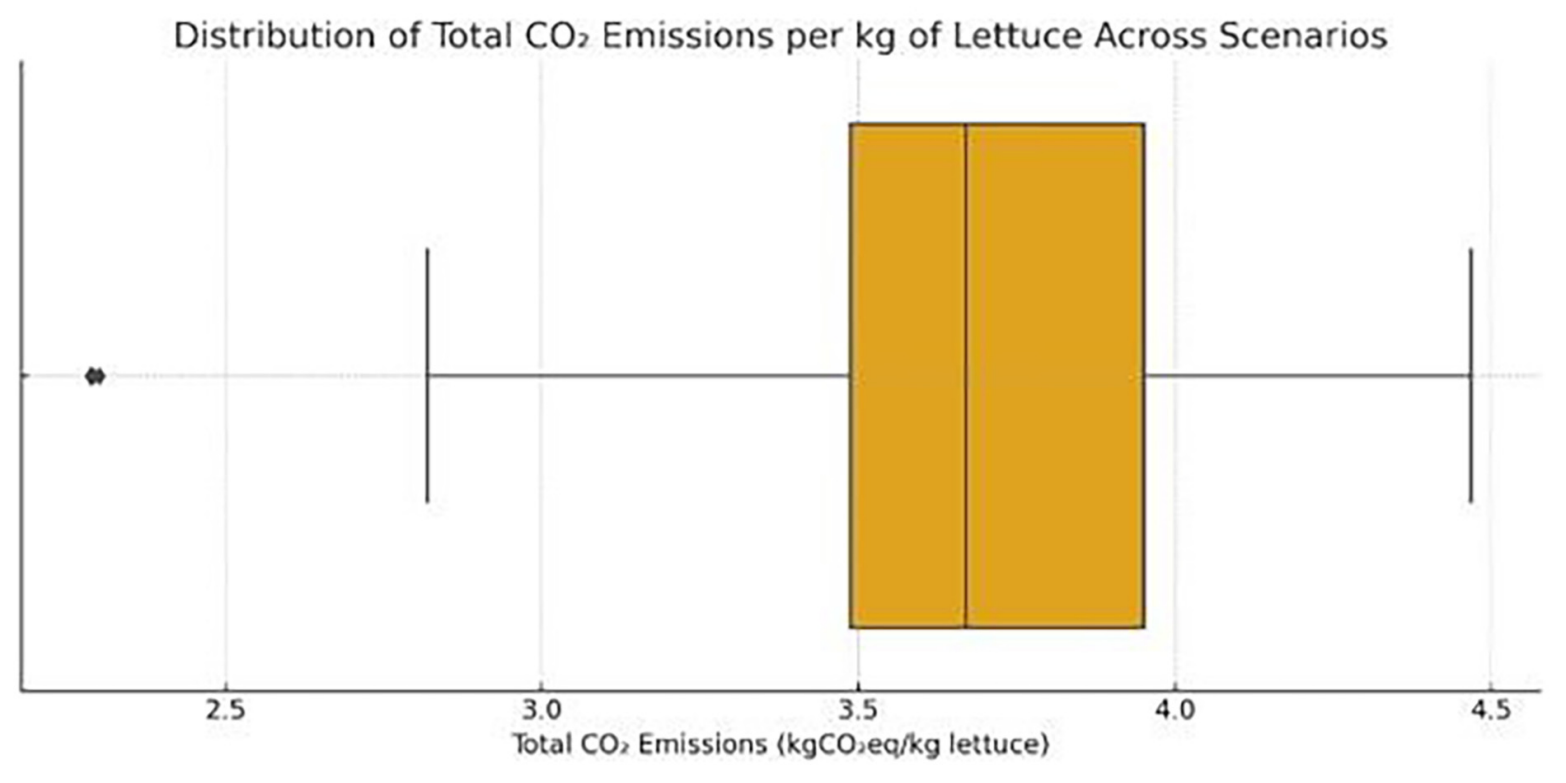
Distribution of CO _2_ emissions across all scenarios. Most values cluster between 3.2–3.8 kgCO _2_eq/kg lettuce, with outliers below 3 and above 4.

***4.3.3 Modeled carbon footprint of vertical farming scenarios.*** The estimated carbon footprint associated with the production of lettuce under all different operational scenarios of the VF system studied in this work varied significantly, with values ranging from a minimum of 2.29 kgCO _2_eq/kg lettuce to a maximum of 4.47 kgCO _2_eq/kg lettuce equivalent per kilogram of lettuce as discussed in Section 3.4. On average, the modeled scenarios yielded an emission intensity of approximately 3.61 kgCO _2_eq/kg lettuce. To gain a more comprehensive understanding of the variability in carbon emissions across all cultivation scenarios, a box plot was employed ( Figure 17). This representation captures the full distribution of the calculated CO _2_ emissions per kilogram of lettuce across the 48 scenarios. Figure visualization reveals that while the majority of scenarios fall within a moderate emission range, specific scenarios—with smallest lighting durations—achieve significantly lower emissions, approaching or even dropping below 2.5 kg CO _2_/kg. The median value is positioned around 3.67 kg CO _2_/kg, indicating that significant number of scenarios (34 out of 48) have lower CO _2_ emissions than the average value for all scenarios.

The boxplot summarizes the life-cycle CO _2_ emissions (in kgCO _2_eq/kg of harvested lettuce) considering energy demand, electricity source, and cultivation schedule. The interquartile range highlights variation across scenarios, while outliers indicate specific combinations of suboptimal scheduling and low PV sufficiency. Chart produced using Python and AI-assisted scheduling analysis.

### 4.4 Comparative evaluation with existing literature

**Figure 18.**
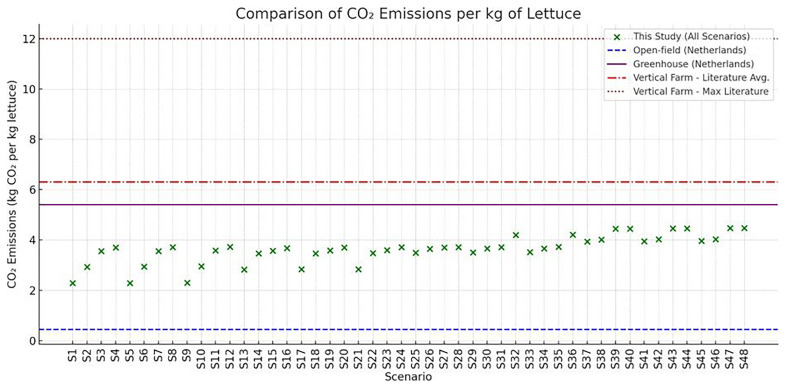
Comparison of CO _2_ per farming option.

The comparative analysis of CO _2_ emissions between the simulations of the examined VF system scenarios and established literature benchmarks reveals valuable insights into the environmental efficiency of the system. Previous studies have reported wide ranges of CO _2_ emissions per kilogram of fresh lettuce, depending on the cultivation system employed. Open-field agriculture typically exhibits the lowest footprint, as it relies entirely on ambient conditions and excludes artificial climate control or lighting inputs ( 3, 6). As expected, open-field farming has significantly lowest CO _2_ emissions, however this method limits lettuce production for a specific period in a year. Greenhouse production, offering higher yields and controlled environments, demonstrates average emissions mainly driven by supplemental lighting and heating demands ^8^. Vertical farming systems, on the other hand, present the highest carbon intensities, depending on the system configuration, location and energy mix ( 4, 5). These numbers stem from the near-complete reliance on artificial lighting as well as HVACD operations. Simulation results examined in this study showed that the best-performing scenarios reach values as low as 2.3 to 2.6 kg CO _2_/kg, clearly outperforming average vertical farm benchmarks, and approaching or even surpassing greenhouse performance in terms of carbon efficiency. The median value across all 48 scenarios remains below the upper greenhouse threshold and well within the reported vertical farming range. Additionally, the carbon intensity of the local electricity grid emerges as a critical variable, aligning with findings from earlier work ( 7, 25). For instance, a low-emission grid or partial integration of photovoltaics and battery storage, as concluded in the examined scenarios, may yield even lower CO _2_ emissions. Ultimately, while vertical farming still struggles to compete with open-field agriculture in carbon terms, our findings show that its footprint can be brought to a competitive level with greenhouses—especially when environmental control is optimized and renewable energy integration is enhanced. Figure 18 illustrates the comparative CO _2_ emissions per kilogram of fresh lettuce produced under four distinct systems: open-field cultivation, greenhouse production, vertical farming based on literature data, and all 48 scenarios simulated in this study. The results demonstrate that the majority of scenarios are clustered around 3.2–3.8 kg CO _2_/kg. These values are substantially lower than the average CO _2_ emissions reported in the literature for vertical farming, which typically range from 6.2 kg CO _2_/kg (average) up to 12 kg CO _2_/kg (maximum). Notably, the lowest-emission scenarios in this study (e.g., S1, S5, S9) approach the levels observed in greenhouse systems (5.4–5.5 kg CO _2_/kg) and, in rare cases, achieve emissions less than half of those reported in prior VF studies. Thus, greenhouse systems offer a middle ground, balancing environmental control with moderate energy use. In comparison, open-field systems maintain the lowest emissions footprint (~0.35kg CO _2_/kg), owing to minimal energy input but suffer from climate dependency and limited spatial efficiency.

Each green cross represents a scenario from this study, spanning a range of energy strategies and crop cycle timings. The blue dashed line indicates the reported emissions from open-field cultivation in the Netherlands, the red dashed-dotted line represents emissions from greenhouse cultivation, while the magenta solid line and red dotted line represent the average and maximum values reported for vertical farms, respectively. Several scenarios in this study achieve substantially lower emissions than most vertical farming benchmarks, highlighting the importance of optimized scheduling and PV integration. Chart produced using Python and AI-assisted scheduling analysis

### 4.5 Impacts of dynamic lighting control on energy and crop cycle duration

To enhance PV self-utilization and minimize grid dependency, a dynamic lighting strategy is implemented that selectively reduces artificial lighting during low-irradiance (“cloudy”) days. Cloudy days were identified by applying a threshold-based condition on daily PV generation data. Specifically, days with production values below the 10th percentile of the annual PV generation profile or more than one standard deviation below the seasonal average were flagged as “cloudy”. This method served as a proxy for reduced solar availability in the absence of direct irradiance data. On such days, lighting energy demand was proportionally reduced by 2.5%, 5%, 7.5% and 10%, resulting trade-offs in crop cycle duration were estimated using a regression model linking photoperiod to crop growth rate. The dynamic model ensures lighting reduction is only triggered when PV generation is insufficient, thereby reducing grid load without severely compromising crop timelines. This adaptive strategy offers a realistic and implementable approach to load shifting in vertical farming systems, especially where PV-battery systems are deployed. To support adaptive energy and schedule optimization in vertical farming systems, this work developed a predictive model for estimating crop cycle duration as a function of key environmental control parameters as discussed in Section 3.3. This model can be subsequently used to estimate the impact of dynamic lighting reductions on crop timing under various scenarios. Using the quarter-hour PV generation profile, days with significantly lower PV output compared to the seasonal average were tagged as cloudy or low-solar days. A rolling average threshold (bottom 10–15% of daily PV generation) was applied across the year to isolate these days. Once low-PV days were identified, reducing LED lighting load by 2.5%, 5%, 7.5% and 10%, was proposed, but only on those specific days. This was applied selectively, avoiding days with sufficient PV, to minimize system grid draw. Thus, for

***Cloudy Day Detection Condition***

*P _i_*: *Daily PV energy production on day i μ _P_*: *Rolling or seasonal average PV energy production σ _P_*: *Standard deviation of daily PV generation*

*Τ _P_*: *PV production threshold for cloudy days* ( *e.g.,bottom* 10 – 15%)

*A day is considered “cloudy” if:*


$$P_{i}<\mu_{P}-a\cdot \sigma_{P}\;\text{or}\;P_{i}<T_{P}\;(7)$$

Where:

*a* ∈ [1,1.5]: *sensitivity coefficient*

*T _P_*: *empirically set percentile cutoff* ( *e.g.,*10 *th percentile of annual PV data)*

***Dynamic Lighting Reduction Application:***

*L*: Baseline lighting energy (kWh/day)

*δ* ∈ {0.025, 0.05, 0.075, 0.1}: *lighting reduction factor*

*L'* = *L* ∙ (1 – *δ*): *adjusted lighting consumption on "cloudy" day*

*C: Baseline crop cycle duration* ( *days*)


$$C^{\prime }=C+f(\delta ):adjusted\;cycle\;duration\;via\;regression\;model\;(8)$$

*The extended cycle duration* *C*' *is estimated using a linear model:*


$$C^{\prime }=a\cdot \delta +b\;(9)$$

*Derived from the photoperiod vs. crop duration model*

*By implementing the aforementioned formulas and by adjusting the new photoperiods as follows:*


$$Photoperiod_{\delta }=Photoperiod_{0}\cdot (1-\delta )\;(10)$$

A new cycle length duration is recalculated based on the regression model with the new *Photoperiod _δ_* and for all the scenarios the energy difference between the initial values for energy consumption and the values for energy consumption after the dynamic lighting control were calculated. The new cycle length durations for each scenario are presented in Table 8.

**Table 8.** Lighting Control Strategies Result.

Lighting Control Strategies Results				
Scenario	Max Safe Lighting Reduction (%)	Rounded Crop Cycle (days)	Estimated Energy Use (kWh)	Energy Saved (kWh)
S1	10	54	857.8	22.0
S10	10	51	902.5	25.1
S11	10	48	929.2	31.4
S12	7.5	45	954.3	10.1
S13	10	50	915.8	9.8
S14	10	47	948.1	14.4
S15	7.5	44	942.3	5.6
S16	10	42	951.5	3.5
S17	10	50	916.3	8.8
S18	10	47	947.9	13.4
S19	7.5	44	941.3	4.7
S2	10	51	902.0	26.1
S20	10	42	951.5	3.5
S21	10	50	916.3	8.8
S22	10	47	944.9	14.1
S23	7.5	44	941.2	4.4
S24	10	42	949.4	2.3
S25	10	47	995.7	6.3
S26	7.5	44	994.6	1.2
S27	10	42	1000.9	0.2
S28	2.5	37	966.4	11.0
S29	10	47	994.7	7.4
S3	10	48	929.1	32.4
S30	7.5	44	994.3	1.2
S31	7.5	41	990.9	9.7
S32	2.5	37	965.2	11.7
S33	10	47	994.0	5.2
S34	7.5	44	991.5	0.8
S35	7.5	41	988.3	9.3
S36	2.5	37	964.5	11.5
S37	0	0	0.0	0.0
S38	0	0	0.0	0.0
S39	5	38	1058.3	0.4
S4	7.5	45	953.2	11.6
S40	0	0	0.0	0.0
S41	0	0	0.0	0.0
S42	0	0	0.0	0.0
S43	0	0	0.0	0.0
S44	0	0	0.0	0.0
S45	0	0	0.0	0.0
S46	0	0	0.0	0.0
S47	0	0	0.0	0.0
S48	0	0	0.0	0.0
S5	10	54	857.8	22.0
S6	10	51	902.5	25.1
S7	10	48	929.7	31.4
S8	7.5	45	953.6	10.8
S9	10	54	857.9	21.0

To evaluate the potential for energy optimization through adaptive lighting strategies, each of the 48 scenarios were analyzed to determine the maximum safe lighting reduction -defined as the highest percentage reduction in lighting duration that did not increase total energy consumption due to elongation of the crop cycles’ duration. Based on this threshold, scenarios were classified into five distinct flexibility clusters: Highly Flexible, Upper Moderately Flexible, Low Moderately Flexible, Low Flexible, and Rigid ( Table 9).

**Table 9.** Lighting Flexibility Clusters by Max Safe Reduction.

Max Safe Lighting Reduction Cluster	Flexibility Class	Description
10%	Highly Flexible	Lighting can be reduced to 90% with no penalty. These scenarios are excellent candidates for dynamic lighting controls, especially in response to PV availability or peak load management.
7.5%	Upper Moderately Flexible	Scenarios that tolerate up to 7.5% reduction. These allow meaningful energy flexibility but may require adaptive scheduling to maintain efficiency.
5%	Low Moderately Flexible	Only small reductions are beneficial. Use with caution — partial flexibility possible without increasing HVAC energy.
2.5%	Low Flexible	Very limited flexibility. Best suited for mild demand response or fixed reductions on high-PV days only.
0%	Rigid	No lighting reductions lead to savings. Avoid dynamic lighting — maintain fixed photoperiods to avoid energy penalties due to prolonged HVAC operation.

**Figure 19.**
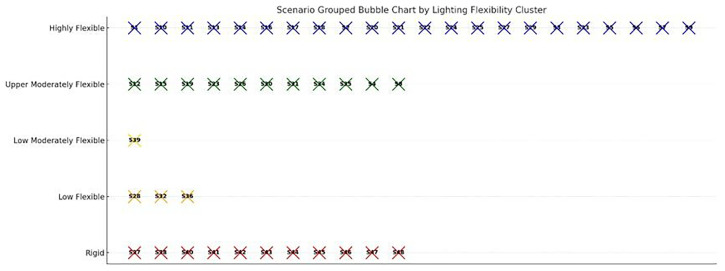
Scenarios grouped by lighting flexibility clusters. Highly flexible cases (blue) tolerate reduced lighting without energy penalty, while rigid cases (red) show no flexibility.

A visual cluster-based bubble chart was constructed, grouping scenarios according to these classifications and clearly highlighting their adaptability to lighting interventions ( Figure 19). Scenarios in the Highly Flexible group (e.g., S1, S2, S13) demonstrated the ability to tolerate up to 10% reduction in lighting without energy penalty, making them ideal candidates for dynamic lighting control strategies that respond to PV availability or energy pricing signals. In contrast, Rigid scenarios (e.g., S37 to S48) showed no flexibility, as even minor reductions led to increased total energy due to extended crop durations and elevated HVAC demands.

Scenarios are categorized into five lighting flexibility clusters: Highly Flexible, Upper Moderately Flexible, Low Moderately Flexible, Low Flexible, and Rigid. This classification reflects the degree to which dynamic lighting strategies can be applied within each scenario to shift energy loads, minimize peak demands, or align with PV availability. Highly flexible scenarios (in blue) demonstrate the greatest potential for energy management optimization through lighting schedules. Chart produced using Python and AI-assisted scheduling analysis.

This clustering approach not only provides insight into the operational flexibility of each scenario but also guides the design of responsive control policies. For example, Low Flexible or Moderately Flexible scenarios may benefit from more conservative or seasonal lighting adjustments, while Highly Flexible configurations can adopt real-time adaptive schedules. This classification is especially valuable when coupled with sustainability metrics, allowing for multi-criteria decision-making based on energy efficiency, CO _2_ emissions, and operational resilience. Conversely, high-demand scenarios, especially those with long photoperiods or extreme HVAC loads, exhibited less flexibility. In these cases, the energy savings from reduced lighting were counterbalanced by increased cycle durations, resulting in little or no net benefit. An aggregate analysis across all 48 scenarios showed that lighting reductions of up to 10% consistently delivered measurable gains in energy savings, cost reduction, and carbon mitigation. However, in many scenarios beyond a threshold (e.g., 5%), the marginal benefits decrease, and the intervention became energetically inefficient due to extended growth durations. These findings confirm the feasibility of integrating dynamic lighting control in vertical farming, especially when guided by real-time PV generation forecasts or demand-response signals.

## 5. Conclusions

This study systematically evaluated the energy performance, environmental impact, and operational flexibility of a vertical hydroponic farming system under Northern Greece climatic conditions through a comprehensive simulation framework. Using TRNSYS 18-based modeling, 48 cultivation scenarios for lettuce production were analyzed, incorporating variations in photoperiod, humidity, HVACD setpoints, and renewable energy integration. A multi-criteria decision framework was introduced encompassing energy per cycle, emissions, and resilience. The results demonstrate that electricity demand in vertical farming is highly sensitive to crop cycle configuration, with artificial lighting and HVACD loads being the dominant contributors. By aligning crop cycles with PV availability, optimized schedules showed measurable reductions in grid reliance and excess generation. These insights were further extended into a proposed dynamic planting calendar and adaptive setpoint strategies for lighting and HVAC systems. Scenarios with longer photoperiods and extreme temperature differentials exhibited higher energy intensity and grid dependency. However, aligning crop schedules with seasons of milder weather conditions—particularly autumn and spring—allowed for improved PV utilization and lower energy demand from grid. The study also revealed marginal variability in carbon emissions across scenarios, ranging from 2.29 to 4.47 kg CO _2_/kg lettuce. Notably, the best-performing configurations achieved emission levels outperforming even greenhouse systems and significantly better than previously reported VF benchmarks. These gains were achieved through strategic timing for optimal energy crop cycles, environmental control and integration with a PV-battery system. A combined assessment using indicators such as Grid Dependency Ratio (GDR), Grid Independence Index (GII), and Seasonal Resilience Score (SRS) enabled a multi-dimensional classification of scenario performance. Scenarios with low grid reliance and stable PV alignment were identified as optimal candidates for future deployment in energy-constrained or off-grid contexts. A key innovation of this work is the Lighting Flexibility Classification, where each scenario was assessed for its capacity to safely reduce photoperiod without increasing total energy demand. Scenarios were grouped into five clusters—Highly Flexible, Upper Moderately Flexible, Low Moderately Flexible, Low Flexible, and Rigid—based on the maximum safe lighting reduction achievable. These classifications offer a practical guideline for implementing dynamic lighting interventions. For instance, scenarios like S1, S2, and S13 were found to tolerate up to 10% lighting reduction with net energy savings, making them ideal candidates for real-time lighting control. On the other hand, Rigid scenarios, primarily those with extreme temperature or humidity requirements (e.g., S37–S48), showed no capacity for lighting reduction without incurring increased HVAC energy costs. Overall, the integration of lighting flexibility, crop cycle adaptation, and renewable alignment advances our ability to design sustainable and intelligent vertical farming operations. The decision tools developed herein—including radar plots, quadrant maps, and bubble cluster charts—can guide farm designers, policy makers, and engineers in selecting optimal environmental strategies. Future work will extend this model to include real-time predictive control using live PV forecasts and feedback from plant physiology to further optimize energy use and crop yield outcomes. In conclusion, the findings confirm that vertical farming, while energy-intensive, can be significantly optimized through seasonal crop scheduling, moderate environmental control, and targeted lighting strategies. Under the right configuration, vertical farms can approach or even outperform greenhouse systems in both energy and carbon metrics, especially when partially powered by renewable sources. Future work should explore the integration of real-time environmental sensing, AI-driven lighting algorithms, and scalable PV-storage systems to further improve system autonomy, productivity, and sustainability. The multiple regression model shows that, when humidity levels remain within the acceptable range for proper plant growth, the scenarios indicate that variations in indoor humidity have a negligible impact on energy consumption. A key innovation of this work is the Lighting Flexibility Classification, where each scenario was assessed for its capacity to safely reduce photoperiod without increasing total energy demand. Scenarios were grouped into five clusters—Highly Flexible, Upper Moderately Flexible, Low Moderately Flexible, Low Flexible, and Rigid—based on the maximum safe lighting reduction achievable. These classifications offer a practical guideline for implementing dynamic lighting interventions. For instance, scenarios like S1, S2, and S13 were found to tolerate up to 10% lighting reduction with net energy savings, making them ideal candidates for real-time lighting control. On the other hand, Rigid scenarios, primarily those with extreme temperature or humidity requirements (e.g., S37–S48), showed no capacity for lighting reduction without incurring increased HVAC energy costs. Overall, the integration of lighting flexibility, crop cycle adaptation, and renewable alignment advances our ability to design sustainable and intelligent vertical farming operations. The decision tools developed herein—including radar plots, quadrant maps, and bubble cluster charts—can guide farm designers, policy makers, and engineers in selecting optimal environmental strategies. Future work will extend this model to include real-time predictive control using live PV forecasts and feedback from plant physiology to further optimize energy use and crop yield outcomes.

## Ethics and consent

Ethics approval and consent are not required

## Data Availability

**Zenodo**: Dynamic Energy Optimization and Lighting Flexibility Classification for Sustainable Vertical Farming: A Simulation-Based Multi-Scenario Analysis https://doi.org/10.5281/zenodo.16926135 ^26^ This project contains the following underlying data: TRNSYS 18 input files (tpf/log/txt/pti/lst/idf/dck/bld/plt) input configuration files used for running the 48 cultivation scenarios. Environmental Datasheets (tm2) raw environmental data used as model inputs Simulation Results (txt) raw and processed outputs from TRNSYS 18 simulations across all scenarios. Figure values (csv) data underlying figures and plots presented in the article **Zenodo**: Dynamic Energy Optimization and Lighting Flexibility Classification for Sustainable Vertical Farming: A Simulation-Based Multi-Scenario Analysis https://doi.org/10.5281/zenodo.16926135 ^26^ Equipment Manuals (pdf) Manuals for the existing equipment in the existing isobox Python Scripts for scenario analysis (txt) Scripts used to process simulation results Scenarios and supplementary tables and data (xlsx/csv) All data for the 48 scenarios analysis Figures and Scenarios optimal crop profiles (jpg/png) All resulting figures from the analysis Tables (docx) All tables used in the final paper The authors confirm that no applicable reporting guidelines (e.g. CONSORT, STROBE, PRISMA) exist for the simulation-based methodological study presented herein as verified through the EQUATOR and FAIRSharing databases. Data are available under the terms of the Creative Commons Attribution 4.0 International license (CC-BY 4.0). Due to software licensing restrictions and proprietary dependencies of TRNSYS 18, the complete project files (including component libraries and simulation workspace) cannot be shared publicly. Sharing these files would require distribution of third-party licensed material that is not permitted under open access. These files are part of the licensed TRNSYS software package, which is distributed by the Transsolar Software Engineering: trnsys.de. Corresponding personnel: Alina Wagner email: hotline@Transsolar.com. Access to these resources require a valid TRNSYS licence.
