# Dynamic Energy Optimization and Lighting Flexibility Classification for Sustainable Vertical Farming: A Simulation-Based Multi-Scenario Analysis

**DOI:** 10.12688/openreseurope.20847.2

**Published:** 2026-05-06

**Authors:** Chrysovalantis Ketikidis, Petros Dallas, Aristotelis Triantafyllidis, Christina Makri, Panagiotis Grammelis

**Affiliations:** 1 CPERI, Ethniko Kentro Ereunas & Technologikes Anaptyxes, Ptolemaida,, Dytiki Makedonia, 50200, Greece

**Keywords:** Vertical Farming, Lettuce Cultivation, Photovoltaic Integration, TRNSYS 18 Simulation, Energy Optimization, Carbon Footprint

## Abstract

**Background:**

Vertical farming offers a promising solution to food production challenges in urban and climate-constrained regions, yet its high energy demand raises concerns about sustainability. Most existing studies assess energy demand and CO _2_ emissions under static operational assumptions and lack a comprehensive framework linking seasonal renewable availability, crop cycle timing, and operational flexibility to system resilience and grid dependency.

**Methods:**

This study evaluates the performance and carbon footprint of a fully enclosed pilot vertical farming unit in Northern Greece using TRNSYS 18 simulations and high-resolution environmental data. Forty-eight cultivation scenarios were generated by varying photoperiods, humidity levels, and HVAC setpoints to reflect seasonal Mediterranean conditions. Each scenario was analyzed for energy consumption, grid reliance, photovoltaic sufficiency, and life cycle CO _2_ emissions. A dynamic crop cycle estimation model was applied to capture seasonal variability and align planting windows with solar energy availability. Performance benchmarking was implemented through a novel resilience-based multi-criteria framework combining energy-per-cycle metrics with two composite indices, the Grid Independence Index (GII) and the Seasonal Resilience Score (SRS), formulated in this study as a key methodological innovation. A Lighting Flexibility Classification System was further developed to determine the maximum safe reduction in artificial lighting without increasing total energy demand.

**Results:**

The median carbon footprint across the 48 simulated scenarios was 3.67 kg CO _2_ kg ^−1^ of lettuce (Lactuca Sativa), with optimized configurations achieving substantially lower emissions compared to conventional greenhouse cultivation. Crop cycle duration varied between 34 and 52 days depending on photoperiod and temperature setpoints. Aligning cultivation cycles with high solar availability, combined with dynamic lighting adjustments, PV contribution to annual electricity coverage was significantly enhanced. In such configurations, lighting inputs were reduced by up to 10% without causing an increase in total system energy requirements.

**Conclusions:**

The findings highlight the potential of simulation-based design for optimizing energy use and minimizing environmental impacts in control-environment agriculture. The proposed metrics and classification provide practical tools for improving the resilience and sustainability of vertical farming under Mediterranean conditions.

## Plain Language Summary

This study looks at how vertical farming, growing crops in stacked indoor systems, can become more energy-efficient and environmentally friendly. Using a test facility in Northern Greece, simulation scenarios of 48 lettuce-plantings were produced with different lighting and climate settings. The electricity production was measured, along with calculations of the amount of energy coming from solar panels, and the amount of carbon dioxide (CO _2_) released. This study also examined the best planting periods based on seasonal sunlight, tools to rate how independent the system was from the electricity grid and how flexible the lighting setup can be. Overall, the study shows that vertical farms can reduce their environmental impact with smart planning, especially when renewable energy is used.

## 1. Introduction

The increasing demand for sustainable food production in densely populated urban areas has led to a growing interest in Vertical Farming (VF) systems. These closed-loop, climate-controlled environments enable year-round crop cultivation with minimal land use, reduced water consumption through hydroponic systems, and the elimination of pesticides and herbicides. ^1^^,^^2^ Vertical farms can yield significantly higher productivity per square meter compared to traditional open-field agriculture, making them a promising solution for future food security. ^3^ This productivity advantage, however, is accompanied by a well-documented trade-off in energy demand. Previous comparative studies have shown that while vertical farming systems achieve substantially higher yield per unit area than greenhouse (GH) cultivation, they also require significantly higher energy inputs, primarily due to artificial lighting and intensive climate control. Such findings underline the fundamental challenge of balancing yield intensification with energy efficiency in controlled-environment agriculture. Ch. Vatistas et al. ^4^ compared the lettuce cultivation between GHs and VFs and presents that VFs are more efficient than GHs in terms of yield per unit area, but require more energy to operate. Lighting alone, mostly provided by LED luminaires, accounts for 60–80% of total electricity consumption in typical VF setups. ^5^^,^^6^ Artificial lighting has therefore received significant attention in recent research, with studies analyzing photoperiod, Photosynthetic Photon Flux Density (PPFD), and lighting efficiency in relation to crop yield and economic performance. Such works demonstrate that lighting strategies can substantially influence productivity and operating costs. Nevertheless, lighting optimization is often evaluated independently of broader system interactions, such as HVAC demand, seasonal renewable availability, or grid dependency, limiting its applicability for holistic energy-resilient vertical farming design. ^7^ This heavy energy burden challenges the economic viability and environmental sustainability of VF, especially in regions with high electricity costs or low renewable energy penetration. To address these challenges, recent research efforts have focused on optimizing energy use through simulation-based design, renewable energy integration, and advanced control strategies. Energy modeling tools offer the ability to evaluate lighting regimes, thermal loads, and storage performance under different operating conditions. ^8^ Furthermore, integrating photovoltaic (PV) systems with battery storage and MPPT inverters has been proposed as a potential solution to enhance energy autonomy and reduce reliance on grid electricity. ^9^^,^^10^ Recent studies have explored near-zero and agrivoltaic configurations for controlled-environment cultivation, demonstrating the technical feasibility of coupling artificial lighting with on-site photovoltaic generation and storage systems. These works highlight the importance of system sizing and energy balance in reducing grid dependency and emissions, particularly in protected or enclosed growing systems. However, such approaches typically rely on fixed operational assumptions and do not explicitly address seasonal variability, crop cycle timing, or adaptive control strategies under fluctuating renewable availability. ^11^ To maximize the Vertical Farming benefits, the applicant must integrate energy-efficient systems and renewable energy sources to remain competitive against traditional agriculture powered by natural sunlight. J. Pimentel’s study ^8^ explored different strategies for energy optimization to be integrated into urban infrastructure, utilizing a multipored model. The results showed that optimizing resource allocation and operation plans can lead to electricity cost savings of up to 40% daily and 31% annually. A case study in Malaysia assesses solar/hybrid/storage energy solutions, demonstrating that grid-connected solar PV systems can support up to 11.6% of VF energy demand while reducing dependency on utility grids and lowering CO _2_ emissions. This study highlights the economic viability of renewable energy integration through Levelized Cost of Energy (LCOE) analysis. Technoeconomic assessments of indoor cultivation systems powered by renewable energy have further demonstrated the potential for reducing operational emissions and costs under optimized configurations. While these studies provide valuable insights at the system or crop level, they generally focus on single-case analyses and do not offer transferable indicators to compare operational resilience, flexibility, or grid dependency across multiple scenarios and seasonal conditions. ^12^ Despite the growing body of literature on energy optimization, renewable integration, and lighting efficiency in controlled-environment agriculture, existing studies typically address these aspects in isolation. A comprehensive framework that jointly evaluates seasonal renewable availability, crop cycle timing, and operational flexibility, while providing quantitative indicators for grid dependency and operational resilience, remains lacking. This study addresses this gap by proposing a multi-scenario, simulation-based framework that integrates dynamic crop cycle scheduling, photovoltaic energy modeling, and novel performance metrics to support climate-resilient and low-carbon vertical farming operation. This study aims to assess the energy efficiency of a VF system under a Mediterranean climatic context through a scenario-based analysis. Using TRNSYS 18, 48 crop cycles are simulated to quantify lighting and HVAC energy demands. Scenarios include different photoperiod strategies and the integration of renewable energy systems. The objective is to identify optimal operational configurations that minimize energy use per unit of harvested biomass, contributing to the broader effort of improving VF sustainability. The key innovations of this work include the introduction of resilience indicators and a Lighting Flexibility Classification System for vertical farming operations.

## 2. Scope of the study

**Figure 1.**
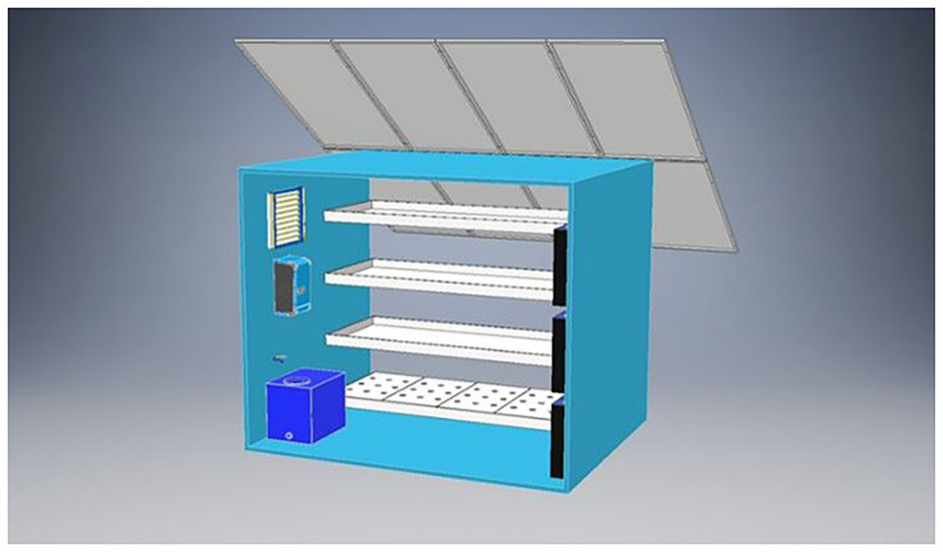
3D visualization of the pilot vertical farming unit.

This study investigates the energy performance and carbon footprint of a fully controlled vertical hydroponic system designed for lettuce cultivation ( Figure 1). Figure 1 shows the analyzed pilot vertical farming unit, comprising four cultivation shelves with LED lighting and integrated HVAC and nutrient delivery systems. Solar panels are mounted on top to enable partial energy autonomy. The internal layout includes environmental sensors, a nutrient reservoir (bottom left), and control electronics (left wall). The sketch was provided by the manufacturer. The analysis is based on dynamic simulations performed using TRNSYS 18, a transient system simulation tool, under a set of predefined technical configurations and operational conditions. The examined system operates in a Mediterranean area, specifically simulating climate data corresponding to Ptolemaida, Greece. Environmental control includes artificial LED lighting, HVACD systems (Heating, Ventilation, Air Conditioning, and Dehumidification), and automated irrigation via nutrient film technique (NFT) which is an active hydroponic system where plants are grown with their bare roots submerged in a shallow stream of nutrient-rich water that continuously circulates through watertight channels or gullies. This method uses a pump to deliver nutrients and water, while the shallow, oxygenated film ensures plants receive water, nutrients, and oxygen. NFT systems are water-efficient, require less space than other hydroponic methods, and are particularly suited for leafy greens and other fast-growing crops. ^1^ All simulations consider a fully enclosed environment, isolated from external natural light. In such fully enclosed vertical farming systems, thermal and moisture loads generated internally cannot be passively dissipated. Artificial lighting constitutes as a source of sensible heat, while crop evapotranspiration represents also a contributor to latent heat and humidity accumulation. Without active climate control, temperature and relative humidity would rapidly exceed optimal thresholds, leading to condensation, plant stress, and increased disease risk. Consequently, the operation of a heating, ventilation, air conditioning, and dehumidification (HVACD) system is a functional requirement rather than a design choice, ensuring stable microclimatic conditions suitable for lettuce cultivation The main focus of the analysis lies in the evaluation of Specific Energy Consumption (SEC), defined as electricity consumption per unit of harvested yield (kWh kg ^−1^), taking into account the impact of different operational scenarios, 48 in total, that vary in crop cycle duration, lighting profiles, and environmental parameters. Additionally, the study assesses the sufficiency of an integrated photovoltaic (PV) system with battery storage in covering the system’s energy demands, both on an annual and seasonal basis. The analysis is based on the operational configuration of a pilot-scale vertical hydroponic unit, which provides the technical specifications and boundary conditions used in the simulation framework. These include the cultivation layout, system sizing, and representative energy consumption characteristics of the vertical farming installation. To place the performance of the examined system within a broader context, the results of the study are interpreted in relation to typical energy-use values reported in the literature for different agricultural production systems:

• **Open-field agriculture:** minimal energy use (≈0.35 kWh kg ^−1^ lettuce), no artificial lighting, but high dependence on climatic conditions. ^13^
• **Greenhouse cultivation:** moderate energy footprint (≈5.4 kWh kg ^−1^), with partial environmental control and occasional supplemental lighting. ^1^
• **Conventional vertical farming (grid-powered):** high energy intensity (≈10–12.5 kWh kg ^−1^), dominated by artificial lighting and climate control requirements. ^6^^,^^8^
• **Hybrid vertical farming systems with photovoltaic support:** potential for up to 50% energy self-sufficiency depending on location, crop scheduling, and storage capacity. ^9^^–^^13^

This comparative perspective allows the investigated vertical farming system to be positioned within the broader landscape of controlled-environment agriculture and sustainable food production systems. The carbon footprint is calculated based on energy consumption and regional emission factors, allowing a comparative assessment with conventional farming systems. The potential benefits of dynamic lighting control strategies are also explored, as a means to reduce overall consumption without affecting yield.

## 3. Methodology

In order to assess the energy performance and sustainability potential of a vertical farming (VF) system, this study employs a scenario-based dynamic simulation framework that integrates environmental control parameters, crop cycle variation, and renewable energy contributions. The analysis focuses on a fully enclosed pilot-scale vertical farming unit, where environmental conditions are entirely controlled through artificial lighting and HVACD operation. The system operates with a self-consumption priority, where PV supply is directed first to loads, then to the to the battery, and finally to the grid. The methodological approach is designed to capture the complexity of VF operations by combining system-level energy modeling, crop growth cycle configurations, and photovoltaic energy supply analysis. The analysis unfolds across multiple stages. Initially, a comprehensive set of cultivation scenarios is defined, incorporating variations in cycle duration, lighting regimes, and harvest frequency. These scenarios are then used as input into a detailed simulation environment developed in TRNSYS 18. The model replicates the core subsystems of a VF unit. The system includes controlled-environment parameters, such as artificial lighting, HVACD (heating, ventilation, air conditioning, and dehumidification, in this case a heat-pump), circulation pumps and fans. The main technical components and auxiliary equipment, are summarized in Table 1. Simulations were run in TRNSYS 18 at 15 min timestep. For weather inputs Meteonorm data were used. Type 56 of TRNSYS components was used in order to simulate the ISOBOX (vertical farming unit) as a single zone building, where dehumidification modeled via latent load removal. First, the cultivation framework was defined based on lettuce ( *Lactuca sativa*) as the model crop, selected due to its widespread use in controlled-environment agriculture and its well-documented sensitivity to environmental parameters such as photoperiod and temperature. Literature-derived cultivation conditions and pilot-scale operational data were used to establish representative environmental ranges and production parameters for the simulation scenarios. Next, the system setup and simulation architecture were defined in order to represent the structural and operational characteristics of the pilot vertical farming unit. This step includes the configuration of the energy system components, environmental control mechanisms, and renewable energy integration within the TRNSYS simulation environment.

**Table 1.** VF Equipment Characteristics.

Vertical Farming System Equipment													
*Pumps*		*Battery 2 V for our case 24 are used*		*Inverter*		*PV Characteristics*		*Heat Pump Technical Specifications*		*LED Lights*		*Fans*	
Types	Values	Specification	Per Cell	Model	Voltronic Axpert VM III 5000–48	Model Name	LX-430M108H (N-Type Bifacial)	Nominal Cooling Capacity	3.4 kW (1.4–4.0 kW range)	Model	MARSHYDRO HYDRO	Model	PF28080R
Flow Rate (m ^3^/h)	1	Nominal Voltage	2 V	Nominal Power	5000 VA/5000 W	Cell Type	Monocrystalline, Half-Cut, Bifacial	Nominal Heating Capacity	4.0 kW (1.4–5.2 kW range)	Power Consuption	300 W	Dimensions	280 × 280 × 80 mm
Head (m)	45	Capacity C120 (1,85 V/cell, 20 °C)	605 (Ah)	Output Voltage	230 VAC	Module Efficiency	22.02%	Cooling Power Input	~0.80 kW	PPFD at 30 cm	940 μmol/m ^2^/s	Voltage	230 V AC
Power (kW)	0.55	Capacity C120 (1,80 V/cell, 20 °C)	422 (Ah)	Output Frequency	50/60 Hz	Nominal Power (Pmax)	430 Wp	Heating Power Input	~0.99 kW	PPFD at 45 cm	780 μmol/m ^2^/s	Power Consumption	130 W
Current (A)	1.6	Recommended Float Voltage	2.23 V	Battery Voltage	48 VDC	Open Circuit Voltage (Voc)	38.93 V	Seasonal Efficiency (SEER/SCOP)	SEER ≈ 8.65 (Cooling), SCOP ≈ 5.10 (Heating)	Spectrum	Full Spectrum (IR + Red+Blue)	Current	0.35 A
Voltage (V)	230	Boost Charge Voltage	2.40 V	Max Charging Current	80 A	Short Circuit Current (Isc)	14.01 A	Nominal EER/COP	EER ≈ 4.23; COP ≈ 4.04	LED Type	Samsung + Epistar Diodes	Speed (RPM)	2,600 rpm
Rotational Speed (rpm)	2850	End-of-Discharge Voltage (C10 rate)	1.80 V	PV Input Voltage Range	60–145 VDC	Maximum Power Voltage (Vmp)	32.65 V	Operating Refrigerant	R-32 (environmentally efficient)	Cooling	Passive aluminum heat	Airflow	1,800–1,980 m ^3^/h (≈ 500–550 L/s)
Impeller Diameter (mm)	140	Internal Resistance	0.64 mΩ	Max PV Array Power	4000 W	Maximum Power Current (Imp)	13.17 A	Airflow (Cooling Mode)	~276–738 m ^3^/h (multiple fan speeds)	Dimming	0–100% knob dimming	Noise Level	68 dBA
Weight (kg)	8.5	Short Circuit Current (each)	3170 A	Max PV Input Current	66 A	NOCT (Nominal Operating Cell Temperature)	45 ± 2 °C	Indoor Unit Noise	Very low (bedroom/office suitable)	Waterproof Rating	IP65	Bearing Type	Ball bearings
		Cycle Life 60% DOD (20 °C)	2000 cycles	Efficiency	93% (peak)	Temperature Coefficient (Pmax)	−0.29%/°C	Controls & Comfort Features	Inverter compressor, intelligent motion sensor, Wi-Fi control	Dimensions	600 × 300 × 70 mm		
		Self-Discharge Rate (20 °C)	~2.5% per month	Communication Interfaces	RS232, USB, Wi-Fi (optional), dry contact	Module Dimensions	1722 x 1134 x30 mm	Dimensions/Weight (Indoor)	~778 × 299 × 272 mm; ~14.5 kg	Weight	5.2 kg		
		Recommended Temperature Range	10–30 °C					Certifications	EU Label A+++ for both heating & cooling				
		Pole Type	M10										

Subsequently, a set of cultivation scenarios was defined to investigate how variations in environmental control parameters influence crop cycle duration and energy demand in vertical farming systems. The scenarios combine different temperature setpoints, humidity levels, and photoperiod durations representative of controlled-environment lettuce cultivation. Next, dynamic simulations were conducted to evaluate the temporal energy behavior of the vertical farming system under each defined cultivation scenario. The simulation process generated time-series outputs describing energy demand, photovoltaic production, and system operation throughout the annual cycle. A set of resilience indicators was developed to evaluate the degree of energy autonomy and seasonal robustness of the vertical farming system across the examined scenarios. These indicators enable the comparative assessment of photovoltaic contribution and grid dependency under varying operational conditions. Following this, multivariate statistical analysis was applied to classify the simulated scenarios according to their energy performance characteristics. Dimensionality reduction and clustering techniques were used to identify groups of scenarios with similar operational behavior. Finally, an adaptive lighting control strategy was introduced to evaluate the potential of demand-side flexibility in vertical farming operations. This approach examines how selective reductions in artificial lighting under low photovoltaic availability can influence system energy demand and crop cycle duration. This comparative framework allows the proposed model to be contextualized within the broader landscape of sustainable food production systems. This methodological structure ensures that the simulation captures both the technological intricacies and environmental implications of VF operation, providing a solid foundation for evaluating optimization pathways and energy-efficiency strategies. In the following section, the simulation framework is presented in more detail, including the system architecture, component modeling, and scenario parameterization.

### 3.1 Cultivation procedure

The selected crop for this analysis is lettuce ( *Lactuca sativa*), a leafy green that has become a benchmark species in vertical farming studies due to its high responsiveness to microclimate conditions. It is a diploid species (2n = 18), photoperiod and temperature-sensitive, is widespread used in controlled-environment agriculture, and generally classified as a long-day plant. The experimental plant material, lettuce cultivar ROMANIA (Lactuca sativa), was obtained from Gemma Grow, a certified supplier of planting material within the Greek agricultural sector. The delivery was accompanied by a plant passport with the following specifications: A: Lactuca sativa, B: 02319004, C: 32658CINDEX, D: GR, Lot number: C.

**Figure 2.**
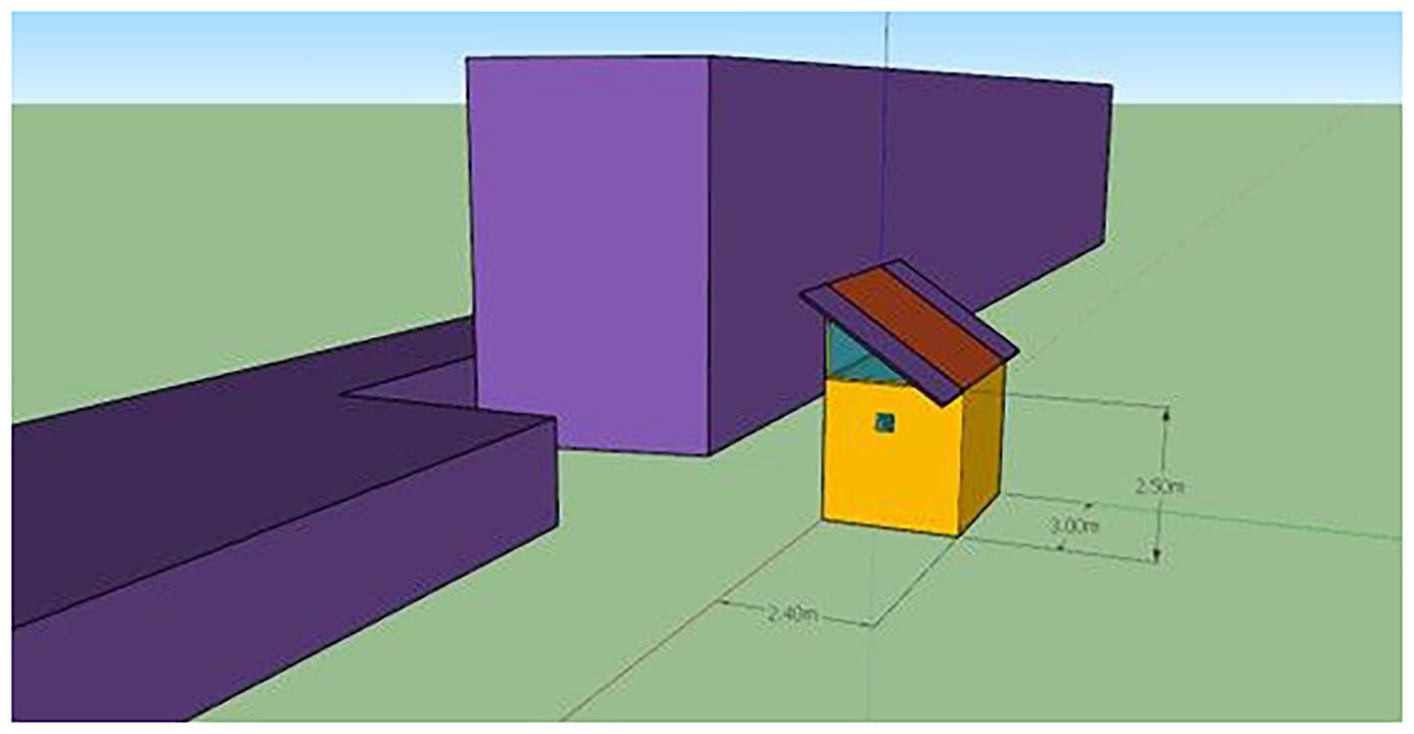
ISOBOX single-zone building exterior. Simplified 3D model of the pilot cultivation chamber (2.40 × 3.00 × 2.50 m) used in TRNSYS simulations, with adjacent blocks representing shading elements.

Vertical farming system is realized in ISOBOX with galvanized metal construction of 4 cm sandwich panel insulation, Aluminium door with LED lights of 1 kW. The dimensions of the ISOBOX are 240 cm × 300 cm × 250 cm (L×W×H) ( Figure 2) as shown in the sketch which will be introduced in TRNSYS 18 software. Figure 2 presents a simplified 3D illustration represents the pilot unit used in the vertical farming study. Dimensions are provided in meters. The orange structure represents the cultivation chamber, while the purple volumes indicate adjacent buildings or shading elements. This schematic was created using SketchUp. The cultivation area comprised four vertical hydroponic layers capable of housing up to 200 lettuce heads per cycle, producing approximately 40–45 kg of lettuce per cultivation cycle. Τhe harvested mass per cultivation cycle was assumed constant across all scenarios, based on pilot-scale operation data and literature-reported fresh weight at physiological maturity for lettuce cultivation. To become more sustainable, VFs must reduce energy demand and develop energy-efficient systems. Table 2 presents a comparative overview of reported cultivation parameters for lettuce (Lactuca sativa) from the literature. These include photoperiod (in hours), humidity (%), temperature (°C), and total cultivation duration (in days), along with the cultivation system employed. This dataset serves as a benchmark for evaluating the environmental conditions used in this study’s simulation scenarios. Where applicable, CO _2_ photosynthetic photon flux density (PPFD) values are reported. Optimal growth has been observed under light intensities with photoperiods of 16 hours. As shown in Table 2, photoperiods between 12 and 20 h are commonly reported in vertical farming and indoor controlled-environment studies for lettuce cultivation. Based on the lighting conditions described in these studies, the applied 16 h photoperiod corresponds to photosynthetic photon flux density (PPFD) levels typically ranging between 200 and 350 μmol·m ^−2^·s ^−1^, resulting in daily light integral (DLI) values of approximately 11–20 mol·m ^−2^·day ^−1^. This range is widely reported as suitable for lettuce growth under controlled-environment agriculture, supporting efficient biomass accumulation without inducing photoinhibition. Photoperiods exceeding 18 hours promote bolting, particularly under elevated temperatures. Optimal growth is achieved at 18–22 °C during the light period and 10–15 °C during the dark period. Exposure to temperatures above 28 °C, especially combined with long photoperiods, increases the likelihood of early flowering and bitterness due to increased sesquiterpene lactones. Lighting is user-defined electric load with scenario-specific photoperiod (10–16 h). Heat pump/HVAC: performance maps applied with heating/cooling setpoints 12–18 °C/20–28 °C. Deviations from these conditions can result in delayed growth, poor head formation, or early bolting. ^13^ No single temperature regime was defined as a fixed baseline for the 48 operational scenarios. Instead, temperature setpoints were selected to span a literature-informed control envelope for lettuce cultivation under vertical farming conditions. Heating setpoints ranged from 12 to 18 °C, while cooling thresholds ranged from 20 to 28 °C, covering both optimal and marginally suboptimal conditions reported in controlled-environment agriculture studies. This approach allowed the systematic evaluation of energy demand, grid dependency, and crop cycle behavior across realistic operational conditions, rather than benchmarking against a single assumed “optimal” temperature. The selected temperature ranges are consistent with reported optimal growth conditions for lettuce (18–24 °C during the light period) and documented tolerance limits below 14 °C and above 28 °C, beyond which growth delays or physiological stress may occur.

**Table 2.** Literature review on lettuce cultivation conditions.

Photoperiod	Humidity	Temperature	Duration	Cultivation type	CO _2_	Ref.
16	60–80%	16–18 °C	transplanted 20 + 35 days	Vertical Farming (VF)		14
16–20	65%	22 °C	14 + 30	Indoor Controlled Environment Agriculture (CEA)		15
24		23,9	30 days	Hydroponic Yield		16
10	60–80	20,5–24	15 + 30	Tempered glass covered greenhouse (GH)		17
12–16	60–65%	21.7	29 + 48	Conventional soilbased cultivation (CN) system	11.97 m ^−2^ s ^−1^	18
12–16	60–65%	22.3	22 + 40.5	Deep water culture (DWC) system	12.95 m ^−2^ s ^−1^	
12–16	60–65%	23.3	22 + 47	Nutrient film technique (NFT) system	10.18 m ^−2^ s ^−1^	
12		20–22	35			19
18		22–28		Directly from the Seeds in Three Separate NTF Hydroponic Systems		20
16–20	70–80	22–26/15–20	30 days			21
	65–75	24/18	19 days after transplantation			22
	60	11	34			23
	60	20	15			
	60	32	14			
	60–70	17–22	57 days			24

Each cultivation cycle begins with seed germination in nursery trays containing cocopeat or perlite for 10 days under constant 50–70% relative humidity. Dehumidification was modeled implicitly as part of the HVAC operation through latent load removal in the single-zone Type 56 model. A relative humidity control band was defined for the indoor air, and when the upper humidity threshold was exceeded, dehumidification was activated via the cooling system by removing latent heat from the air. No standalone dehumidifier component was modeled; instead, moisture control was achieved through the HVAC system’s cooling and ventilation operation, consistent with typical enclosed vertical farming installations. The associated energy demand of dehumidification was therefore accounted for within the cooling load of the heat pump, as calculated by TRNSYS based on zone moisture balance and latent loads. Relative humidity control was implemented using a common upper-limit control strategy applied consistently across all scenarios. An indoor relative humidity control band of approximately 50–70% was defined, and dehumidification was activated when the upper threshold was exceeded. Scenario-specific humidity conditions were allowed to evolve within this control framework, enabling the assessment of their impacts on energy demand and crop cycle duration. Following transplanting, crops were grown under controlled conditions in a vertical rack system with recirculating hydroponics. Two substrates were evaluated: (i) mineral wool (rockwool), and (ii) expanded clay pellets. The rockwool system exhibited superior water-holding capacity and capillarity, supporting maximum yields of 5.26 kg/m ^2^ per cycle, compared to 3.82 kg/m ^2^ in the clay pellet system. The nutrient solution was based on a modified Hoagland formulation with continuous pH and EC monitoring. The pH was maintained at 5.5–6.0, while electrical conductivity ranged from 1.8 to 2.2 mS/cm, adjusted according to growth stage. The solution was recirculated at a flow rate of 1.2 − 1.5 L/min, ensuring full saturation and oxygenation. Water temperature was kept at 18–20 °C to minimize thermal shock and root stress. No artificial CO _2_ enrichment was applied. Instead, air exchange was passively regulated to maintain ambient CO _2_ concentrations (~400–450 ppm). Relative humidity was controlled at 50–70%, with automated misting used during the seedling stage. The vertical racks consisted of 4 cultivation tiers spaced 50 cm apart, with LED luminaires delivering variable PPFD levels (200–350 μmol/m ^2^/s) depending on the lighting scenario. Harvest took place at physiological maturity, corresponding to an average fresh weight of approximately 120–150 g per head, based on pilot-scale operational data from the studied vertical farming unit and consistent with values commonly reported in the literature ( Table 2) for lettuce cultivated under controlled-environment conditions, depending on lighting strategy and environmental control conditions.

### 3.2 System setup

**Figure 3.**
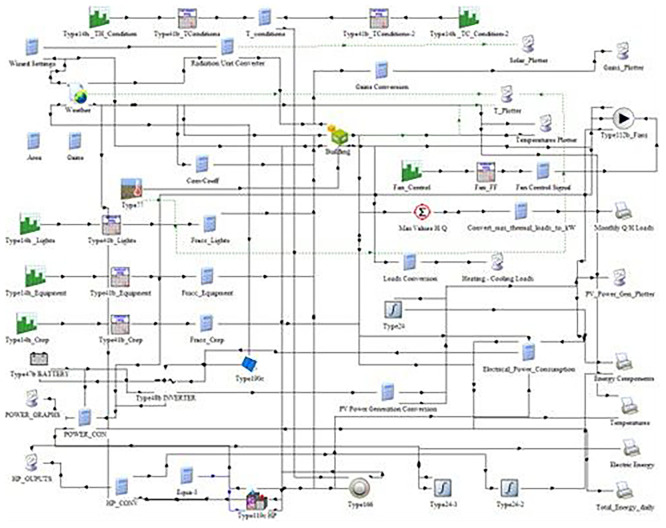
Configuration of the TRNSYS 18 model for the vertical farming unit. The schematic integrates weather inputs, lighting, HVAC, PV generation, battery storage.

Figure 3 illustrates the structure of the simulation model, including components for weather input, lighting, HVAC, photovoltaic power generation, battery storage, and crop energy demand. The model enables the calculation of total energy consumption and PV contribution under different cultivation scenarios. This flowchart was exported from TRNSYS 18 Studio. Within this environment, a full-year simulation was developed to capture quarter hourly fluctuations in energy demand and supply for the pilot VF system. Simulations replicate the structural and operational characteristics of the existing hydroponic vertical farm system operating under the COALITION project. The HVACD system was modeled, along with climate database taking from METEONORM for the case of Northern Greece.

All the energy components of the VF system, including lighting, heating, cooling, and dehumidification, were simulated in an integrated manner within TRNSYS 18 to assess seasonal performance. PV generation was also modeled matching the actual specifications of the installed VF system, which is integrated with inverter and batteries. The battery storage system was modeled as a 24 V battery bank, composed of series-connected 2 V cells. The 2 V value reported in Table 1 refers to the nominal voltage of individual battery cells, while the overall system operates at 24 V, consistent with typical small-scale PV installations. The energy systems were incorporated to simulate load shifting and storage of surplus PV energy production, while the MPPT inverter regulates energy from PV to storage, to consumption for the components that require energy and to the energy grid for surplus energy production. Thermal energy gains of the VF system derive from crop cultivation of lettuce in terms of latent heat throughout their evapotranspiration. Latent heat gains resulting from plant evapotranspiration were estimated by quantifying the mass of water transpired by lettuce plants and applying the latent heat of vaporization. Latent heat gains within the vertical farming unit were primarily attributed to plant transpiration during lettuce cultivation. Transpiration rates were quantified based on literature values reported for controlled-environment lettuce production, ranging between 0.2 and 0.5 L per plant per day, depending on lighting intensity, temperature, and relative humidity. The total latent heat load was calculated by converting the transpired water mass into latent heat using the latent heat of vaporization of water (h_fg ≈ 2450 kJ/kg at 20 °C). This load was implemented in TRNSYS Type 56 as an internal latent gain, allowing the HVACD system to dynamically respond through dehumidification. The NFT system was not treated as an independent humidity source, as moisture release originates from plant transpiration rather than the nutrient solution itself. The process accounts for the energy required to convert liquid water released by the plants into water vapor, which contributes to the internal latent load of the space. The total latent heat gain (Q _latent_) was calculated using the equation:

$$Q_{latent}=m\cdot h_{fg}$$ (1)

where *m* is the mass flow rate of transpired water (kg/day or kg/h), and h *_fg_* is the latent heat of vaporization of water which is approximately 2,450 kJ/kg, at 20 °C under standard atmospheric pressure. Transpiration rate was derived from literature values specific to indoor vertical farming conditions for lettuce, taking into account light intensity, temperature, and relative humidity. A value in the range of 0.2–0.5 l/day per plant was used to reflect typical water loss through transpiration under controlled environment agriculture (CEA). ^25^^,^^26^ Sensible heat gains concern the electrical equipment of the VF system including fans, pumps, and the lighting system. All electrical power consumed by the LED lighting system was assumed to be fully converted into internal sensible heat and explicitly included in the zone energy balance of the single-zone Type 56 model, directly affecting the heating and cooling loads calculated by the HVAC system. The model incorporates dynamic interactions between internal loads (e.g. lighting, plant evapotranspiration, electrical equipment, etc.), external climate conditions, and HVACD system. Indoor setpoints for each scenario (heating, cooling, humidity, lighting duration) were introduced as control parameters, dynamically adjusted to reflect realistic operating logic for lettuce cultivation. Humidification and dehumidification were accounted for by balancing vapor pressures based on the indoor air setpoints and crop transpiration rates.

### 3.3 Scenario definition and crop cycles

To explore the dynamic behavior of energy consumption in vertical farming, a number of cultivation scenarios were conducted, each representing a specific combination of environmental conditions relevant to lettuce growth in terms of temperature set-point and humidity levels. The indoor environmental conditions were tailored for safe lettuce growth. Lighting requirements were defined across various scenarios, reflecting the photoperiods. Thus, three key environmental parameters were systematically varied across scenarios ( Table 3):

• Relative humidity, ranging from 50% to 70%,
• Photoperiod duration, from 10 to 16 hours per day, and
• Setpoint temperatures, with heating temperatures from 12 °C - 18 °C and cooling thresholds from 20 °C - 28 °C.

**Table 3.** Multiple Linear Regression Model.

ANOVA Table (Crop Cycle ~ ΔT + Photoperiod + Humidity)					
Source	DF	Sum of Squares	Mean Square	F-value	P-value
**ΔT**	1	533.94	533.94	3879.33	<0.0001
**Photoperiod**	1	540	540	3923.33	<0.0001
**Humidity**	1	0	0	0	0.998
**Residual**	44	6.06	0.14		

Model Summary					
Statistic	Value				
R-squared (R ^2^)	0.994				
Adjusted R-squared	0.994				
F-statistic	2601				
Prob (F-statistic)	1.59E-49				
Observations	48				
Degrees of Freedom	44 (residual)				

Coefficient Table					
Term	Coefficient	Std. Error	t-value	P-value	Interpretation
Intercept	57.02	0.512	111.3	<0.001	Base duration with zero inputs
ΔT	0.645	0.01	62.3	<0.001	Longer cycles with wider temperature gap
Photoperiod	–1.5	0.024	−62.6	<0.001	Shorter cycles with longer light periods
Humidity	~0.0001	0.007	≈ 0	0.998	No significant effect on crop cycle duration

Each one of the scenarios represents a distinct cultivation schedule, with unique crop cycle durations and annual repetition frequencies. In order to find the required crop cycle for each scenario values were taken from literature overview as presented in Table 2 concerning the lettuce cultivation conditions as well as data taken from the operation of the installed VF system. Thus, a multiple linear regression model was developed to estimate crop cycle duration as a function of environmental control parameters. Using 48 scenario configurations, the model considered temperature differential (Δ *T*), photoperiod, and humidity as predictive variables.

$$\text{y}={\text{β}}_{0}+{\text{β}}_{1}{\text{x}}_{1}+{\text{β}}_{2}{\text{x}}_{2}+\text{⋅}\cdot \cdot .+{\text{β}}_{\text{p}}{\text{x}}_{\text{p}}+\text{ε}$$ (2)

Where:

• y: Dependent variable (response)
• β _0_: Intercept (value of y when all x _i_ = 0)
• β _1,_ β _2_, … …,β _p_: Regression coefficients
• x _1_, x _2_…, x _p_: Independent variables (predictors)
• ε: Error term (residual)

Thus, the final fitted model used in all scenario computations was:

CropCycle(days)=57.02+0.645⋅ΔT–1.50⋅Photoperiod+0.0001⋅Humidity (3)

with R ^2^ = 0.994, Adj-R ^2^ = 0.994, and the humidity term non-significant (p ≈ 0.998).

It should be noted that Equation (3) is valid only within the controlled lighting conditions examined in this study, corresponding to photosynthetic photon flux density (PPFD) levels in the range of 200–350 μmol·m ^−2^·s ^−1^. Extrapolation beyond this PPFD range is outside the scope of the present analysis.

**Figure 4.**
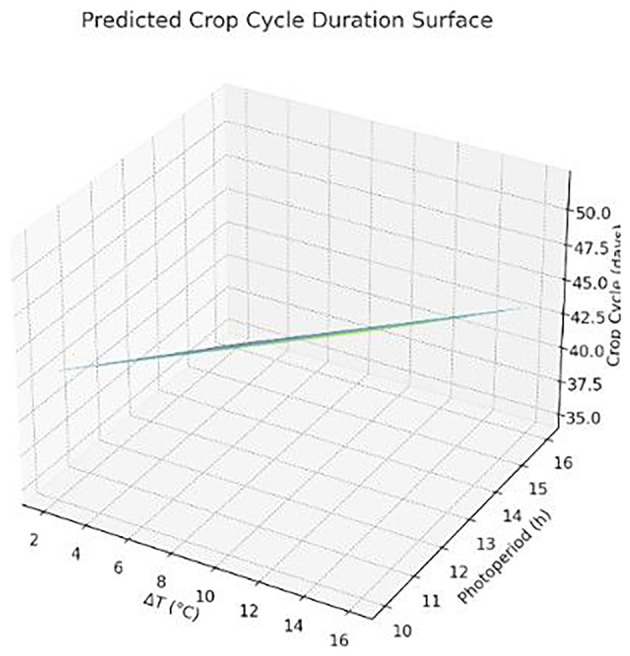
Predicted crop cycle duration as a function of photoperiod and temperature difference (ΔT). Longer light periods reduce cycle length, while wider ΔT slightly increases it.

The following Figure ( Figure 4) presents a 3D surface plot visualizing the Final Crop Cycle Estimation Model, which shows that crop cycles decrease significantly with increasing photoperiods, while they increase modestly with higher ΔT (temperature difference). This confirms the dominant role of light duration in accelerating growth, with ΔT offering a secondary, supportive influence.

The 3D surface represents the modeled crop cycle duration (in days) as a function of photoperiod (h/day) and temperature difference (ΔT in °C). Results were derived using a multiple linear regression model applied to simulation outputs. Graph produced using AI-assisted plotting tools.

Thus, the scenarios simulate a distinct cultivation strategy that leads to a different crop cycle duration, varying between 34 and 52 days as presented in Table 4. Shorter cycles (34–37 days) were observed in configurations combining high photoperiods (16 h), moderate humidity (50–60%), and elevated temperatures (18 °C heating, 20 °C cooling). Conversely, longer cycles (49–52 days) appeared in scenarios with lower light exposure (10 h), minimal heating (12 °C), and broader temperature differentials between day and night. This structured variation allows for assessing how tighter climate control and intensified lighting can accelerate plant development, thereby increasing the number of annual cycles, while also evaluating the trade-offs in energy demand and sustainability. TRNSYS 18 simulations produced energy demand profiles for lighting and HVACD, which were subsequently compared against simulated PV energy availability on a quarter hourly basis. The aim was to quantify PV sufficiency across all scenarios and to assess energy demands.

**Table 4.** Environmental parameter combinations across the 48 lettuce cultivation scenarios, with estimated crop cycle durations.

Scenario	Heating Temperature (°C)	Cooling Temperature (°C)	Humidity (%)	Photoperiod (hours)	Estimated Crop Cycle (days)
s1	12	28	50	10	52
s2	12	28	50	12	49
s3	12	28	50	14	46
s4	12	28	50	16	43
s5	12	28	60	10	52
s6	12	28	60	12	49
s7	12	28	60	14	46
s8	12	28	60	16	43
s9	12	28	70	10	52
s10	12	28	70	12	49
s11	12	28	70	14	46
s12	12	28	70	16	43
s13	14	24	50	10	49
s14	14	24	50	12	46
s15	14	24	50	14	43
s16	14	24	50	16	40
s17	14	24	60	10	49
s18	14	24	60	12	46
s19	14	24	60	14	43
s20	14	24	60	16	40
s21	14	24	70	10	49
s22	14	24	70	12	46
s23	14	24	70	14	43
s24	14	24	70	16	40
s25	16	22	50	10	46
s26	16	22	50	12	43
s27	16	22	50	14	40
s28	16	22	50	16	37
s29	16	22	60	10	46
s30	16	22	60	12	43
s31	16	22	60	14	40
s32	16	22	60	16	37
s33	16	22	70	10	46
s34	16	22	70	12	43
s35	16	22	70	14	40
s36	16	22	70	16	37
s37	18	20	50	10	43
s38	18	20	50	12	40
s39	18	20	50	14	37
s40	18	20	50	16	34
s41	18	20	60	10	43
s42	18	20	60	12	40
s43	18	20	60	14	37
s44	18	20	60	16	34
s45	18	20	70	10	43
s46	18	20	70	12	40
s47	18	20	70	14	37
s48	18	20	70	16	34

### 3.4 Simulation process

Quarter-hourly dynamic simulations were performed in TRNSYS 18 over a full annual period (8,760 h) for each of the 48 scenarios. The model generated time-series outputs including: (i) indoor operative and ambient temperatures, (ii) total and beam solar radiation, (iii) HVAC heating and cooling loads, (iv) photovoltaic (PV) generation, (v) grid import/export flows, (vi) battery charge–discharge cycles, and (vii) auxiliary electrical consumption. These outputs formed the basis for the subsequent energy balance, resilience, and carbon footprint analyses, the results of which are presented and discussed in Section 4.

### 3.5 Resilience Indicators and Scenario Classification

To assess the adaptability of each vertical farming scenario under fluctuating solar and thermal conditions, two composite performance indicators were formulated based on the annual and monthly energy balance outputs derived from the TRNSYS simulations. These indices were specifically developed within the framework of the present study to quantify grid independence and seasonal robustness of photovoltaic (PV) integration. The first indicator, the Grid Independence Index (GII), captures the proportion of total annual energy demand that is covered by on-site PV generation and battery storage, thus reflecting the overall degree of energy autonomy. The second indicator, the Seasonal Resilience Score (SRS), was introduced to quantify intra-annual variability in PV contribution and expresses the system’s ability to maintain PV sufficiency during its lowest-performing month relative to its average monthly PV coverage. These proposed indicators do not correspond to previously published formulations but are derived directly from fundamental energy balance relationships and were designed to enable comparative scenario-level resilience analysis within this work. Together, GII and SRS allow simultaneous evaluation of both structural energy autonomy and temporal robustness under seasonal solar variability.

$$\text{GII}=\left(1-\frac{\text{Annual}\;\mathrm{Grid}\;\text{Energy}\;\text{Use}}{\text{Total}\;\text{Annual}\;\text{Energy}\;\text{Demand}}\right)\times 100$$ (4)

$$\text{SRS}=\frac{\text{PV}\;\text{Coverage}\;\text{in}\;\text{Lowest}\;\text{Month}}{\text{Average}\;\text{Monthly}\;\text{PV}\;\text{Coverage}}$$ (5)

$$\text{PV}\;\text{Coverage}=\frac{\text{PV}\;\text{Used}}{\text{Monthly}\;\text{Energy}\;\text{Demand}}$$ (6)

To further assess the robustness of each scenario under energy-constrained conditions, the resilience score of all scenarios was evaluated based on two dimensions: the worst-case PV deficit across a full crop cycle and the seasonal variability in PV availability. Four descriptive energy-performance typologies were set:

• **High Demand, Optimizable:** Heavy energy use but good potential for savings via cycle shifts or dynamic scheduling.
• **High Demand, Rigid:** High loads with limited flexibility, likely candidates for backup systems or setpoint reduction.
• **Moderate, Responsive:** Mid-range energy demand with high responsiveness, efficient and adaptable setups.
• **Stable, Low-Demand:** Low energy needs and minimal gains from optimization, ideal for low-intervention farming.

The calculated indices were subsequently used to generate comparative plots and scenario classifications, which are presented and discussed in the Results section.

### 3.6 Multivariate Scenario Classification

Principal Component Analysis (PCA) was applied to six standardized performance indicators derived from dynamic crop-cycle simulations (maximum HVAC demand, minimum PV availability, total cycle energy, optimized energy, energy savings, crop duration). K-means clustering (k = 4) was subsequently applied in the reduced PCA space to identify scenario groupings. The PCA-derived clusters align with the resilience typology defined in Section 3.5.

### 3.7 CO _2_ Emissions Estimation

For each scenario (S1–S48), monthly simulation outputs were aggregated to derive annual system energy requirements. Electricity supply was modeled as a hybrid system combining on-site PV generation and grid electricity. Grid electricity consumption was derived directly from the quarter-hourly TRNSYS energy-flow outputs and corresponds to the fraction of electrical demand not covered by on-site photovoltaic (PV) generation and locally stored energy. A self-consumption operating scheme was assumed, whereby PV electricity was first allocated to on-site loads, and any surplus was stored in the battery or curtailed; no feed-in to the grid was considered. Battery degradation effects were not included, as the analysis focuses on annual operational energy flows and associated CO _2_ emissions. An emission factor of 0.256 kg CO _2_/kWh was applied to grid-supplied electricity, based on the Greek National Inventory and the country-average electricity mix. ^27^ A life cycle assessment (LCA) of greenhouse gas emissions (GWP) was conducted per kilogram of harvested fresh lettuce (functional unit: 1 kg; average head weight 250 g). The system boundaries include operational electricity use (LED lighting, HVACD, water pumping/recirculation, valves, and monitoring/sensor systems) and cultivation-related inputs associated with a full crop cycle (e.g., substrates such as rockwool and clay pellets, growth media, and total water consumption). The life cycle inventory was established using real-time monitoring data from the pilot unit (e.g., water use, grid electricity), complemented with literature-based assumptions (e.g., germination duration). The resulting scenario-specific electricity uses and carbon footprint values are reported and discussed in Section 4.3 ( Table 5).

**Table 5.** CO _2_ Emissions for each scenario based on LCA.

CO _2_ Emissions for Each Scenario based on LCA					
Scenarios	Total Electricity (kWh)	Total CO _2_ (kgCO _2_eq/kg lettuce)	Scenarios	Total Electricity (kWh)	Total CO _2_ (kgCO _2_eq/kg lettuce)
S1	2455.3	2.3	S25	3839.2	3.5
S2	3198.1	2.9	S26	4587.9	3.6
S3	3908.7	3.5	S27	5242.2	3.7
S4	4659.2	3.7	S28	5264.5	3.7
S5	2460.8	2.3	S29	3858.3	3.5
S6	3210.9	2.9	S30	4607.8	3.7
S7	3921.5	3.6	S31	5262.3	3.7
S8	4674.4	3.7	S32	5980.3	4.2
S9	2472.0	2.3	S33	3876.8	3.5
S10	3217.3	2.9	S34	4614.0	3.7
S11	3940.4	3.6	S35	5280.3	3.7
S12	4693.6	3.7	S36	6002.2	4.2
S13	3068.3	2.8	S37	4971.5	3.9
S14	3808.0	3.5	S38	5712.7	4.0
S15	4486.4	3.6	S39	6346.8	4.4
S16	5216.8	3.7	S40	7061.0	4.4
S17	3082.7	2.8	S41	4989.3	3.9
S18	3816.3	3.5	S42	5722.6	4.0
S19	4504.1	3.6	S43	6363.5	4.5
S20	5242.2	3.7	S44	7086.3	4.5
S21	3093.6	2.8	S45	5015.2	4.0
S22	3829.3	3.5	S46	5740.7	4.0
S23	4523.1	3.6	S47	6383.9	4.5
S24	5264.5	3.7	S48	7092.5	4.5

### 3.8 Dynamic Lighting Control on Energy and Crop Cycle Duration

To enhance PV self-utilization and minimize grid dependency, a dynamic lighting strategy is implemented that selectively reduces artificial lighting during low-irradiance (“cloudy”) days. Cloudy days were identified by applying a threshold-based condition on daily PV generation data. Specifically, days with production values below the 10th percentile of the annual PV generation profile or more than one standard deviation below the seasonal average were flagged as “cloudy”. This method served as a proxy for reduced solar availability in the absence of direct irradiance data. On such days, lighting energy demand was proportionally reduced by 2.5%, 5%, 7.5% and 10%, resulting trade-offs in crop cycle duration were estimated using a regression model linking photoperiod to crop growth rate. The dynamic model ensures lighting reduction is only triggered when PV generation is insufficient, thereby reducing grid load without severely compromising crop timelines. Dynamic lighting reductions were assumed to influence the rate of crop development and cultivation time, while final harvest mass per cycle was preserved by extending the growing period until physiological maturity. This adaptive strategy offers a realistic and implementable approach to load shifting in vertical farming systems, especially where PV-battery systems are deployed. To support adaptive energy and schedule optimization in vertical farming systems, this work developed a predictive model for estimating crop cycle duration as a function of key environmental control parameters as discussed in Section 3.3. This model can be subsequently used to estimate the impact of dynamic lighting reductions on crop timing under various scenarios. Using the quarter-hour PV generation profile, days with significantly lower PV output compared to the seasonal average were tagged as cloudy or low-solar days. A rolling average threshold (bottom 10–15% of daily PV generation) was applied across the year to isolate these days. Once low-PV days were identified, reducing LED lighting load by 2.5%, 5%, 7.5% and 10%, was proposed, but only on those specific days. This was applied selectively, avoiding days with sufficient PV, to minimize system grid draw. Thus, for

***Cloudy Day Detection Condition***

$P_{i}:\text{Daily}\;\mathrm{PV}\;\text{energy production}\;\mathrm{on}\;\mathrm{day}\;i$

$\mu_{P}:\text{Rolling or seasonal average}\;\mathrm{PV}\;\text{energy production}$

$\sigma_{P}:\text{Standard deviation of daily}\;\mathrm{PV}\;\text{generation}$

${Τ}_{P}:\mathrm{PV}\;\text{production threshold for cloudy days}\;\left(e.g.,\text{bottom}\;10-15\%\right)$

A day is considered “cloudy” if:

$$P_{i}<\mu_{P}-a\cdot \sigma_{P}\;\text{or}\;P_{i}<T_{P}$$ (7)

Where:

a∈[1,1.5]:sensitivity coefficient

$T_{P}:\text{empirically}\;\mathrm{set}\;\text{percentile cutoff}\;\left(e.g.,10\mathrm{th}\;\text{percentile of annual}\;\mathrm{PV}\;\text{data}\right).$

***Dynamic Lighting Reduction Application***

L:Baseline lighting energy(kWh/day)

δ∈{0.025,0.05,0.075,0.1}:lighting reduction factor

$L΄=L∙\left(1-\delta \right):\text{adjusted lighting consumption}\;{\mathrm{on}}^{"}{\text{cloudy}}^{"}\;\mathrm{day}$

C:Baseline crop cycle duration(days)

C΄=C+f(δ):adjusted cycle durationviaregression model (8)

The extended cycle duration C΄ is estimated using a linear model:

C΄=a∙δ+b (9)

Derived from the photoperiod vs. crop duration model

By implementing the aforementioned formulas and by adjusting the new photoperiods as follows:

$${\text{Photoperiod}}_{\delta }={\text{Photoperiod}}_{0}∙\left(1-\delta \right)$$ (10)

A new cycle length duration is recalculated based on the regression model with the new ${\text{Photoperiod}}_{\delta }$ and for all the scenarios the energy difference between the initial values for energy consumption and the values for energy consumption after the dynamic lighting control were calculated. The new cycle length durations for each scenario are presented in Table 6.

## 4. Results and discussion

### 4.1 Annual energy demand & grid dependency per scenario

**Figure 5.**
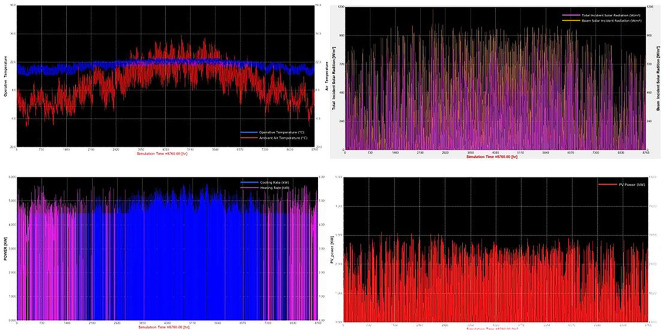
TRNSYS 18 Simulation Results for Specific Scenario. (a) Operative and ambient air temperature. (b) Total and beam incident solar radiation. (c) Heating and cooling loads. (d) Photovoltaic power output.

***4.1.1 Representative dynamic annual profiles.*** To illustrate the intra-annual dynamics of the simulated system, Figure 5 presents the annual time series for a representative intensive scenario (S40). Indoor operative temperature remained within the prescribed setpoint range (18–22 °C) despite significant outdoor fluctuations, confirming effective HVAC control ( Figure 5a). Seasonal variability in solar radiation ( Figure 5b) translated directly into PV production patterns ( Figure 5d), with marked reductions during winter months. Heating loads peaked during winter, while cooling demand increased in summer ( Figure 5c), reflecting the seasonal inversion of thermal requirements. These dynamics demonstrate the strong coupling between environmental control, renewable availability, and grid reliance, providing the foundation for the comparative scenario analyses that follow.

**Table 6.** Lighting Control Strategies Result.

Lighting Control Strategies Results				
Scenario	Max Safe Lighting Reduction (%)	Rounded Crop Cycle (days)	Estimated Energy Use (kWh)	Energy Saved (kWh)
S1	10	54	857.8	22.0
S10	10	51	902.5	25.1
S11	10	48	929.2	31.4
S12	7.5	45	954.3	10.1
S13	10	50	915.8	9.8
S14	10	47	948.1	14.4
S15	7.5	44	942.3	5.6
S16	10	42	951.5	3.5
S17	10	50	916.3	8.8
S18	10	47	947.9	13.4
S19	7.5	44	941.3	4.7
S2	10	51	902.0	26.1
S20	10	42	951.5	3.5
S21	10	50	916.3	8.8
S22	10	47	944.9	14.1
S23	7.5	44	941.2	4.4
S24	10	42	949.4	2.3
S25	10	47	995.7	6.3
S26	7.5	44	994.6	1.2
S27	10	42	1000.9	0.2
S28	2.5	37	966.4	11.0
S29	10	47	994.7	7.4
S3	10	48	929.1	32.4
S30	7.5	44	994.3	1.2
S31	7.5	41	990.9	9.7
S32	2.5	37	965.2	11.7
S33	10	47	994.0	5.2
S34	7.5	44	991.5	0.8
S35	7.5	41	988.3	9.3
S36	2.5	37	964.5	11.5
S37	0	0	0.0	0.0
S38	0	0	0.0	0.0
S39	5	38	1058.3	0.4
S4	7.5	45	953.2	11.6
S40	0	0	0.0	0.0
S41	0	0	0.0	0.0
S42	0	0	0.0	0.0
S43	0	0	0.0	0.0
S44	0	0	0.0	0.0
S45	0	0	0.0	0.0
S46	0	0	0.0	0.0
S47	0	0	0.0	0.0
S48	0	0	0.0	0.0
S5	10	54	857.8	22.0
S6	10	51	902.5	25.1
S7	10	48	929.7	31.4
S8	7.5	45	953.6	10.8
S9	10	54	857.9	21.0

**Figure 6.**
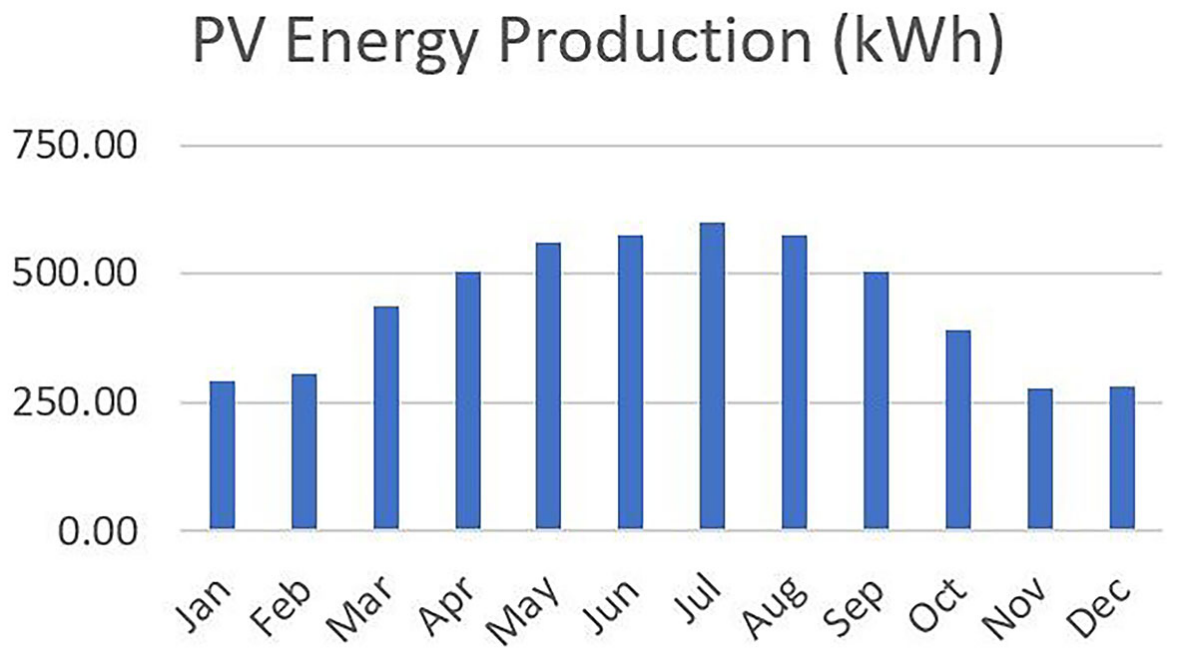
Monthly PV energy production profile for Northern Greece, with higher output in summer and lower in winter.

***4.1.2. Annual PV energy production & energy analysis.*** The PV integrated into the VF unit was modeled under fixed operational parameters based on the optimal tilt angle and orientation, for the case of Northern Greece. The system’s specifications remained the same across all 48 scenarios. Based on local solar irradiance data and TRNSYS 18 simulations, the annual electricity generation from the PV system was estimated at approximately 5,234 kWh, with monthly values ranging from 280 kWh in December to 600 kWh in July, following the typical seasonal pattern. This consistent energy profile served as the renewable input baseline across all scenarios. Any deviations in grid dependency, energy autonomy, or carbon footprint thus stem from the variation in environmental control settings (e.g., photoperiod, HVAC setpoints) rather than PV availability. Figure 6 illustrates the monthly distribution of PV energy production.

**Figure 7.**
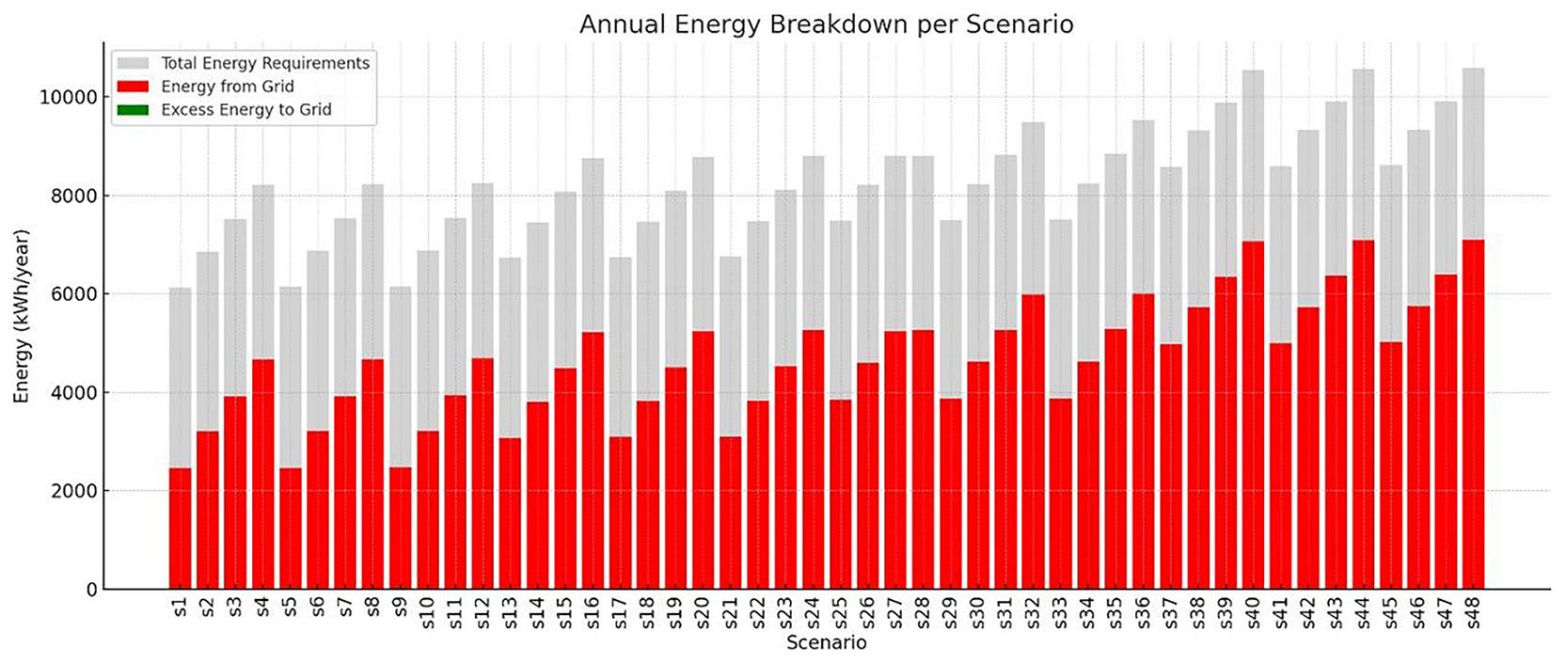
Breakdown of VF systems energy in annual basis.

The total annual energy requirements of the VF system vary considerably across the different scenarios, ranging from approximately 6,100 kWh to over 10,500 kWh ( Figure 7). Each stacked bar represents the total annual energy demand (gray), the portion covered by grid electricity (red), and any excess PV energy fed back into the grid (green), across 48 simulation scenarios. Diagram generated using Python-based visualization tools and refined using AI-assisted layout optimization. This variability reflects differences in environmental control strategies, such as photoperiod, HVAC setpoints and humidity levels which directly impact the system’s lighting and climate energy loads. The amount of energy required from the grid also shows substantial fluctuation, spanning from almost 2,500 kWh in the most efficient scenarios (e.g., S1 and S5) to nearly 7,100 kWh in the most demanding cases. These figures underscore the importance of scenario configuration in determining energy autonomy. Scenarios with optimized settings, such as moderate temperature differentials and daylight-aligned photoperiods, demonstrate a significantly lower dependency on external energy sources. While the PV system contributes to reducing this grid reliance, the excess energy exported back to the grid remains relatively minimal in all cases, typically under 1% of total generation. For example, in scenarios like S1 and S5, although a small surplus of around 20 kWh is sent back to the grid, the majority of PV energy is consumed internally by the system (energy requirements and batteries). This indicates that the system operates close to a self-consumption model, with high internal utilization of PV electricity and limited overproduction. Overall, this energy breakdown confirms that while PV integration significantly offsets the total energy demand, grid dependency is still substantial in high-demand scenarios. The data also reinforces the value of seasonal crop scheduling, dynamic lighting control, and HVAC optimization to reduce total energy use and enhance self-sufficiency. In systems where excess PV is minimal, energy storage and load-shifting strategies become essential for further reducing grid reliance and improving energy resilience.

**Figure 8.**
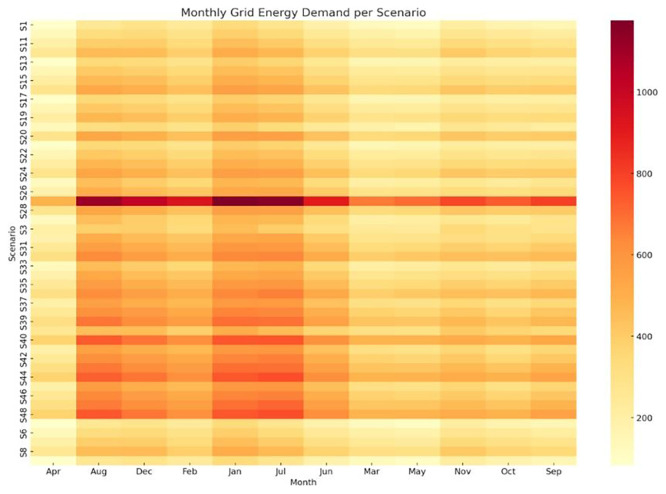
Monthly grid energy demand per scenario. Darker tones indicate higher dependency, with peaks in August, December, and January.

***4.1.3 Analysis of monthly grid energy demand by scenario.*** While electricity generation from PV remains fixed across all scenarios, the monthly grid energy demand varies significantly depending on the crop cycle configuration. These differences stem from diverse environmental control strategies, including heating and cooling setpoints, lighting durations, and humidity levels, all of which affect the total energy load and its seasonal distribution. To capture this variability, Figure 8 presents a heatmap of monthly grid electricity use for each scenario. The x-axis spans the calendar months, while the y-axis lists the 48 crop cycle scenarios sorted approximately by increasing annual energy consumption. This visualization reveals several key insights. Scenarios with higher energy demand exhibit intense grid dependency during months with large temperature fluctuations such as August, December and January reflected as deeper red tones on the heatmap. In contrast, energy-efficient scenarios, such as those with moderate environmental setpoints and shorter crop durations (e.g., S1–S10), display lighter tones, indicating lower grid dependency across the year. A notable asymmetry emerges in summer, where PV production peaks but energy demand may still rise sharply due to active cooling demands. This seasonal interplay underscores the importance of crop scheduling and environmental strategy optimization, particularly in months with low solar availability. Scenarios that compress crop cycles into spring and autumn months tend to benefit more from direct PV utilization.

Heatmap showing the variation in monthly grid energy requirements (in kWh) across all 48 simulation scenarios (S1–S48). Darker shades indicate higher dependency on grid electricity. Visualization generated using Python (matplotlib/seaborn); layout enhanced using AI-assisted tools.

**Figure 9.**
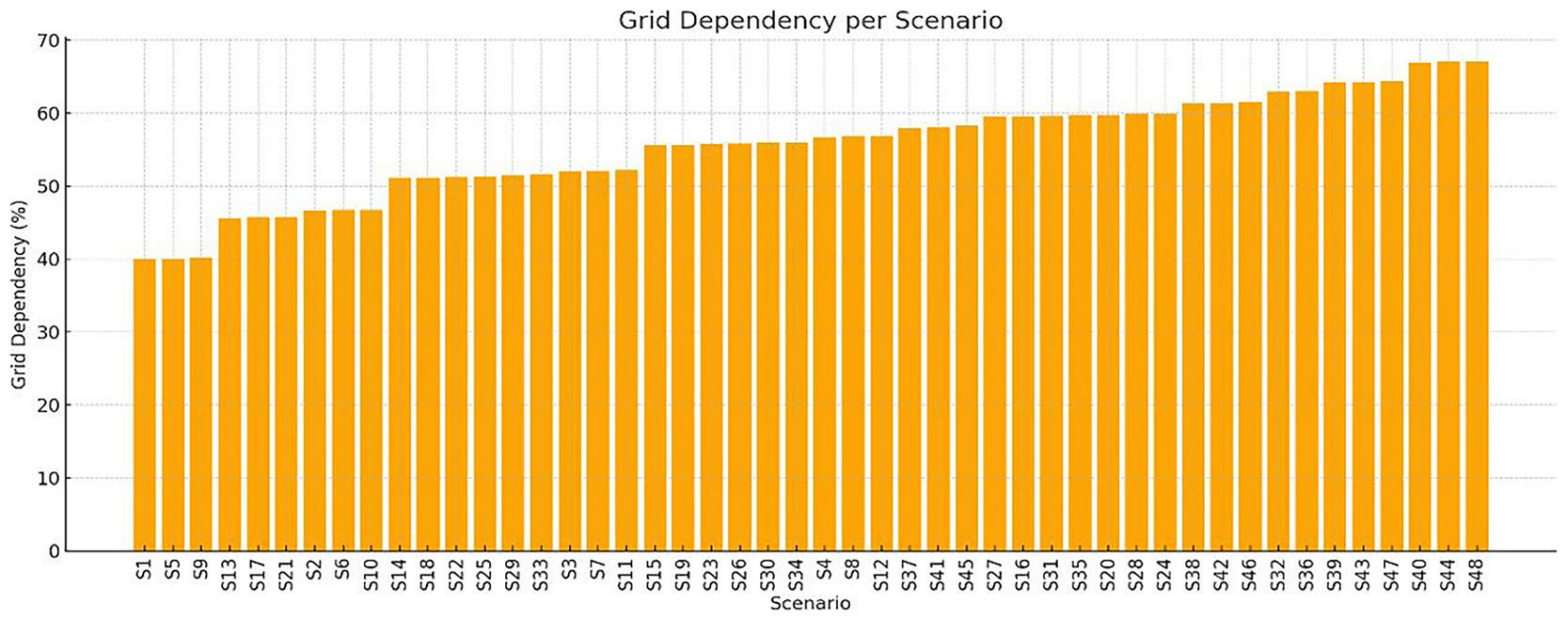
Grid dependency ratio for all 48 scenarios. Lower values (e.g., S1, S5, S9, S13) indicate higher PV contribution, while intensive scenarios (e.g., S44–S48) indicate higher grid reliance.

***4.1.4 Grid dependency per scenario.*** To better quantify the extent to which each scenario depends on grid for electricity supply, the Grid Dependency Ratio was computed for all 48 crop cycles. This metric reflects the percentage of the total annual energy demand that is met by the national grid, after accounting for the fixed annual PV contribution. Nearly all scenarios exhibit very high PV utilization (>99.6%), meaning almost all solar energy is consumed by the system. Since PV production is fixed for all scenarios, efficiency hinges on reducing demand or improving storage/load matching. Thus, the total energy demand varied significantly as a result of changing photoperiods, HVAC setpoints, and humidity levels. This variation in demand causes the share of PV energy to fluctuate accordingly, impacting grid reliance. The bar chart in Figure 9 illustrates the Grid Dependency Ratio values per scenario. Bar chart illustrating the percentage of grid dependency for each of the 48 simulation scenarios (S1–S48), based on annual energy balance results. Higher values reflect increased reliance on external electricity sources and reduced energy autonomy. Plot generated using Python’s matplotlib; layout support with AI-assisted enhancement. Scenarios such as S1, S5, S9, and S13 exhibit the lowest grid dependency (~40%), corresponding to milder temperature settings, shorter photoperiods, and moderate humidity levels. On the contrary, scenarios like S44, S47, and S48 demonstrate the highest dependency, exceeding 66%, primarily due to extended lighting durations and elevated heating setpoints. This visualization underscores the direct relationship between crop cycle intensity and grid reliance. Scenarios that aim for smaller crop cycles typically impose higher energy burdens, particularly in lighting and heat-pump use, thus diluting the relative impact of PV production. Conversely, scenarios with larger crop cycles align better with the PV output, requiring less energy from the grid.

### 4.2 Resilience analysis

**Figure 10.**
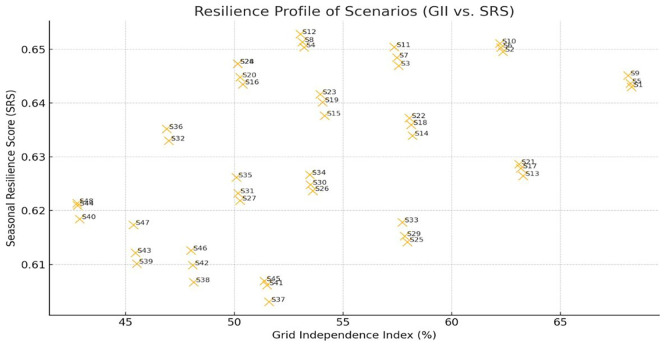
Resilience profile of scenarios based on GII and SRS. Upper-right cases are more resilient, while lower-left are grid-dependent.

The resilience map ( Figure 10) illustrates the distribution of scenarios in terms of grid independence (GII) and seasonal robustness (SRS). Scenarios located in the upper-right quadrant exhibit both strong annual PV coverage and stable seasonal performance, representing the most resilient configurations. Among them, S22 and S11 achieved high values for both indices. Scenario S1 recorded the highest GII (68.3%), while maintaining moderate seasonal robustness (SRS = 0.643). In contrast, scenarios such as S40 and S43 fall into the low-GII/low-SRS region, indicating both high grid dependence and seasonal vulnerability.

**Figure 11.**
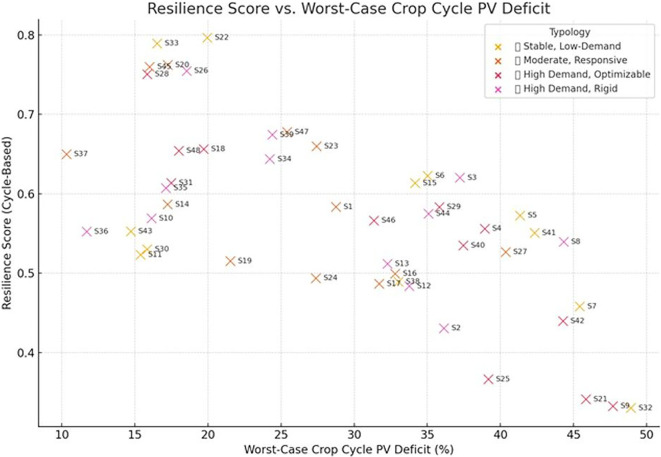
Resilience score versus worst-case PV deficit per crop cycle. Scenarios in the upper-right are more resilient, while those in the lower-left are vulnerable to energy shortages.

Figure 11 presents the cycle-based resilience assessment using worst-case PV deficit as an additional stress indicator. Scenarios S22, S33, and S26 demonstrate strong performance under both annual and cycle-based criteria, whereas S9, S21, and S32 exhibit higher PV deficits and reduced resilience. Integrating cycle-level stress analysis refines scenario benchmarking by revealing vulnerabilities that are not evident from annual averages alone.

When resilience indicators were combined with energy cost and carbon emissions in a composite multi-objective assessment, scenarios such as S33 and S22 consistently ranked among the most balanced configurations, achieving favorable trade-offs between sustainability and robustness. In contrast, some scenarios with strong sustainability performance (e.g., low cost and emissions) fared poorly in resilience, revealing potential hidden vulnerabilities under real-world energy dynamics.

***4.2.1 Optimal crop cycle and season placement.*** To better understand the interplay between energy efficiency and crop scheduling, a focused analysis was conducted on the minimum annual energy requirements associated with the most favorable crop cycle per scenario ( Table 7). These values derive from dynamic simulations incorporating TRNSYS 18 data and seasonal variations.

**Table 7.** Lowest Energy Demand Crop Cycles per Scenario.

Scenario	First Day of Cycle	Last Day of Cycle
s1	291	342
s2	295	343
s3	297	342
s4	306	348
s5	291	342
s6	294	242
s7	294	339
s8	306	348
s9	292	343
s10	294	342
s11	294	339
s12	306	348
s13	72	120
s14	286	331
s15	289	331
s16	292	331
s17	72	120
s18	286	331
s19	289	331
s20	292	331
s21	72	120
s22	286	331
s23	289	331
s24	292	331
s25	286	331
s26	289	331
s27	291	330
s28	295	331
s29	286	331
s30	289	331
s31	291	330
s32	295	331
s33	286	331
s34	289	331
s35	292	331
s36	295	331
s37	288	330
s38	291	330
s39	294	330
s40	297	330
s41	288	330
s42	291	330
s43	294	330
s44	297	330
s45	288	330
s46	291	300
s47	294	330
s48	297	330

**Figure 12.**
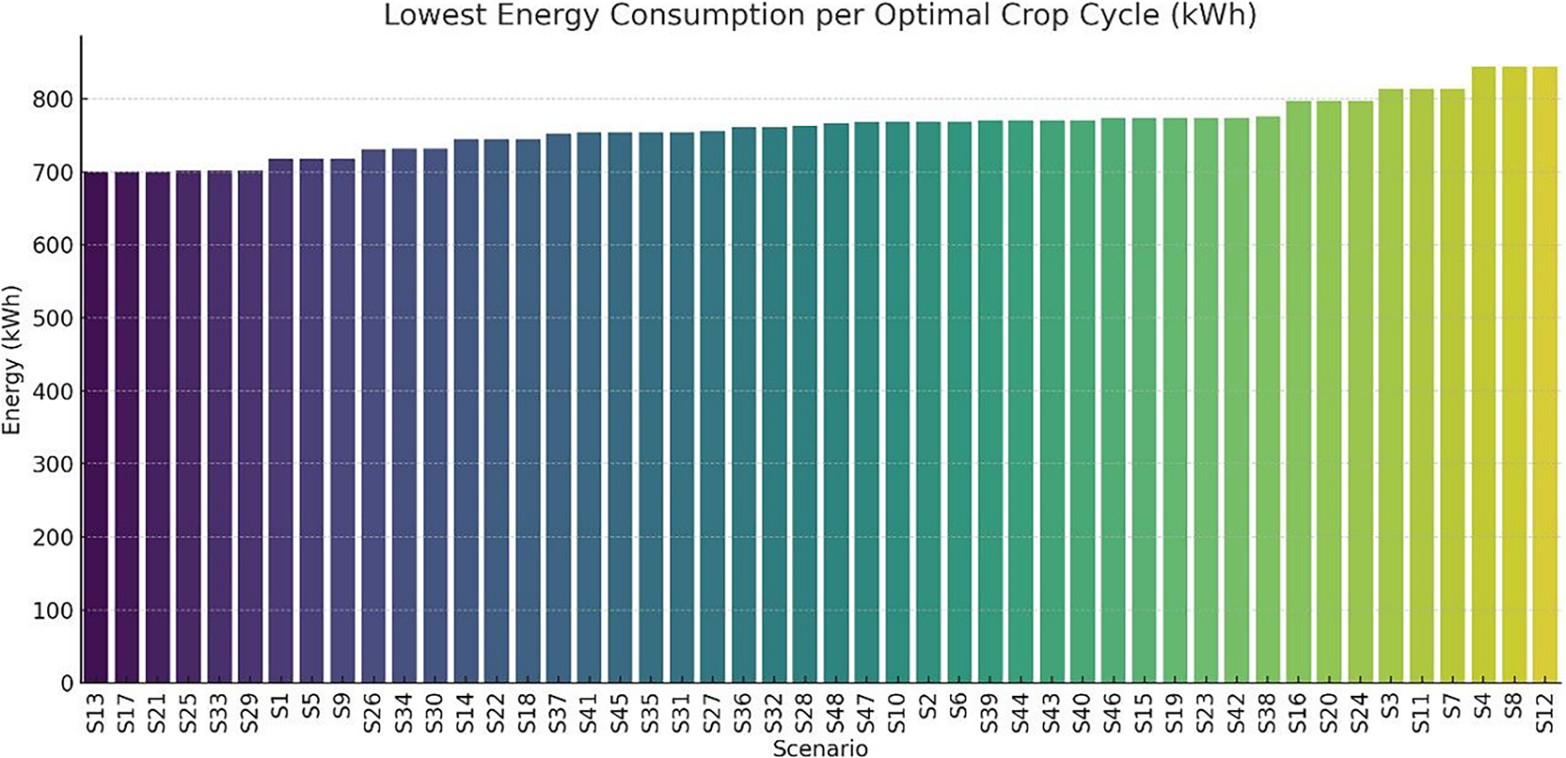
Optimal Crop Cycle Energy Profile example.

A typical depiction of s17 concerning the lowest energy demand for a full crop cycle, as well as its start and end dates during the year is presented in Figure 12. The bar graph shows daily energy consumption (kWh) throughout the year, with the optimal crop cycle window indicated in green. This period was identified through simulation as the most energy-efficient and resilient interval for cultivation under Scenario 17 conditions. Diagram generated using AI-assisted plotting tools in Python for enhanced visual clarity. For this case the optimal crop cycle takes place in spring time.

**Figure 13.**
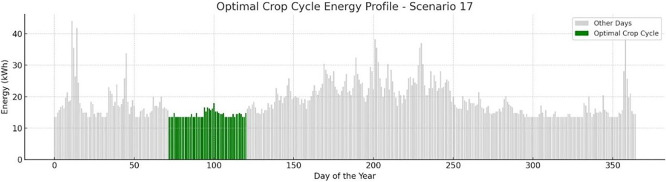
Optimal crop cycle energy demand across all scenarios.

A study was conducted on the optimal energy demand per scenario along with the corresponding first and last day of the crop cycle. The results are illustrated in Figure 13. The bar chart presents the total energy consumed (in kWh) during the most efficient cultivation cycle for each scenario (S1–S48). Scenarios are ranked from lowest to highest energy requirement, illustrating the variation in energy efficiency depending on environmental and operational parameters. Chart created using Python with AI-assisted data visualization techniques.

Scenarios such as S1, S13, S17, and S21 exhibit the lowest energy consumption (approximately 700–720 kWh), while other scenarios (e.g., S4, S12, S8) surpass 840 kWh. These variations are primarily driven by setpoint configurations (e.g., heating/cooling set temperatures) and photoperiod duration.

**Figure 14.**
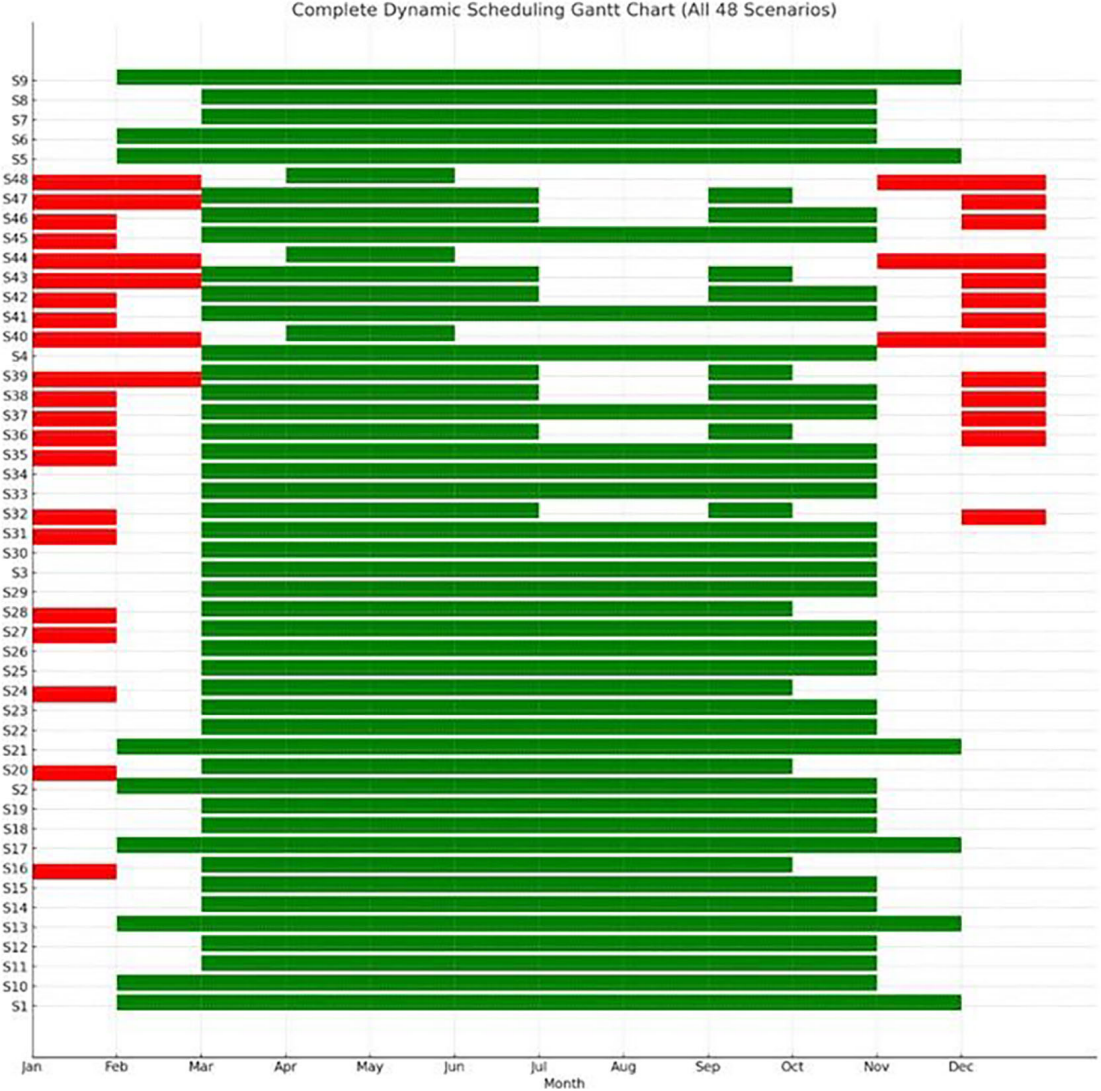
Dynamic scheduling Gantt chart of crop cycles across all scenarios.

To visualize how each crop cycle aligns with solar availability and ambient conditions, Figure 14 provides a dynamic Gantt chart of all scenarios, by providing information based on ascending energy requirements. Each row represents one of the 48 scenarios (S1–S48), while the green bars indicate the periods during which optimal crop cycles were scheduled. Red bars highlight energy-deficient periods that were excluded from scheduling due to low PV availability or high energy demand. This Gantt chart visualizes the adaptive temporal distribution of crop cycles throughout the year, according to energy sufficiency and environmental suitability. Chart produced using Python and AI-assisted scheduling analysis. Green bars indicate PV-favorable months (PV coverage >60%) which are ideal for starting crop cycles while red bars represent grid-stressed months (PV coverage <40%) where high-load operations are present. Blank months show a moderate energy balance where standard operations take place. Most high-performance scenarios (e.g., S1–S25) are clustered around the spring and autumn period, which offers favorable HVACD balance and improved PV yield. These scenarios avoid the extreme cooling load demands of summer and the high heating demands of winter. On the other hand, lower-ranked configurations like S4, S8, S12 are scheduled in late autumn or winter, where energy costs per cycle peak, and PV contribution is minimal. This temporal dimension of optimization demonstrates that seasonal timing is as critical as environmental control. Selecting appropriate crop windows allows the system to capitalize on periods of natural thermal neutrality and maximum PV availability, improving both energy independence and carbon footprint performance.

**Figure 15.**
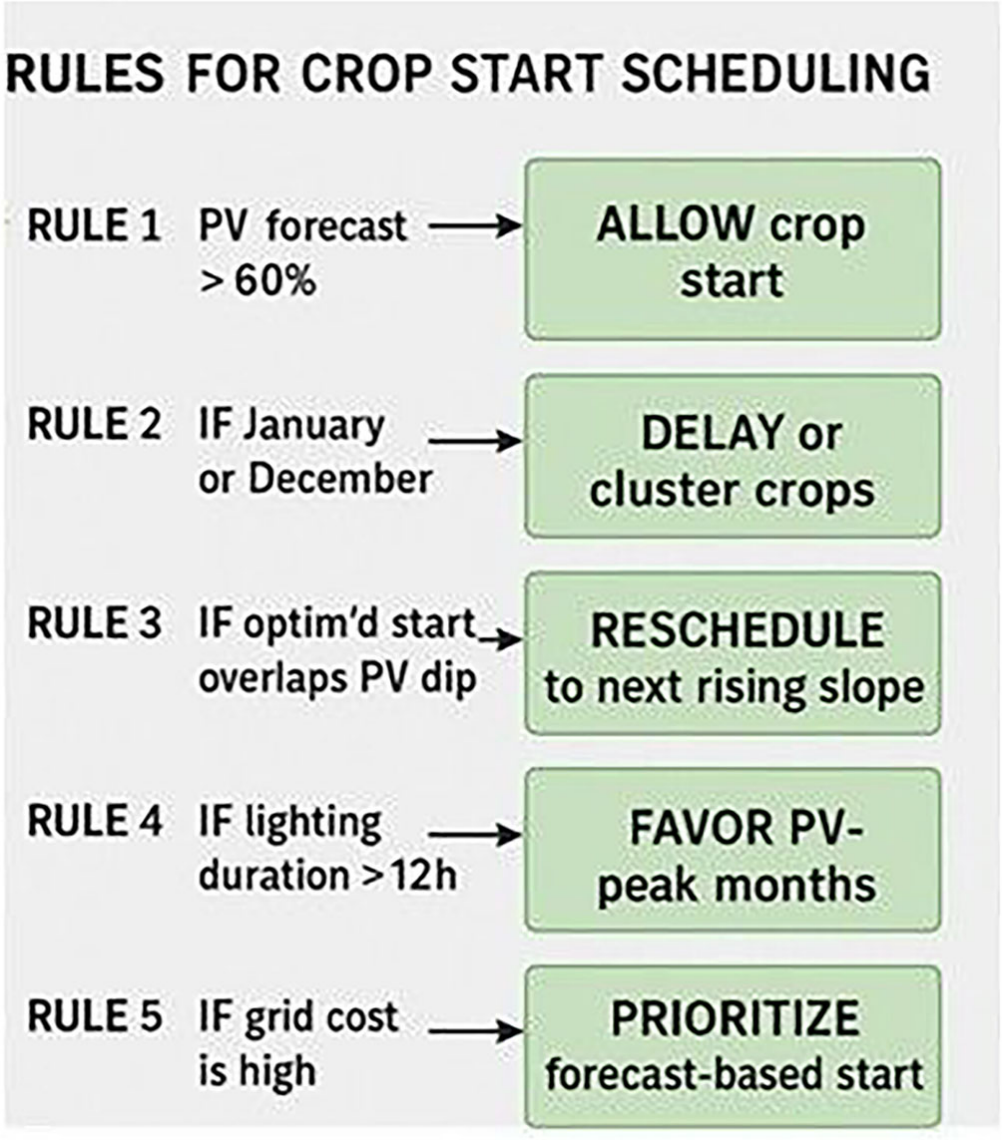
Rules for Crop Starting Scheduling.

To improve the energy efficiency of the VF system, one of the most impactful considerations is the timing of crop cycles. Aligning planting and harvesting schedules with months that are favorable for PV generation, typically March through May and September through October, can significantly reduce reliance on grid electricity. During these periods, solar irradiance tends to be higher, leading to more effective utilization of PV energy. Conversely, planting during winter months, such as December and January, should be avoided due to reduced solar availability and increased dependence on the grid. In regions like Greece, strategic scheduling can reduce grid energy consumption by over 40 percent. Employing a calendar-based Gantt model to plan crop cycles around these optimal solar periods ensures that energy use is aligned with the natural availability of renewable resources. In addition to scheduling, the climate control parameters of the indoor environment play a critical role in managing energy demand. Efficient setpoints for heating, ventilation, and air conditioning (HVAC) systems are essential. Cooling temperatures should be maintained between near 28 °C to reduce the energy load on heat pumps for lettuce cultivation. Heating, on the other hand, should be limited to a range of 12 °C up to 16 °C to minimize energy consumption during colder months. Maintaining a relative humidity of 50% to 60% percent strikes a balance between optimizing plant transpiration and limiting energy-intensive dehumidification or humidification processes. It is also important to avoid extreme internal temperature gradients. Differences greater than 18 °C between daytime and nighttime settings can dramatically increase HVAC demand. A dynamic approach to climate control is recommended, where HVAC intensity is adjusted in accordance with seasonal energy availability. Lighting, as one of the most energy-intensive components in vertical farming, must also be managed with precision to improve overall energy efficiency. Ideally, lighting should be concentrated during the daytime to coincide with periods of high PV output, thereby making the best use of self-generated solar energy. Furthermore, lighting intensity should be modulated based on environmental conditions, such as cloud cover, and the availability of energy stored in on-site batteries. During times when HVAC demand is high, lighting loads should be reduced accordingly to avoid overlapping energy peaks, which can place excessive stress on the system and increase reliance on grid electricity. All these recommendations are presented in Figure 15. Five decision rules are applied to optimize the start date of cultivation cycles. Rule 1 allows crop start when PV forecast exceeds 60%. Rule 2 delays or clusters crops during low-insolation months (January or December). Rule 3 reschedules start dates that coincide with predicted PV dips. Rule 4 favors months with lighting durations >12 hours, while Rule 5 prioritizes forecast-based timing when grid electricity cost is high. These rules form the basis for dynamic Gantt scheduling in the simulation model.

To classify scenarios based on their energy behavior and adaptability, a Principal Component Analysis (PCA) was conducted on key performance indicators derived from dynamic crop-cycle simulations. The input variables included: (i) maximum HVAC energy during crop cycles, (ii) minimum PV availability within the worst energy period, (iii) total energy consumption over full cycles, (iv) optimized energy under ideal scheduling, (v) absolute energy savings from schedule optimization, and (vi) crop cycle duration. These features were chosen to jointly reflect both energy stress and operational flexibility of each vertical farming scenario.

The PCA reduced the six-dimensional dataset into two principal components, which together captured over 80% of the total variance. Principal Component 1 (PC1) was primarily associated with maximum cycle energy and energy savings (both negatively), while showing a slight positive correlation with crop cycle length and optimized energy use. This axis effectively separates scenarios based on energy intensity and flexibility. Scenarios with strongly negative PC1 scores exhibit high baseline energy demand but strong optimization potential, whereas those on the positive end are generally stable, lower-energy systems with flatter energy profiles and minimal gains from dynamic intervention. Principal Component 2 (PC2) captured the trade-off between cycle duration and optimized energy. It correlated positively with crop cycle length, and negatively with optimized cycle energy, distinguishing longer-duration, efficient scenarios from short-cycle, high-demand cases. Together, PC1 and PC2 define a spectrum from short, intensive, energy-heavy setups to long-duration, energy-optimized configurations. To further interpret the scenario landscape, k-means clustering (k = 4) was applied to the PCA-transformed data, resulting in four distinct clustering groups:

• Cluster 0: High Demand, Rigid - Scenarios with heavy HVAC and lighting loads but limited optimization response. Likely require infrastructural interventions or high-reliability backup systems
• Cluster 1: Moderate, Responsive - Highly responsive to schedule shifts. Suitable for adaptive lighting or seasonally adjusted planting windows
• Cluster 2: High Demand, Optimizable - Initially high-demand scenarios with excellent response to cycle alignment, ideal for predictive control
• Cluster 3: Stable, Low-Demand - Inherently low-energy systems that show little gain from optimization, potential candidates for passive operation or fixed setpoints

**Figure 16.**
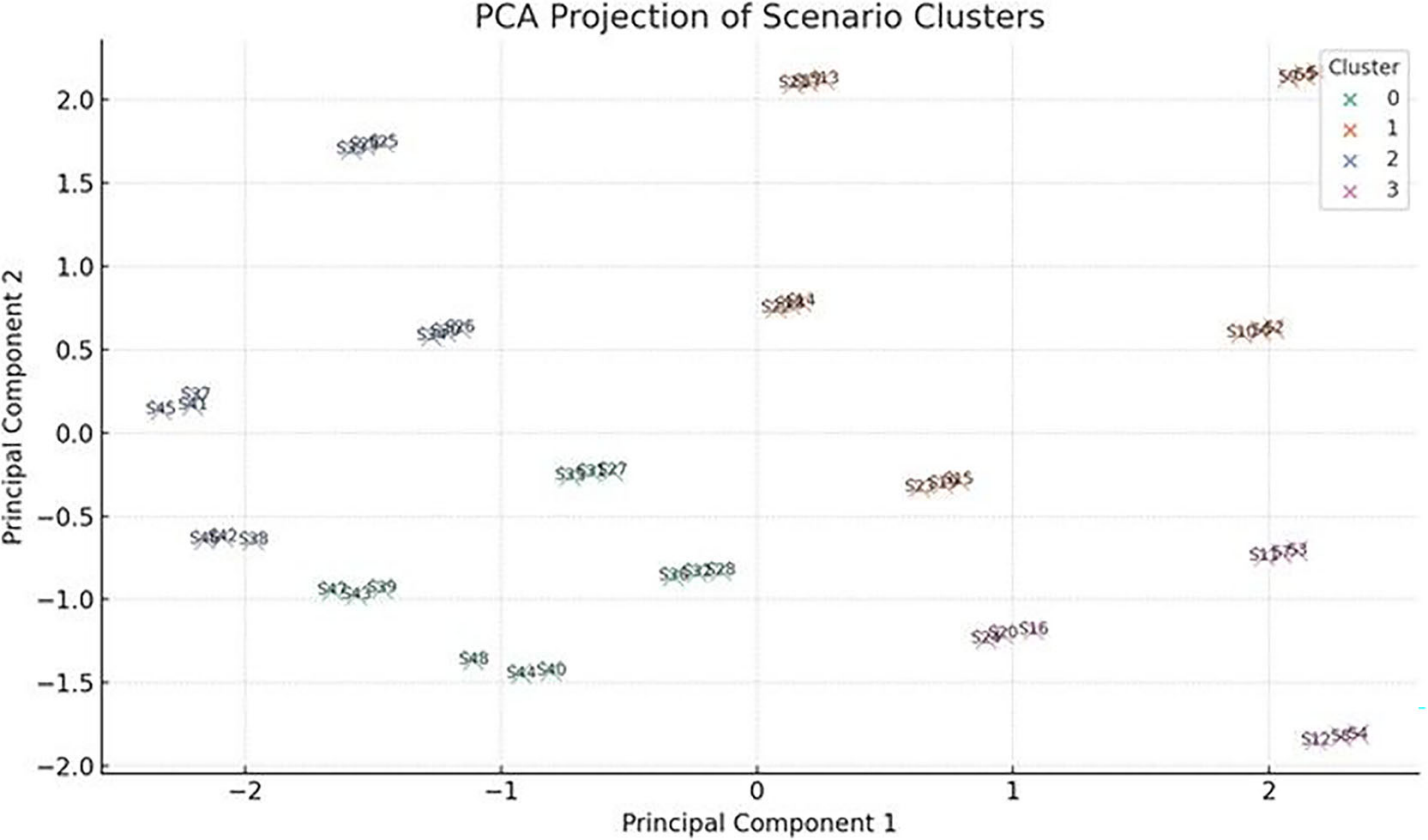
PCA of examined scenarios.

To further explore structural similarities among scenarios beyond individual resilience indicators, a Principal Component Analysis (PCA) was applied to key cycle-based performance metrics. The first two principal components explained more than 80% of the total variance ( Figure 16), enabling a two-dimensional representation of scenario behavior. Principal Component 1 (PC1) primarily captured energy intensity and optimization potential, separating high-demand, high-savings configurations from inherently stable, low-energy systems. Principal Component 2 (PC2) reflected the trade-off between crop cycle duration and optimized energy demand, distinguishing longer-duration, energy-efficient setups from shorter, more energy-intensive configurations. Subsequent k-means clustering (k = 4) performed in the PCA-reduced space identified four distinct scenario groupings. One cluster comprised high-demand but highly optimizable configurations, characterized by strong responsiveness to scheduling adjustments. Another cluster represented rigid, high-load scenarios with limited optimization gains. A third group included moderate-demand, responsive systems balancing efficiency and flexibility, while the final cluster consisted of stable, low-demand scenarios exhibiting minimal deviation between baseline and optimized performance. The PCA-based classification complements the resilience framework presented in Section 4.2 by revealing structural energy-behavior patterns that are not fully captured by annual autonomy or seasonal robustness metrics alone. In particular, scenarios such as S22 and S33 consistently appeared within high-performance clusters, reinforcing their balanced trade-offs between energy efficiency, adaptability, and renewable integration.

### 4.3 CO _2_ emissions comparison

***4.3.1 Introduction-comparative carbon footprint of vertical, greenhouse, and open-field farming.*** Quantifying and comparing the carbon of farming systems is essential for evaluating their environmental sustainability, especially under the lens of climate change and decarbonization targets. In the context of vertical farming (VF), which relies heavily on artificial energy inputs, understanding the specific emissions per kilogram of output is a critical step toward evaluating its viability against more traditional systems such as greenhouses and open-field agriculture. Among the available metrics, CO _2_ emissions per kilogram of produced lettuce in comparative studies is the most widely adopted indicator. This normalized unit allows for a consistent comparison across systems that differ significantly in scale, energy inputs, and yield densities. It also enables benchmarking of technological improvements, such as the integration of renewable energy systems or optimized climate control strategies. In this section, a comparison of the carbon emissions associated with VF, greenhouse, and open-field production systems takes place based on both published literature and simulation outputs.

***4.3.2 Comparison rationale.*** A comparative literature review of the carbon footprint associated with lettuce production across different agricultural systems reveals significant variation, primarily driven by energy inputs, yield density, and regional grid emission factors. Table 8 summarizes indicative values (in kg CO _2eq_/kg lettuce) from key studies.

**Table 8.** Overview on carbon footprint emissions for lettuce production.

Source	Production System/Location	CO _**2**_ Emissions (kgCO _**2**_/kg lettuce)	Scenario Description
3	Vertical Farming (NL)	15.2	VF unit powered by Dutch electricity mix (high fossil share), full artificial lighting.
	Greenhouse (NL)	2.4	High-yield semi-closed greenhouse with combined heat and power (CHP) and some supplemental lighting.
	Open Field (NL)	0.35	Conventional outdoor farming with minimal mechanization, no artificial lighting.
28	Vertical Farming (EU)	11.6–14.7	Range for fully enclosed systems with LED lighting and mechanical HVAC.
9	Vertical Farming (MY)	6.1–9.4	Hybrid PV-grid system in tropical climate with reduced HVAC demand.
6	Vertical Farming	6.9–12.0	Results vary by lighting intensity and climate (warmer climates yield lower HVAC loads).
8	Vertical Farming	≈9.8	Multiparametric optimization across different energy sources and lighting profiles.
5	VF (Global Range)	8.0–18.0	Aggregated data from multiple studies covering global setups, including worst-case fossil grid mixes.
4	Greenhouse (EU)	2.5–3.2	Range from low-tech to advanced greenhouses with CO _2_ fertilization and heat recovery.

Several key factors influence the variability of carbon emissions in vertical farming systems, with the most significant being the carbon intensity of the electricity grid. Vertical farms that operate in regions where the electrical grid relies heavily on fossil fuels, such as coal or natural gas, tend to exhibit substantially higher CO _2_ emissions. The energy source behind a vertical farm’s electricity supply is therefore a primary determinant of its environmental impact. Lighting, also, represents another major contributor to overall energy consumption in vertical farming. Consequently, the lighting strategy adopted has a direct effect on emissions. Approaches that reduce the Daily Light Integral (DLI), such as spectrum-tuned LEDs or photoperiod adjustments, as well as the implementation of dynamic lighting systems that respond to real-time energy availability or crop needs, can substantially decrease total emissions. Heating, ventilation, air conditioning, and dehumidification (HVACD) systems also play an increasingly important role in emission profiles, particularly in tropical climates or facilities with suboptimal insulation. In such contexts, the energy demands of HVACD systems can rival or exceed those of lighting. Optimizing these systems through the use of energy-efficient heat pumps, and smart thermal setpoint scheduling is essential for minimizing emissions without compromising crop quality or yield. Finally, the method of normalizing emissions data has a significant impact on the interpretation of sustainability metrics. While some studies express emissions per square meter of cultivated area, this approach can obscure meaningful comparisons across systems with varying productivity. In this analysis, all emission values are normalized per kilogram of fresh produce, providing a more accurate and consistent basis for assessing the environmental performance of different vertical farming configurations. This yield-based normalization ensures that emissions are contextualized relative to actual product output, supporting more reliable benchmarking and decision-making.

**Figure 17.**
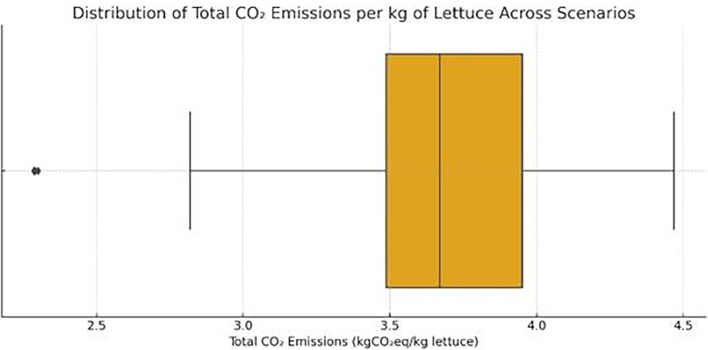
Distribution of CO _2_ emissions across all scenarios. Most values cluster between 3.2–3.8 kgCO _2_eq/kg lettuce, with outliers below 3 and above 4.

***4.3.3 Modeled carbon footprint of vertical farming scenarios.*** The scenario-specific total electricity consumption and corresponding life-cycle CO _2_ emissions per kilogram of harvested lettuce are summarized in Table 5. Emissions were calculated based on scenario-specific grid electricity consumption and operational inputs, as defined in the LCA framework described in Section 3.4. Across the 48 operational scenarios, the carbon footprint ranged from 2.29 to 4.47 kgCO _2_eq/kg lettuce, with an average value of 3.61 kgCO _2_eq/kg and a median of approximately 3.67 kgCO _2_eq/kg. Notably, 34 out of 48 scenarios exhibited emissions below the overall mean value. The observed variability reflects differences in lighting duration, HVAC loads, and PV contribution across seasonal configurations. Figure 17 illustrates the distribution of modeled emissions using a boxplot representation. Chart produced using Python and AI-assisted scheduling analysis. The interquartile range captures the concentration of most scenarios within a moderate emission band, while lower-end outliers correspond to reduced photoperiods and improved PV sufficiency. Conversely, high-emission scenarios are associated with extended lighting schedules and increased grid dependency. Although photovoltaic generation offsets a substantial share of electricity demand, residual reliance on the Greek electricity grid, characterized by moderate carbon intensity, remains the dominant contributor to overall emissions. Substrate-related impacts (rockwool and clay pellets) were included in the life-cycle assessment but contributed only marginally compared to operational electricity use. These findings indicate that emission reduction potential lies primarily in operational optimization strategies (e.g., dynamic lighting adjustment and shorter crop cycles) rather than material substitution alone.

### 4.4 Comparative evaluation with existing literature

**Figure 18.**
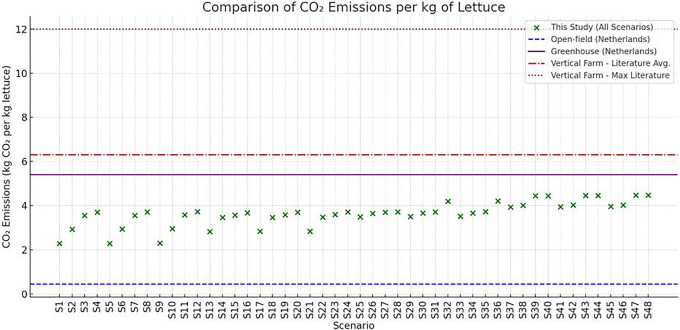
Comparison of CO _2_ per farming option.

The comparative analysis of CO _2_ emissions between the simulations of the examined VF system scenarios and established literature benchmarks reveals valuable insights into the environmental efficiency of the system. Previous studies have reported wide ranges of CO _2_ emissions per kilogram of fresh lettuce, depending on the cultivation system employed. Open-field agriculture typically exhibits the lowest footprint, as it relies entirely on ambient conditions and excludes artificial climate control or lighting inputs. ^3^^,^^8^ As expected, open-field farming has significantly lowest CO _2_ emissions, however this method limits lettuce production for a specific period in a year. Greenhouse production, offering higher yields and controlled environments, demonstrates average emissions mainly driven by supplemental lighting and heating demands. Vertical farming systems, on the other hand, present the highest carbon intensities, depending on the system configuration, location and energy mix. ^5^^,^^6^ These numbers stem from the near-complete reliance on artificial lighting as well as HVACD operations. Simulation results examined in this study showed that the best-performing scenarios reach values as low as 2.3 to 2.6 kg CO _2_/kg, clearly outperforming average vertical farm benchmarks, and approaching or even surpassing greenhouse performance in terms of carbon efficiency. The median value across all 48 scenarios remains below the upper greenhouse threshold and well within the reported vertical farming range. Additionally, the carbon intensity of the local electricity grid emerges as a critical variable, aligning with findings from earlier work. ^9^^,^^28^ For instance, a low-emission grid or partial integration of photovoltaics and battery storage, as concluded in the examined scenarios, may yield even lower CO _2_ emissions. Ultimately, while vertical farming still struggles to compete with open-field agriculture in carbon terms, our findings show that its footprint can be brought to a competitive level with greenhouses, especially when environmental control is optimized and renewable energy integration is enhanced. Figure 18 illustrates the comparative CO _2_ emissions per kilogram of fresh lettuce produced under four distinct systems: open-field cultivation, greenhouse production, vertical farming based on literature data, and all 48 scenarios simulated in this study. Each green cross represents a scenario from this study, spanning a range of energy strategies and crop cycle timings. The blue dashed line indicates the reported emissions from open-field cultivation in the Netherlands, the red dashed-dotted line represents emissions from greenhouse cultivation, while the magenta solid line and red dotted line represent the average and maximum values reported for vertical farms, respectively. Several scenarios in this study achieve substantially lower emissions than most vertical farming benchmarks, highlighting the importance of optimized scheduling and PV integration. Chart produced using Python and AI-assisted scheduling analysis. The results demonstrate that the majority of scenarios are clustered around 3.2–3.8 kg CO _2_/kg. These values are substantially lower than the average CO _2_ emissions reported in the literature for vertical farming, which typically range from 6.2 kg CO _2_/kg (average) up to 12 kg CO _2_/kg (maximum). Notably, the lowest-emission scenarios in this study (e.g., S1, S5, S9) approach the levels observed in greenhouse systems (5.4–5.5 kg CO _2_/kg) and, in rare cases, achieve emissions less than half of those reported in prior VF studies. Thus, greenhouse systems offer a middle ground, balancing environmental control with moderate energy use. In comparison, open-field systems maintain the lowest emissions footprint (~0.35 kg CO _2_/kg), owing to minimal energy input but suffer from climate dependency and limited spatial efficiency.

### 4.5 Impacts of dynamic lighting control on energy and crop cycle duration

To evaluate the potential for energy optimization through adaptive lighting strategies, each of the 48 scenarios were analyzed to determine the maximum safe lighting reduction -defined as the highest percentage reduction in lighting duration that did not increase total energy consumption due to elongation of the crop cycles’ duration. Based on this threshold, scenarios were classified into five distinct flexibility clusters: Highly Flexible, Upper Moderately Flexible, Low Moderately Flexible, Low Flexible, and Rigid ( Table 9).

**Table 9.** Lighting Flexibility Clusters by Max Safe Reduction.

Max Safe Lighting Reduction Cluster	Flexibility Class	Description
10%	Highly Flexible	Lighting can be reduced to 90% with no penalty. These scenarios are excellent candidates for dynamic lighting controls, especially in response to PV availability or peak load management.
7.5%	Upper Moderately Flexible	Scenarios that tolerate up to 7.5% reduction. These allow meaningful energy flexibility but may require adaptive scheduling to maintain efficiency.
5%	Low Moderately Flexible	Only small reductions are beneficial. Use with caution — partial flexibility possible without increasing HVAC energy.
2.5%	Low Flexible	Very limited flexibility. Best suited for mild demand response or fixed reductions on high-PV days only.
0%	Rigid	No lighting reductions lead to savings. Avoid dynamic lighting — maintain fixed photoperiods to avoid energy penalties due to prolonged HVAC operation.

**Figure 19.**
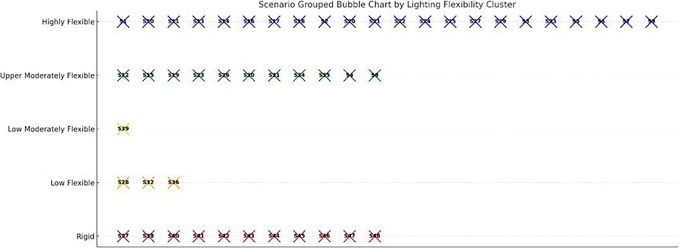
Scenarios grouped by lighting flexibility clusters. Highly flexible cases (blue) tolerate reduced lighting without energy penalty, while rigid cases (red) show no flexibility.

A visual cluster-based bubble chart was constructed, grouping scenarios according to these classifications and clearly highlighting their adaptability to lighting interventions ( Figure 19). Scenarios are categorized into five lighting flexibility clusters: Highly Flexible, Upper Moderately Flexible, Low Moderately Flexible, Low Flexible, and Rigid. This classification reflects the degree to which dynamic lighting strategies can be applied within each scenario to shift energy loads, minimize peak demands, or align with PV availability. Highly flexible scenarios (in blue) demonstrate the greatest potential for energy management optimization through lighting schedules. Chart produced using Python and AI-assisted scheduling analysis. Scenarios in the Highly Flexible group (e.g., S1, S2, S13) demonstrated the ability to tolerate up to 10% reduction in lighting without energy penalty, making them ideal candidates for dynamic lighting control strategies that respond to PV availability or energy pricing signals. In contrast, Rigid scenarios (e.g., S37 to S48) showed no flexibility, as even minor reductions led to increased total energy due to extended crop durations and elevated HVAC demands.

This clustering approach not only provides insight into the operational flexibility of each scenario but also guides the design of responsive control policies. For example, Low Flexible or Moderately Flexible scenarios may benefit from more conservative or seasonal lighting adjustments, while Highly Flexible configurations can adopt real-time adaptive schedules. This classification is especially valuable when coupled with sustainability metrics, allowing for multi-criteria decision-making based on energy efficiency, CO _2_ emissions, and operational resilience. Conversely, high-demand scenarios, especially those with long photoperiods or extreme HVAC loads, exhibited less flexibility. In these cases, the energy savings from reduced lighting were counterbalanced by increased cycle durations, resulting in little or no net benefit. An aggregate analysis across all 48 scenarios showed that lighting reductions of up to 10% consistently delivered measurable gains in energy savings, cost reduction, and carbon mitigation. However, in many scenarios beyond a threshold (e.g., 5%), the marginal benefits decrease, and the intervention became energetically inefficient due to extended growth durations. These findings confirm the feasibility of integrating dynamic lighting control in vertical farming, especially when guided by real-time PV generation forecasts or demand-response signals.

## 5. Conclusions

This study systematically evaluated the energy performance, environmental impact, and operational flexibility of a vertical hydroponic farming system under Northern Greece climatic conditions through a comprehensive simulation framework. Using TRNSYS 18-based modeling, 48 cultivation scenarios for lettuce production were analyzed, incorporating variations in photoperiod, humidity, HVACD setpoints, and renewable energy integration. A multi-criteria decision framework was introduced encompassing energy per cycle, emissions, and resilience. The results demonstrate that electricity demand in vertical farming is highly sensitive to crop cycle configuration, with artificial lighting and HVACD loads being the dominant contributors. By aligning crop cycles with PV availability, optimized schedules showed measurable reductions in grid reliance and excess generation. These insights were further extended into a proposed dynamic planting calendar and adaptive setpoint strategies for lighting and HVAC systems. Scenarios with longer photoperiods and extreme temperature differentials exhibited higher energy intensity and grid dependency. However, aligning crop schedules with seasons of milder weather conditions, particularly autumn and spring, allowed for improved PV utilization and lower energy demand from grid. The study also revealed marginal variability in carbon emissions across scenarios, ranging from 2.29 to 4.47 kg CO _2_/kg lettuce. Notably, the best-performing configurations achieved emission levels outperforming even greenhouse systems and significantly better than previously reported VF benchmarks. These gains were achieved through strategic timing for optimal energy crop cycles, environmental control and integration with a PV-battery system. A combined assessment using indicators such as Grid Dependency Ratio (GDR), Grid Independence Index (GII), and Seasonal Resilience Score (SRS) enabled a multi-dimensional classification of scenario performance. Scenarios with low grid reliance and stable PV alignment were identified as optimal candidates for future deployment in energy-constrained or off-grid contexts. A key innovation of this work is the Lighting Flexibility Classification System, where each scenario was assessed for its capacity to safely reduce photoperiod without increasing total energy demand. Scenarios were grouped into five clusters, Highly Flexible, Upper Moderately Flexible, Low Moderately Flexible, Low Flexible, and Rigid, based on the maximum safe lighting reduction achievable. These classifications offer a practical guideline for implementing dynamic lighting interventions. For instance, scenarios like S1, S2, and S13 were found to tolerate up to 10% lighting reduction with net energy savings, making them ideal candidates for real-time lighting control. On the other hand, Rigid scenarios, primarily those with extreme temperature or humidity requirements (e.g., S37–S48), showed no capacity for lighting reduction without incurring increased HVAC energy costs. Overall, the integration of lighting flexibility, crop cycle adaptation, and renewable alignment advances our ability to design sustainable and intelligent vertical farming operations. The decision tools developed herein, including radar plots, quadrant maps, and bubble cluster charts, can guide farm designers, policy makers, and engineers in selecting optimal environmental strategies. Future work will extend this model to include real-time predictive control using live PV forecasts and feedback from plant physiology to further optimize energy use and crop yield outcomes. In conclusion, the findings confirm that vertical farming, while energy-intensive, can be significantly optimized through seasonal crop scheduling, moderate environmental control, and targeted lighting strategies. Under the right configuration, vertical farms can approach or even outperform greenhouse systems in both energy and carbon metrics, especially when partially powered by renewable sources. Future work should explore the integration of real-time environmental sensing, AI-driven lighting algorithms, and scalable PV-storage systems to further improve system autonomy, productivity, and sustainability. The multiple regression model shows that, when humidity levels remain within the acceptable range for proper plant growth, the scenarios indicate that variations in indoor humidity have a negligible impact on energy consumption.

## Ethics and consent

Ethics approval and consent are not required.

## Data Availability

**Zenodo**: Dynamic Energy Optimization and Lighting Flexibility Classification for Sustainable Vertical Farming: A Simulation-Based Multi-Scenario Analysis https://doi.org/10.5281/zenodo.16926135. ^26^ This project contains the following underlying data:
• TRNSYS 18 input files (tpf/log/txt/pti/lst/idf/dck/bld/plt) input configuration files used for running the 48 cultivation scenarios. • Environmental Datasheets (tm2) raw environmental data used as model inputs • Simulation Results (txt) raw and processed outputs from TRNSYS 18 simulations across all scenarios. •
Figure values (csv) data underlying figures and plots presented in the article **Zenodo**: Dynamic Energy Optimization and Lighting Flexibility Classification for Sustainable Vertical Farming: A Simulation-Based Multi-Scenario Analysis https://doi.org/10.5281/zenodo.16926135 ^26^
• Equipment Manuals (pdf ) Manuals for the existing equipment in the existing isobox • Python Scripts for scenario analysis (txt) Scripts used to process simulation results • Scenarios and supplementary tables and data (xlsx/csv) All data for the 48 scenarios analysis •
Figures and Scenarios optimal crop profiles (jpg/png) All resulting figures from the analysis •
Tables (docx) All tables used in the final paper The authors confirm that no applicable reporting guidelines (e.g. CONSORT, STROBE, PRISMA) exist for the simulation-based methodological study presented herein as verified through the EQUATOR and FAIRSharing databases.

## License

Data are available under the terms of the Creative Commons Attribution 4.0 International license (CC-BY 4.0).

## Restrictions

Due to software licensing restrictions and proprietary dependencies of TRNSYS 18, the complete project files (including component libraries and simulation workspace) cannot be shared publicly. Sharing these files would require distribution of third-party licensed material that is not permitted under open access. These files are part of the licensed TRNSYS software package, which is distributed by the Transsolar Software Engineering: trnsys.de. Corresponding personnel: Alina Wagner email: hotline@Transsolar.com. Access to these resources require a valid TRNSYS licence.
