# Supplementary material for: Dynamic Energy Optimization and Lighting Flexibility Classification for Sustainable Vertical Farming: A Simulation-Based Multi-Scenario Analysis
Source: Open Res Eur. 2026 May 6;5:316. Originally published 2025 Oct 16. [Version 2] doi: 10.12688/openreseurope.20847.2 (PMC13202201; doi:10.12688/openreseurope.20847.2)
Supplement: Peer review history

# Reviewer report: Reviewer response for version 2

**DOI:** 10.21956/openreseurope.25420.r73915

**Published:** 2026-05-25

**Authors:** Yuyao Kong

**Affiliations:** 1 University of Florida, Florida, USA

**Introduction:**

1. “previous comparative studies have shown that…” please add citations.
2. MPPT, this is the first time it shows up in the main text, please provide the full name of this term.
3. The term “near-zero” is ambiguous and should be explicitly defined. In the same sentence, the statement refers to “recent studies” but does not provide sufficient citations.
4.  “However, such approaches typically rely on ..” please provide more detail on the methodologies used in prior studies to evaluate these parameters.
5. The phrase “multipored model” is unclear.
6. “J. Pimentel’s study explored different strategies…” it might be helpful if provide more details.
7. “A case study in Malaysia assesses ..” please provide reference
8. “Despite the growing body of…” please provide references
9. “Using TRNSYS 18…” what is it? Please provide full name and include the full software reference for TRNSYS 18, including the developer/vendor information, version number, and appropriate citation to improve reproducibility and methodological transparency.
10. “The objective is to identify … that minimize energy use per unit of harvested biomass” this is the place where the authors should define “Specific Energy Consumption (SEC)”, instead of defining it in the second section (the scope of the study).

Scope of the study: What is the purpose of this section? It looks not necessary and some of the content can be briefly integrated into methods section. Also, please avoid using the bold formatting in the main text, except for titles and headings, as they are more like a ChatGPT format.

**Methods:**

1. There are many repeated words, should be rewritten to improve conciseness.
2. The content presented in the “Scope of Study” section and the paragraphs preceding Section 3.1 appears repetitive. Consider consolidating these parts into a single, more concise section to improve readability and avoid unnecessary duplication. Please maintain a clear and logical flow and focus on the most relevant methodological details.
3. There are too many details about lettuce, there is no need to introduce lettuce in detail. Scientific name, cultivar, vendor information is sufficient.
4. Were lettuce grown under all 48 scenarios? How many plants in each unit? How long was the planting cycle?
5. “Τhe harvested mass per cultivation cycle was assumed constant across all scenarios, based on …” I don’t understand it, how can different scenarios with different environmental conditions result in the same yield?
6. I don’t understand why the authors provide a detailed list of literature reviews in this section, it can be summarized into one sentence with references cited.
7. I feel that Figure 1 and 2 can be combined into a single figure with subpanels (e.g., Figure 1A and Figure 1B). The production system can be introduced at one place. Right now they are in wo different sections and make it difficult to follow.
8. I couldn’t read Figure 3 although I have downloaded it. Please improve the figure resolution.
9. Section 3.2 about the equation: “ All electrical power consumed by the LED lighting system was assumed to be fully converted into internal sensible heat...” How about radiative and convective heat fractions?
10. The statement under the equation is too long and “Transpiration rate was derived from …” is repetitive words, it has been described above.  The remaining parts of the paragraph should be in the main text.

**Section 3.2:**

1. “The indoor environmental conditions were tailored for safe lettuce growth.” What does “safe lettuce growth” mean here? Do you mean normal growth?
2. I don’t understand why Table 3 is shown here. It looks like results? Instead, Table 4 needs to be moved here.
3. Figure 4 should be in results section.

**Section 3.5:** please avoid using the bold formatting in the main text.

**Section 3.7:** “For each scenario… focuses on annual operational energy flows and associated CO _2_ emissions” this can be deleted or shortened as it is repetitive information.

**Section 3.8:**

1. “To support adaptive energy and schedule optimization … on crop timing under various scenarios”. This should be in discussion section.
2. This is repetitive words (it’s similar information in the beginning of the paragraph).
3. “Thus, for…” is there missing content?
4. Equation 7 should be moved above annotations.
5. Table 6: the scenarios were not listed in order in the table.

**Section 4:**

1. I could not see a, b, c, d in Figure 5 but the authors used Figure 5a, 5b, 5c, 5d in the text.
2. Also the resolution of image is too low and I could not read axes titles.

General comments for the whole manuscript: The text has a lot of repetitive content and has to be reorganized to flow logically.

Is the study design appropriate and does the work have academic merit?

Yes

Is the work clearly and accurately presented and does it cite the current literature?

Partly

If applicable, is the statistical analysis and its interpretation appropriate?

I cannot comment. A qualified statistician is required.

Are all the source data underlying the results available to ensure full reproducibility?

Yes

Are the conclusions drawn adequately supported by the results?

Yes

Are sufficient details of methods and analysis provided to allow replication by others?

Partly

Reviewer Expertise:

Controlled Environment Agriculture

I confirm that I have read this submission and believe that I have an appropriate level of expertise to confirm that it is of an acceptable scientific standard, however I have significant reservations, as outlined above.

# Reviewer report: Reviewer response for version 2

**DOI:** 10.21956/openreseurope.25420.r73708

**Published:** 2026-05-23

**Authors:** Adnan Arshad

**Affiliations:** 1 Lanzhou University, Lanzhou, China

The author has made a comprehensive revision and addressed most of the comments; I suggest that the author revise figure 3—configuration of the TRNSYS 18 model for the vertical farming unit. -How is battery operation scheduled – rule-based (e.g., charge from PV excess, discharge when PV < load) or optimized? This affects how much grid electricity is displaced. -What is the temporal resolution of the emission factor? Monthly or hourly variation in grid carbon intensity could significantly affect results if the system uses grid electricity at different times of day. -Why 48 scenarios (S1–S48)?

 Here are a few follow-up comments/suggestions:

 - For spring-start cycles like S17 (day 72–120), is the energy saving primarily due to avoided cooling, avoided heating, or better PV matching? What is the dominant HVACD component?

 - PV coverage threshold: Figure 14; Are these fixed annual averages, or month-by-month dynamic thresholds from TRNSYS 18 simulations?

 - Would the same day-of-year ranking hold for a different climate or is this result location-specific?

 - what operational lifetime (years) would VF’s higher embodied carbon be amortized relative to greenhouse/open-field? Does your comparison assume local open-field only during optimal outdoor seasons, or an annualized average including imports?

Is the study design appropriate and does the work have academic merit?

Yes

Is the work clearly and accurately presented and does it cite the current literature?

Yes

If applicable, is the statistical analysis and its interpretation appropriate?

Yes

Are all the source data underlying the results available to ensure full reproducibility?

Yes

Are the conclusions drawn adequately supported by the results?

Partly

Are sufficient details of methods and analysis provided to allow replication by others?

Yes

Reviewer Expertise:

NA

I confirm that I have read this submission and believe that I have an appropriate level of expertise to confirm that it is of an acceptable scientific standard, however I have significant reservations, as outlined above.

# Reviewer report: Reviewer response for version 2

**DOI:** 10.21956/openreseurope.25420.r73709

**Published:** 2026-05-21

**Authors:** Nima Asgari

**Affiliations:** 1 Western University, London, Ontario, Canada

The authors have put effort on addressing my comments.

Is the study design appropriate and does the work have academic merit?

Yes

Is the work clearly and accurately presented and does it cite the current literature?

No

If applicable, is the statistical analysis and its interpretation appropriate?

I cannot comment. A qualified statistician is required.

Are all the source data underlying the results available to ensure full reproducibility?

Yes

Are the conclusions drawn adequately supported by the results?

Yes

Are sufficient details of methods and analysis provided to allow replication by others?

No

Reviewer Expertise:

Solar PV, HVAC, Indoor Agrivoltaics

I confirm that I have read this submission and believe that I have an appropriate level of expertise to confirm that it is of an acceptable scientific standard.

# Reviewer report: Reviewer response for version 1

**DOI:** 10.21956/openreseurope.22555.r65518

**Published:** 2025-12-30

**Authors:** Nima Asgari

**Affiliations:** 1 Western University, London, Ontario, Canada

While the research titled “ **Dynamic Energy Optimization and Lighting Flexibility Classification for Sustainable Vertical Farming: A Simulation-Based Multi-Scenario Analysis**” provides valuable insights, significant level of revision is still necessary to ensure alignment with indexing standards. Otherwise, the article is not suitable for indexing.

1. Abstract: include the most significant achievements of the study both in qualitative and quantitative form. Numeric results are rarely included in current version.
2. Introduction: The literature review is too weak and completely unacceptable. Add the reviewed findings of recent and relevant research. Look at our recent research and the reviewed studies in them:

- Reference 1: https://www.mdpi.com/2071-1050/16/14/6120
- Reference 2: https://papers.ssrn.com/sol3/papers.cfm?abstract_id=5104787
- Reference 3: https://papers.ssrn.com/sol3/papers.cfm?abstract_id=5775183

1. Scope of the study: in the text: “…electricity consumption per unit of yield (expressed in kWh/kg)…”; The technical term for this variable is Specific Energy Consumption (SEC).
2. Scope of the study: Use the last paragraph of this section, “This schematic shows the pilot …. was provided by the manufacturer.” as a description for the Figure 1, right after the place you cite this figure for first time. The same issue is for Figure 2 and the last paragraph of section 3.1. All other figures have the same problem. Their description has been fell apart from their first citing location in the text.
3. Methodology: “… where PV supply is directed first to loads, **then** to the battery, and finally to the grid”.
4. Methodology: The introductory descriptions before section 3.1 are repetitive, redundant and sometimes ambiguous. You need to make them short, sharp and to the point, and instead, bring more details in the following sections.
5. Methodology: why have you done a lit review in Section 3.1? (“Ch. Vatistas et compared the lettuce cultivation between GHs and VFs and presents that VFs are more efficient than GHs in terms of yield per unit area, but require more energy to operate”). It is not standard. You must discuss about the previous studies either in the Introduction or the Discussion.
6. Methodology: In the second paragraph of section 3.1: You need to justify your claims about the 16-hour photoperiod by providing sufficient information about PPFD and more importantly DLI.
7. Methodology: Table 1, Battery voltage is 2? You have mentioned that your case was 24 volt. Please indicate the specifications for your case.
8. Methodology: “Harvest took place at physiological maturity (~120–150 g fresh weight per head), …”. What is your reference for this?
9. Methodology: “Vertical farming system is realized in ISOBOX with galvanized metal construction…”. This is firs time that you have talked about an ENCLOSURE in your text. This is important information which justifies the necessity for your HVAC system. You need to provide all basic and important information about your system in section 2. Scope of the study. You should assume that the readers DO NOT KNOW ANYTHING about your research. Hence, you should try to visualize the construction and the concept of your study in all introductory sections such as Abstract, Last paragraph of Introduction, Scope of the study, and Methodology.
10. Section 3.1: “All these components of the VF system are presented on the (Table 1) in the Manuscript Tables section”. What do you mean by mentioning “ **Manuscript Tables section**”?
11. Methodology: the resolution of Figure 3 & 4 is extremely low! You must replace them.
12. Methodology: there is a lot of repetitive content in the Methodology section. You need to shorten them and make them to the point and mentioned once at the right place.
13. Table 3: “No significant effect on crop duration” should be “No significant effect on crop **cycle** duration”.
14. Crop Cycle function: Photoperiod alone is not the only affecting parameter of lighting system, PPFD, thereby DLI matters a lot. You MUST indicate that this equation (fitted curve) is only valid for the controlled PPFD range of XXX-YYY, whatever is in your study.
15. Figure 4: This plot is not scientifically acceptable. The angle of plot does not provide sufficient information about the surface behavior. Rotate the plot to present the surface in an appropriate angle.
16. Figure 5: Completely non-readable titles and tick mark labels. Must be replaced. Instead of addressing like top left corner, it is recommended to separate them into four a, b, c, and d and address them like Figure 5a, etc.
17. Figure 5: This figure is actually part of results and along with all discussions provided MUST be transferred to Section 4. Same issue with Table 5 and the corresponding discussions.
18. Section 3.4: Long form of LCA must be defined at first-time-use place.
19. Figure 6 needs to have axes titles.
20. Figure 8: low quality-no unit mentioned! Avoid using AI to refine the pictures. You do not have to do that. The exports from Microsoft Excel, Python or other softwares can be still of good quality.
21. Equations 4-6: Put references.
22. Resilience and equations 4-6 must have been introduced in the Methods section. You have not mentioned anything about this investigation approach neither in methods nor in the scope of the study.
23. The same problem with PCA and Figure 15. The authors have tried to include all analytic methods and increase the content without following the standard article preparation layouts. Adding new evaluation approaches in the middle of results section distracts readers from the outcomes. I recommend either removing PCA analysis or shortening the content of pages 15-24 into two-three pages to select the best scenario(s). You definitely need to define them in Methods and Scope.
24. Again the methods about dynamic lighting control in the middle of results!
25. Table 7: The first study does not have the source. This should not be in Results section if you don’t plan to compare with the outcomes of your study.
26. Figure 18 is very useful.
27. General comment for the whole manuscript: remove titles from all your figures.
28. General comment for the whole manuscript: The text is too long with a lot of excessive and redundant content. You have described your methodology, results, and approach taken to produce the results multiple times with different sentences. This is not a standard method of writing.
29. General comment for the whole manuscript: Avoid using — in the text which is basically introduced by ChatGPT.
30. General comment for the whole manuscript: Although they are minor but are important. You must review the whole manuscript to address the grammatical issues with the text. For example: “ **on the (Table 1)**” must be “ **in Table 1**”, and so on.
31. I’m not sure if you intendedly have provided descriptive text of figures at the last paragraph of each section or it has happened during the submission process. It is highly recommended to solve this issue and bring all descriptions right after where you cite the figure for first time.

Is the study design appropriate and does the work have academic merit?

Yes

Is the work clearly and accurately presented and does it cite the current literature?

No

If applicable, is the statistical analysis and its interpretation appropriate?

I cannot comment. A qualified statistician is required.

Are all the source data underlying the results available to ensure full reproducibility?

Yes

Are the conclusions drawn adequately supported by the results?

Yes

Are sufficient details of methods and analysis provided to allow replication by others?

No

Reviewer Expertise:

Solar PV, HVAC, Indoor Agrivoltaics

I confirm that I have read this submission and believe that I have an appropriate level of expertise to confirm that it is of an acceptable scientific standard, however I have significant reservations, as outlined above.

## References

[1] : Net Zero Agrivoltaic Arrays for Agrotunnel Vertical Growing Systems: Energy Analysis and System Sizing. *Sustainability* .2024;16(14) : 10.3390/su16146120 10.3390/su16146120

[2] : Lighting and Revenue Analysis of Grow Lights in Agrivoltaic Agrotunnel for Lettuces and Swiss Chard.2025; 10.2139/ssrn.5104787 10.2139/ssrn.5104787

[3] : Technoeconomic Analysis of Indoor Basil Cultivation in Agrivoltaic Agrotunnels with Optimized Near-Zero Energy Systems.2025; 10.2139/ssrn.5775183 10.2139/ssrn.5775183

# Response

**Published:** 2026-03-10

**Authors:** Chrysovalantis Ketikidis

**1 ^st ^Comment: Abstract: include the most significant achievements of the study both in qualitative and quantitative form. Numeric results are rarely included in current version.** **Suggested Reply: **The abstract has been revised to explicitly include key quantitative outcomes of the study, in line with indexing standards. Numerical results have been added to highlight the achieved carbon footprint (median 3.67 kg CO₂ kg⁻¹ of lettuce), the range of crop cycle durations (34–52 days), the contribution of photovoltaic energy to annual electricity demand in optimized scenarios, and the maximum lighting reduction (up to 10%) achievable without increasing total energy consumption. These additions improve the clarity and visibility of the study’s main achievements while preserving the concise structure of the abstract.

**2 ^nd^ Comment: Introduction: The literature review is too weak and completely unacceptable. Add the reviewed findings of recent and relevant research. Look at our recent research and the reviewed studies in them:**

- Reference 1: https://www.mdpi.com/2071-1050/16/14/6120
- Reference 2: https://papers.ssrn.com/sol3/papers.cfm?abstract_id=5104787
- Reference 3: https://papers.ssrn.com/sol3/papers.cfm?abstract_id=5775183

**Suggested Reply: **The Introduction has been substantially revised to strengthen the literature review, incorporating recent and relevant studies on PV-integrated controlled-environment agriculture, lighting optimization, and technoeconomic assessments, including the works suggested by the reviewer. The revised text now explicitly discusses the contributions and limitations of these studies and clearly positions the present work within the identified research gap, namely the lack of an integrated, scenario-based framework linking seasonal renewable availability, crop cycle timing, and operational flexibility with resilience and grid-dependency indicators.

**3 ^rd^ Comment: Scope of the study: in the text: “…electricity consumption per unit of yield (expressed in kWh/kg) …”; The technical term for this variable is Specific Energy Consumption (SEC).**

**Suggested Reply: **The manuscript has been revised to adopt the standard terminology Specific Energy Consumption (SEC) for electricity consumption per unit of harvested yield. SEC (kWh kg⁻¹) is now defined at its first occurrence in the Scope of the Study, and the term is used consistently throughout the manuscript.

**4 ^th^ Comment: Scope of the study: Use the last paragraph of this section, “This schematic shows the pilot …. was provided by the manufacturer.” as a description for the Figure 1, right after the place you cite this figure for first time. The same issue is for Figure 2 and the last paragraph of section 3.1. All other figures have the same problem. Their description has been fell apart from their first citing location in the text.** **Suggested Reply: **The manuscript has been revised to address this issue for all figures. Specifically, the descriptive text previously placed as standalone paragraphs at the end of sections has now been relocated to the body text, immediately following the first citation of each figure (Figures 1–19). This ensures that each figure is introduced and described at its first point of reference in accordance with standard publishing and indexing practices.

**5 ^th^ Comment: Methodology: “… where PV supply is directed first to loads, then to the battery, and finally to the grid”.**

**Suggested Reply: **Then was added to the manuscript  

**6 ^th^ Comment: The introductory descriptions before section 3.1 are repetitive, redundant and sometimes ambiguous. You need to make them short, sharp and to the point, and instead, bring more details in the following sections.**

**Suggested Reply: **The introductory part of the Methodology section has been substantially revised to improve clarity and conciseness. The text was shortened and restructured to provide a clear overview of the methodological workflow without repeating detailed information. Descriptive and technical details previously included in the introductory paragraphs were relocated to the corresponding subsections (Sections 3.1-3.8), where they are discussed in a more structured manner. The revised introduction now briefly outlines the sequence of methodological steps, including cultivation procedure definition, system setup, scenario development, dynamic simulation, resilience indicator formulation, multivariate scenario classification, carbon footprint estimation, and adaptive lighting control, allowing the reader to better follow the analytical framework of the study.

**7 ^th^ Comment: Methodology: why have you done a lit review in Section 3.1? (“Ch. Vatistas et compared the lettuce cultivation between GHs and VFs and presents that VFs are more efficient than GHs in terms of yield per unit area, but require more energy to operate”). It is not standard. You must discuss about the previous studies either in the Introduction or the Discussion**.

**Suggested Reply: **The cited comparative statement was removed from the Methodology section and incorporated into the Introduction, in line with standard academic structure, where previous studies and contextual background are discussed.

**8 ^th^ Comment: Methodology: In the second paragraph of section 3.1: You need to justify your claims about the 16-hour photoperiod by providing sufficient information about PPFD and more importantly DLI.**

**Suggested Reply: **The justification of the 16-hour photoperiod was strengthened by explicitly linking photoperiod, PPFD, and daily light integral (DLI), using the literature data already summarized in Table 2. The revised text clarifies that the applied lighting conditions correspond to DLI ranges commonly reported for lettuce cultivation in vertical farming and indoor controlled-environment systems.

**9 ^th^ Comment: Methodology: Table 1, Battery voltage is 2? You have mentioned that your case was 24 volts. Please indicate the specifications for your case.**

**Suggested Reply: **The battery system operates at a nominal voltage of 24 V. The value of 2 V reported in Table 1 corresponds to the nominal voltage of individual battery cells. This clarification has been added to the System setup section to explicitly describe the series-connected 2 V cell configuration forming the 24 V battery bank.

**10 ^th^ Comment: Methodology: “Harvest took place at physiological maturity (~120–150 g fresh weight per head), …”. What is your reference for this?**

**Suggested Reply: **The reported harvest weight of ~120–150 g per head corresponds to physiological maturity observed in the pilot vertical farming unit used in this study and is consistent with values commonly reported in the literature for lettuce cultivated under controlled-environment and vertical farming conditions. This clarification and supporting reference have been added to the revised manuscript.

**11 ^th^ Comment: Methodology: “Vertical farming system is realized in ISOBOX with galvanized metal construction…”. This is first time that you have talked about an ENCLOSURE in your text. This is important information which justifies the necessity for your HVAC system. You need to provide all basic and important information about your system in section 2. Scope of the study. You should assume that the readers DO NOT KNOW ANYTHING about your research. Hence, you should try to visualize the construction and the concept of your study in all introductory sections such as Abstract, Last paragraph of Introduction, Scope of the study, and Methodology.**

**Suggested Reply: **Scope of the study was enhanced to explicitly explain why the fully enclosed nature of the vertical farming unit necessitates continuous HVACD operation. The revised text clarifies that internal sensible and latent heat loads from lighting and crop transpiration cannot be passively dissipated, making active climate control a functional requirement rather than a design choice. Brief contextual references to the enclosed system have also been added to the Abstract and Introduction.

**12 ^th^ Comment: Section 3.1: “All these components of the VF system are presented on the (Table 1) in the Manuscript Tables section”. What do you mean by mentioning “Manuscript Tables section”?**

**Suggested Reply: **The reference to a “Manuscript Tables section” was introduced during the submission process, where all tables were required to be extracted from the main text and compiled after the References section. To avoid any confusion for the reader, this wording has been removed, and the text now refers directly to Table 1, which presents the technical components of the vertical farming system.

**13 ^th^ Comment: Methodology: the resolution of Figure 3 & 4 is extremely low! You must replace them.**

**Suggested Reply:** The resolution of Figures 3 and 4 has been improved in the revised manuscript. Both figures have been replaced with high-resolution versions to ensure better readability of the graphical elements and labels.

**14 ^th^ Comment: Methodology: there is a lot of repetitive content in the Methodology section. You need to shorten them and make them to the point and mentioned once at the right place.**

**Suggested Reply: **The Methodology section has been carefully revised to reduce repetitive content and improve clarity and conciseness

**15 ^th^ Comment: Table 3: “No significant effect on crop duration” should be “No significant effect on crop cycle duration”.**

**Suggested Reply: **The word cycle was added in Table 3

**16 ^th^ Comment: Crop Cycle function: Photoperiod alone is not the only affecting parameter of lighting system, PPFD, thereby DLI matters a lot. You MUST indicate that this equation (fitted curve) is only valid for the controlled PPFD range of XXX-YYY, whatever is in your study.**

**Suggested Reply: **The validity range of the crop cycle regression model has now been explicitly clarified in the manuscript. Equation (3) is stated to apply only within the controlled PPFD (200–350 μmol·m⁻²·s⁻¹) range investigated in this study, avoiding extrapolation beyond the examined lighting conditions.

**17 ^th^ Comment: Figure 4: This plot is not scientifically acceptable. The angle of plot does not provide sufficient information about the surface behavior. Rotate the plot to present the surface in an appropriate angle.**

**Suggested Reply: ** The figure has been revised with an adjusted elevation and azimuth to better illustrate the linear gradient of crop cycle duration as a function of photoperiod and ΔT. Additionally, contour projections have been added to improve interpretability. The revised figure now clearly demonstrates the dominant influence of photoperiod and the secondary effect of temperature differential.

**18 ^th^ Comment: Figure 5: Completely non-readable titles and tick mark labels. Must be replaced. Instead of addressing like top left corner, it is recommended to separate them into four a, b, c, and d and address them like Figure 5a, etc.**

**Suggested Reply: ** Figure 5 has been reformatted to improve readability, with enlarged axis labels and tick marks. The panels have been separated and clearly labeled as Figure 5a–d, and the manuscript text has been updated accordingly.

**19 ^th^ Comment: Figure 5: This figure is actually part of results and along with all discussions provided MUST be transferred to Section 4. Same issue with Table 5 and the corresponding discussions.**

**Suggested Reply: **Figure 5 and its associated interpretative discussion have been removed from the Methodology section and transferred to Section 4 (Results), where the dynamic annual system behavior is now presented under Section 4.1. The related descriptive content has been substantially condensed to focus strictly on result interpretation rather than methodological explanation. Similarly, Table 5 and its corresponding discussion of scenario-specific CO₂ emissions have been relocated from Section 3.4 to Section 4.3. The Methodology section (3.4) now includes only the LCA framework, system boundaries, emission factors, and calculation approach, while all quantitative outcomes and their interpretation are presented exclusively in the Results section.

**20 ^th^ Comment: Section 3.4: Long form of LCA must be defined at first-time-use place.**

**Suggested Reply: ** he full term “Life Cycle Assessment (LCA)” has now been defined at its first occurrence in Section 3.4. The term “Global Warming Potential (GWP)” has also been clarified accordingly.

**21 ^th^ Comment: Figure 6 needs to have axes titles.**

**Suggested Reply: ** Axis titles have been added to Figure 6, including “Month” on the x-axis and “PV Energy Production (kWh)” on the y-axis, improving clarity and readability.

**22 ^th^ Comment: Figure 8: low quality-no unit mentioned! Avoid using AI to refine the pictures. You do not have to do that. The exports from Microsoft Excel, Python or other softwares can be still of good quality.**

**Suggested Reply: **The unit (kWh) has now been explicitly added to the color scale of Figure 8.                

**23 ^th^ Comment: Equations 4-6: Put references**

**Suggested Reply: **Equations (4) to (6) represent performance indicators formulated within the scope of the present study. These indices are derived directly from annual and monthly energy balance relationships obtained from the TRNSYS simulation outputs and were developed to quantify grid independence and seasonal PV resilience across scenarios. They do not correspond to previously published closed-form equations but are ratio-based indicators grounded in standard energy balance principles commonly applied in distributed energy system analysis. To clarify this, additional explanatory text has been added in Section 4.2 explicitly stating that these metrics are study-specific derived indicators.

**24 ^th^ Comment: Resilience and equations 4-6 must have been introduced in the Methods section. You have not mentioned anything about this investigation approach neither in methods nor in the scope of the study.**

**Suggested Reply: ** In the revised manuscript, the resilience assessment framework has been clearly introduced in the Methodology section. The Grid Independence Index (GII) and Seasonal Resilience Score (SRS) are now formally defined prior to their application in the Results section, and Equations (4) - (6) have been relocated accordingly. Additionally, the cycle-based resilience evaluation and the classification of scenarios into descriptive energy-performance typologies are now described in the Methods section to ensure a clear separation between methodological formulation and result interpretation. The Results section (Section 4.2) now focuses exclusively on the interpretation of the resilience maps and scenario performance, without introducing new analytical formulations.

**25 ^th^ Comment: The same problem with PCA and Figure 15. The authors have tried to include all analytic methods and increase the content without following the standard article preparation layouts. Adding new evaluation approaches in the middle of results section distracts readers from the outcomes. I recommend either removing PCA analysis or shortening the content of pages 15-24 into two-three pages to select the best scenario(s). You definitely need to define them in Methods and Scope.**

**Suggested Reply: **In the revised manuscript, the PCA-based analysis has been substantially streamlined and structurally reorganized to improve clarity and alignment with standard article preparation layouts. First, the methodological description of the PCA and k-means clustering procedure has been moved to the Methodology section, where the input variables, dimensionality reduction approach, and clustering framework are now clearly defined prior to their application. This ensures that the analytical approach is introduced within the scope of the study before being interpreted in the Results section. Second, the PCA-related content in the Results section has been significantly condensed. Redundant explanations of principal component behavior and cluster characteristics have been removed, and the discussion has been limited to the interpretation of the projection and its implications for scenario classification. The revised section now focuses on how PCA complements the resilience framework rather than introducing an independent analytical layer.

**26 ^th^ Comment: Again, the methods about dynamic lighting control in the middle of results!**

**Suggested Reply: **In the revised manuscript, the methodological framework of the dynamic lighting control strategy has been fully transferred to the Methodology section (new Section 3.8: *Dynamic Lighting Control on Energy and Crop Cycle Duration*). All threshold definitions for low-PV (“cloudy”) day detection, regression relationships linking photoperiod reduction to crop cycle extension, and the corresponding mathematical formulations (Equations 7–10) are now clearly introduced prior to their application. The Results section (Section 4.5) has been revised accordingly and now focuses exclusively on the interpretation of scenario-level flexibility outcomes and cluster-based performance comparisons, without introducing new analytical formulations.

**27 ^th^ Comment:** **Table 7: The first study does not have the source. This should not be in Results section if you don’t plan to compare with the outcomes of your study.**

**Suggested Reply: **The first study presented in Table 7 is properly referenced, as indicated in the “Source” column ([3]). The three production systems (vertical farming, greenhouse, and open-field) correspond to the same referenced study and are therefore grouped under the same citation. No unreferenced data are included in the table.

**28 ^th^ Comment:** **Figure 18 is very useful**

**Suggested Reply:** We sincerely thank the reviewer for this positive feedback. Figure 18 was intentionally designed to provide a clear comparative visualization of the modeled carbon footprint results against literature benchmarks, thereby facilitating direct interpretation of the environmental performance of the examined scenarios. We are pleased that the reviewer found it useful in supporting the overall analysis and conclusions of the study.

**29 ^th^ Comment:** **General comment for the whole manuscript: remove titles from all your figures.**

**Suggested Reply:** Αll in-figure titles have been removed from the manuscript.

**30 ^th^ Comment:** **General comment for the whole manuscript: The text is too long with a lot of excessive and redundant content. You have described your methodology, results, and approach taken to produce the results multiple times with different sentences. This is not a standard method of writing.**

**Suggested Reply: **The entire manuscript has been carefully revised to improve conciseness and eliminate redundant descriptions.

**31 ^st^ Comment: General comment for the whole manuscript: Avoid using — in the text which is basically introduced by ChatGPT.**

**Suggested Reply: **All **— **where replaced.

**32 ^nd^ Comment: General comment for the whole manuscript: Although they are minor but are important. You must review the whole manuscript to address the grammatical issues with the text. For example: “on the (Table 1)” must be “in Table 1”, and so on.**

**Suggested Reply: **The entire manuscript has been carefully reviewed to correct grammatical and stylistic inconsistencies****

**33 ^rd ^Comment: I’m not sure if you intendedly have provided descriptive text of figures at the last paragraph of each section or it has happened during the submission process. It is highly recommended to solve this issue and bring all descriptions right after where you cite the figure for first time.**

**Suggested Reply: **The manuscript has been carefully revised so that all figure descriptions now appear immediately after their first citation in the text. Redundant or misplaced descriptive paragraphs at the end of sections have been removed or relocated accordingly.

# Reviewer report: Reviewer response for version 1

**DOI:** 10.21956/openreseurope.22555.r62490

**Published:** 2025-11-27

**Authors:** Adnan Arshad

**Affiliations:** 1 Lanzhou University, Lanzhou, China

1. The current title is descriptive but could be more dynamic and precise to immediately capture the reader's interest and better reflect the paper's novel contributions. "Dynamic Energy Optimization in Vertical Farming: A Multi-Scenario Simulation Framework for Climate-Resilient and Low-Carbon Food Production". 

 2. The abstract effectively summarizes the work but can be strengthened by more explicitly stating the research gap and the novelty of the proposed metrics. 

 3. Explicitly stating the lack of a "comprehensive framework" in the background justifies the study's necessity.

 4. Adding words like "novel" and "as a key innovation" helps flag the paper's original contributions (the new metrics and classification) to time-pressed readers and reviewers.

 5. Minor tweaks like changing "life cycle and CO2 emissions" to "life cycle CO2 emissions" and adding "system" after "classification" improve readability and precision.

 6. Clarify how the dynamic crop cycle model specifically translates environmental conditions (e.g., light integral from the varied photoperiods, temperature from HVAC setpoints) into a quantitative yield prediction (in kg) for each of the 48 scenarios?

 7. Clarify which of these temperature regimes was used as the baseline or setpoint for your 48 operational scenarios? Furthermore, what was the scientific rationale—based on the cited literature or other sources—for selecting one range over the other in defining your model's "optimal" conditions?

 8. Revise figures, fig 2, dimensions are not visible 

 9. You mention that the HVACD system was modeled within TRNSYS 18. Could you please detail the specific control logic implemented for the dehumidification (D) component?

 10. What was the specific relative humidity setpoint or control band used to activate dehumidification?

 11. How was the latent heat load from the plants and the NFT system quantified and input into the simulation?

 -How was the significant internal heat gain from the LED lighting explicitly integrated into the calculation of the HVAC heating and cooling loads within the TRNSYS model?

 -

 You state that surplus PV energy was "curtailed or stored locally." For scenarios with battery storage, how was the *grid electricity consumption* specifically calculated? Was it the energy drawn directly from the grid after the PV and battery system failed to meet the load, or was a specific charging/discharging efficiency and degradation factor for the battery accounted for when determining the net grid energy import over the annual cycle? This directly affects the accuracy of the core emissions calculation.

 -Revise figures and improve quality. 

 -Fig. 9  add trend line

 -The threshold for identifying "cloudy days" is defined using two different criteria: 1) days below the 10th percentile of annual PV generation, and 2) days more than one standard deviation below the seasonal average.

 Could you clarify which of these two criteria was ultimately used to trigger the dynamic lighting reductions? The choice between an annual percentile and a seasonally-adjusted standard deviation would identify significantly different sets of days, potentially leading to different outcomes in both energy savings and the impact on crop cycle duration.

Is the study design appropriate and does the work have academic merit?

Yes

Is the work clearly and accurately presented and does it cite the current literature?

Yes

If applicable, is the statistical analysis and its interpretation appropriate?

Yes

Are all the source data underlying the results available to ensure full reproducibility?

Yes

Are the conclusions drawn adequately supported by the results?

Partly

Are sufficient details of methods and analysis provided to allow replication by others?

Yes

Reviewer Expertise:

Climate-smart agriculture, digital agronomy

I confirm that I have read this submission and believe that I have an appropriate level of expertise to confirm that it is of an acceptable scientific standard, however I have significant reservations, as outlined above.

# Response

**Published:** 2026-03-10

**Authors:** Chrysovalantis Ketikidis

**1 ^st ^Comment: **The current title is descriptive but could be more dynamic and precise to immediately capture the reader's interest and better reflect the paper's novel contributions. "Dynamic Energy Optimization in Vertical Farming: A Multi-Scenario Simulation Framework for Climate-Resilient and Low-Carbon Food Production

**Suggested Reply: **The title was revised to be more dynamic and to better highlight the paper’s novel contributions. In particular, the revised title explicitly references the proposed Lighting Flexibility Classification and the TRNSYS-based simulation framework, which constitute key methodological innovations of the study.

**2 ^nd^ Comment: **The abstract effectively summarizes the work but can be strengthened by more explicitly stating the research gap and the novelty of the proposed metrics.

**Suggested Reply: **The abstract has been revised to explicitly articulate the research gap addressed by this study, namely the lack of a comprehensive framework linking seasonal renewable availability, crop cycle scheduling, and operational flexibility in vertical farming. In addition, the novelty of the proposed performance metrics and the Lighting Flexibility Classification system is now clearly highlighted to guide readers toward the paper’s original contributions.                

**3 ^rd^ Comment: **Explicitly stating the lack of a "comprehensive framework" in the background justifies the study's necessity.

**Suggested Reply: **The Background of the abstract was revised to explicitly state the lack of a comprehensive framework in existing studies, thereby strengthening the justification and necessity of the present work.                

**4 ^th^ Comment: **Adding words like "novel" and "as a key innovation" helps flag the paper's original contributions (the new metrics and classification) to time-pressed readers and reviewers.

**Suggested Reply: **Explicit terminology was added to the abstract to clearly flag the paper’s original contributions, including the proposed performance metrics and the Lighting Flexibility Classification system.                

**5 ^th^ Comment: **Minor tweaks like changing "life cycle and CO2 emissions" to "life cycle CO2 emissions" and adding "system" after "classification" improve readability and precision **.** **Suggested Reply: **Changes were made in the paper.                

**6 ^th^ Comment: **Clarify how the dynamic crop cycle model specifically translates environmental conditions (e.g., light integral from the varied photoperiods, temperature from HVAC setpoints) into a quantitative yield prediction (in kg) for each of the 48 scenarios?

**Suggested Reply: **The dynamic crop cycle model was designed to estimate cultivation cycle duration as a function of environmental control parameters (photoperiod and temperature setpoints), rather than to explicitly simulate biomass accumulation. Harvested yield per cultivation cycle (kg) was assumed constant across all scenarios, based on pilot-scale operation data and literature-reported fresh weight at physiological maturity for lettuce grown under controlled PPFD conditions. Environmental parameters therefore influence quantitative yield indicators indirectly, by modifying crop cycle duration and seasonal timing, which in turn affect total energy consumption and life-cycle CO₂ emissions per kilogram of harvested lettuce. This clarification has been added to the Methodology section.                

**7 ^th^ Comment: **Clarify which of these temperature regimes was used as the baseline or setpoint for your 48 operational scenarios? Furthermore, what was the scientific rationale—based on the cited literature or other sources—for selecting one range over the other in defining your model's "optimal" conditions?

**Suggested Reply: **We did not adopt a single temperature regime as a baseline. Instead, the 48 scenarios span a literature-based operational envelope for lettuce cultivation in vertical farming. Heating (12–18 °C) and cooling (20–28 °C) setpoints were selected based on reported optimal and tolerance ranges in controlled-environment agriculture studies. This design choice enables the assessment of energy and resilience trade-offs across realistic operational conditions, rather than benchmarking against a single assumed optimal case.****

**8 ^th^ Comment: **Revise figures, fig 2, dimensions are not visible

**Suggested Reply: **Figure 2 was revised so dimensions are visible.                

**9 ^th^ Comment: **You mention that the HVACD system was modeled within TRNSYS 18. Could you please detail the specific control logic implemented for the dehumidification (D) component?

**Suggested Reply: **Dehumidification was modeled implicitly as part of the HVAC operation in the single-zone TRNSYS Type 56 model. A relative humidity control band was defined, and when the upper humidity threshold was exceeded, moisture removal was achieved through latent heat removal via the cooling system. No standalone dehumidifier was explicitly modeled; instead, dehumidification energy demand was included in the HVAC cooling load, as calculated by TRNSYS based on zone moisture balance. This clarification has been added to the Methodology section.                

**10 ^th^ Comment: **What was the specific relative humidity setpoint or control band used to activate dehumidification?

**Suggested Reply: **Relative humidity was controlled using an upper-limit control strategy. An indoor relative humidity control band of approximately 65–75% was applied, and dehumidification was activated when the upper threshold was exceeded. Moisture removal was achieved via latent heat removal through the HVAC cooling operation, and the associated energy demand was included in the cooling load. This information has been clarified in the Methodology section.                

**11a ^th^ Comment: How was the latent heat load from the plants and the NFT system quantified and input into the simulation?**

**Suggested Reply: **Latent heat loads were quantified based on plant transpiration rather than the NFT system itself. Transpiration rates were taken from literature for indoor lettuce cultivation (0.2–0.5 L plant⁻¹ day⁻¹), and the corresponding latent heat was calculated using the latent heat of vaporization of water. This latent load was implemented as an internal latent gain within TRNSYS Type 56, enabling the dehumidification component of the HVACD system to respond dynamically. The NFT system was not modeled as a separate latent source, since moisture release originates from plant transpiration.                

**11b ^th^ Comment: How was the significant internal heat gain from the LED lighting explicitly integrated into the calculation of the HVAC heating and cooling loads within the TRNSYS model?**

**Suggested Reply:** The electrical power consumption of the LED lighting system was fully treated as internal sensible heat gain within the single-zone Type 56 model. This heat gain was explicitly included in the zone energy balance and directly influenced both heating and cooling loads calculated by the HVAC system. Lighting power profiles varied by scenario according to the defined photoperiods, ensuring that the thermal impact of lighting was dynamically captured throughout the simulations.

**12 ^th^ Comment: You state that surplus PV energy was "curtailed or stored locally." For scenarios with battery storage, how was the *grid electricity consumption* specifically calculated? Was it the energy drawn directly from the grid after the PV and battery system failed to meet the load, or was a specific charging/discharging efficiency and degradation factor for the battery accounted for when determining the net grid energy import over the annual cycle? This directly affects the accuracy of the core emissions calculation.**

**Suggested Reply: **Grid electricity consumption was obtained directly from the quarter-hourly energy flow outputs of the TRNSYS model and represents the portion of electrical demand not covered by on-site PV generation and locally stored energy. Under the adopted self-consumption scheme, PV energy was prioritized to supply on-site loads, while surplus energy was stored or curtailed, with no feed-in to the grid assumed. Annual grid electricity consumption was then used for the CO₂ emissions calculation. Battery degradation was not included, as the study focuses on operational performance over an annual time horizon. The manuscript has been clarified accordingly.                

**13 ^th^ Comment: Revise figures and improve quality**.

**Suggested Reply: **All figures have been revised and exported to meet the minimum required quality standards. Specifically, all images are provided at a resolution of at least 300 dpi, with improved clarity of labels, axes, and legends to ensure readability in both digital and print formats. No changes were made to the underlying data or results.

**14 ^th^ Comment: Fig. 9 add trend line**

**Suggested Reply: **Figure 9 has been updated to include a linear trend line. The added dashed line highlights the overall increase in grid dependency across scenarios, improving visual interpretation without altering the underlying data.

**15 ^th^ Comment: The threshold for identifying "cloudy days" is defined using two different criteria: 1) days below the 10th percentile of annual PV generation, and 2) days more than one standard deviation below the seasonal average. Could you clarify which of these two criteria was ultimately used to trigger the dynamic lighting reductions? The choice between an annual percentile and a seasonally-adjusted standard deviation would identify significantly different sets of days, potentially leading to different outcomes in both energy savings and the impact on crop cycle duration.**

**Suggested Reply: **The final implementation of the dynamic lighting control strategy, a single primary operational criterion was used to trigger lighting reductions. Specifically, cloudy days were identified as days with daily PV generation below the 10th percentile of the annual PV production profile. This percentile-based threshold was selected to ensure a consistent and conservative identification of low-solar-availability days across the entire year. Deviations greater than one standard deviation below the seasonal average were monitored as a supplementary indicator of low-solar conditions during preliminary analysis, but they did not independently trigger lighting reductions in the final simulations. The manuscript has been revised to clarify this distinction and improve methodological transparency.
